# Spin annihilations of and spin sifters for transverse electric and transverse magnetic waves in co- and counter-rotations

**DOI:** 10.3762/bjnano.5.199

**Published:** 2014-10-28

**Authors:** Hyoung-In Lee, Jinsik Mok

**Affiliations:** 1 Research Institute of Mathematics, Seoul National University, Seoul, 151-747 Korea; 2 School of Computational Sciences, Korea Institute for Advanced Study, Seoul Korea; 3 Dept. of Mathematics, Sunmoon University, Asan, Chungnam 336-708 Korea

**Keywords:** angular momentum, multiplexing, nanoparticle, orbital, Poynting, spin, trajectory

## Abstract

This study is motivated in part to better understand multiplexing in wireless communications, which employs photons carrying varying angular momenta. In particular, we examine both transverse electric (TE) and transverse magnetic (TM) waves in either co-rotations or counter-rotations. To this goal, we analyze both Poynting-vector flows and orbital and spin parts of the energy flow density for the combined fields. Consequently, we find not only enhancements but also cancellations between the two modes. To our surprise, the photon spins in the azimuthal direction exhibit a complete annihilation for the counter-rotational case even if the intensities of the colliding waves are of different magnitudes. In contrast, the orbital flow density disappears only if the two intensities satisfy a certain ratio. In addition, the concepts of spin sifters and enantiomer sorting are illustrated.

## Introduction

Electromagnetic (EM) waves are now fairly well understood at least in terms of angular momentum (AM) and Poynting vector (PV). For instance, the AM of spin-one photons is divisible into the spin and orbital parts [1–6]. Because the PV is proportional to the flow density of the total linear momentum, the examination of the PV flows, i.e., their trajectories would reveal the secrets behind the AM [6]. The AM is revealed, for instance, in the optical vortices with circular and helical streamlines in two and three spatial dimensions, respectively. As prototypically non-rectilinear propagations [3,5–6], the circular propagations can be further divided into clock-wise and counter-clockwise ones. When translated into propagations in three-dimensional space, we call them right- or left-handed helical propagations.

**Figure 1:**
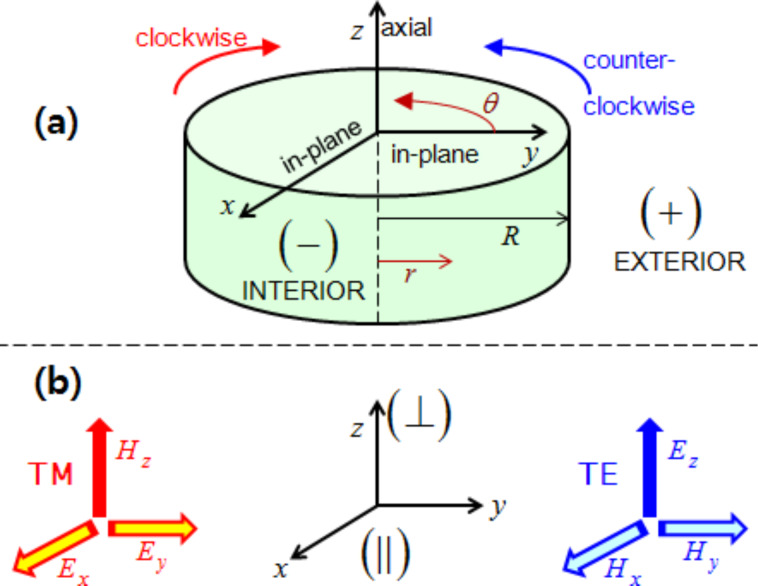
(a) The axial direction is along the *z*-axis, whereas the "in-plane" refers to the *xy*-plane. The superscript "−" refers to the interior of a cylinder, whereas the superscript "+" refers to the exterior. A thin layer lies between the interior and exterior dielectric media. (b) A schematic for the TE and TM waves.

Closely related to the photon spins are those metamaterials that consist exclusively of nonmagnetic constituents [7–12]. For example, a simple planar periodic array of split-ring resonators (SRRs) provides magnetic resonances. Of particular interest is a stacked SRR dimer, in which the relative twist angle between the dimer constituents tunes the degree of interference between the electric and magnetic modes [6,10–11]. This varied interference turns out to be related to the co- and counter-rotations under current investigation as presented in Figure 1a. As a reference, closed rings lack a magnetic resonance mode in comparison to split rings. In addition, the enantiomer sorting takes advantage of the optical chirality and helical flows which accompany the photon spins [5,12].

Another important area in which AM-carrying beams prove useful is the manipulation of nanoparticles. Here, particles with electric polarizability experience appreciable forces by the illuminating EM waves [4,13]. For instance, nanoparticles in a semi-infinite space undergo the influence of the AM of the beams, which are incident through prisms from the other semi-infinite space. Here, evanescent waves with a spatial inhomogeneity or of structured waves play an important role in supplying such AM [6]. Based on the above two kinds of examples, we conclude in loose terms that the spatial inhomogeneity is essential to imparting the AM of EM waves to nanoscale objects [3,14].

Our investigation is motivated to better understand multiplexing of EM waves in the area of wireless communications [15], which is among numerous applications of photon AM. Beams that carry different angular momenta are multiplexed, transported, and then recovered. The communication capacity is thus enhanced, although there remain many obstacles to practical realizations. Here, suitable spiral phase plates (SPPs) help to generate the necessary AM to the beams [16], by imposing either clockwise or counter-clockwise rotational AM. These SPPs must be excited in turn by either electric or optical means. Of course, practical SPPs are associated with three-dimensional inhomogeneity.

We focus here on the simplest multiplexing, namely, a duplexing between two beams: the transverse-electric (TE) and the transverse-magnetic (TM) fields as shown in Figure 1b. However, TE and TM waves in co-rotations have the same azimuthal mode index, thereby referring to the same AM. Instead, we concentrate here on the TE and TM waves in counter-rotations, thus referring to the opposite azimuthal mode indices [15,17]. By duplexing, an interference is implied, which is also present in the interactions among multiple beams [3–4,6,12]. In the mathematical context, there turn out to be frequent sign changes in the energy terms for the counter-rotational case in comparison to those for the co-rotational case. These sign changes are related not only to the Coriolis force hidden behind the vector Laplacian in classical fluid dynamics [18] but also to the vector potential leading to the Landau levels in quantum mechanics [19].

Let us refer again to the schematic Figure 1a in both Cartesian and cylindrical coordinates, where a cylinder located at *r* = *R* divides the interior from exterior. Figure 1b displays the TE and TM waves, each being characterized by non-zero axial fields. Both TE and TM waves are assumed to undergo circular propagations in the *xy*-plane. Hence, the TM and TE modes correspond, respectively, to the S- and P-polarizations [7]. This annular cylinder of infinitesimally small thickness idealizes a possible active zone of optical gain [20–22]. The current study complements our previous one on the co-rotating TE and TM waves [23]. In [23], we have paid a little more attention to the jump conditions across the cylindrical active layer, which essentially provides energy to the PV flows in both the interior and the exterior [20].

In the current study, we will just take the annular cylinder as a black box. Therefore, our forthcoming formulations are relatively scale-independent. As an example from the current technological perspective [7–9], we assume a mid-IR EM wave of a frequency of 3 THz. The diameter of the cylinder shown in Figure 1a is one wavelength of 100 μm. Assuming the thin layer of thickness as small as its hundredth refers to 1 micrometer. In this respect, nanoparticles immersed in this configuration could be appropriately treated. From theoretical and numerical analysis, we found that the results from the counter-rotational case are not simply opposite to those from the co-rotational case. In other words, the mutual reinforcements and cancellations accompanying the counter-rotational case are so subtle as to defy ordinary expectations gleaned from our previous results for the co-rotational case in [23]. In particular, the photon spins turn out be quite intriguing [15,20,23].

This paper is organized as follows. Section "Formulation and Fundamentals" describes a problem statement and solutions to such formulations. Section "Poynting Vectors and Trajectories" considers Poynting vectors and associated trajectories. Section "Angular Momentum and Spins" considers the AM and photon spins, thus coming up with several extraordinary concepts. Section "Discussion" provides additional arguments, followed by "Conclusion" summarizing the principle behind spin sifters and other issues.

## Results

### Formulation and fundamentals

The first part of this section up to Equation 1 largely follows what has been presented in our paper [23]. Nonetheless, more details are provided in Section S1 of Supporting Information File 1. As shown in Figure 1a for the Cartesian coordinates (*x*,*y*,*z*), the cylindrical coordinates (*r*,θ,*z*) are related by (*x*,*y*) = *r* (cos θ, sin θ). Both electric field 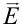 and magnetic field 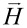 are dependent on (*r*,θ) so that waves propagate only in the in-plane direction normal to the *z*-axis. The TE and TM waves presented in Figure 1b are independent basis states in this cylindrical configuration. As a result, the EM field vectors are decomposable into two orthogonal modes: (i) the TE mode with 
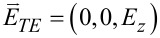
 and 
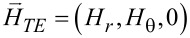
, and (ii) the TM mode with 
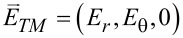
 and 
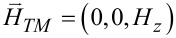
 [4–7]. We assume the time-harmonic and azimuthally periodic form 
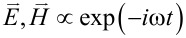
. Here, ω is frequency and *t* is the time, from which k ≡ ω/*c* is the free-space wave number.

Consider the normalized amplitude functions (*f_r_*,*f*_θ_,*f_z_*) and (*h_r_*,*h*_θ_,*h_z_*) for the electric and magnetic fields, respectively. The total fields are then expressed as below for the counter-rotational case [3]:

[2]
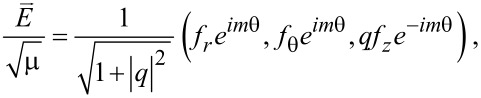


[3]
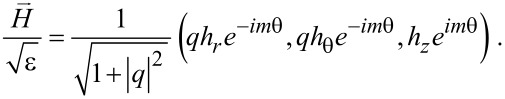


Here, the complex number *q* is the coupling coefficient between the TE and TM modes [4]. Hence, the two limits |*q*| → 0 and |*q*| → ∞ refer, respectively, to the pure TM and pure TE modes. Besides, |*q*|^2^ ≡ q*·*q*, and ε is the permittivity, which is positive for dielectric media so that ε ≡ *n*^2^ with *n* being the refractive index. In addition, μ = 1 is the permeability for non-magnetic materials. Note that both ε and μ have already been made dimensionless [2–3].

Equally important is that the integer *m* is the azimuthal mode index. For the co-rotational case, all the occurrences of exp(±*im*θ) in both Equation 2 and Equation 3 should be replaced by either exp(*im*θ) or exp(−*im*θ) [23]. Therefore, the TE and TM waves in Equation 2 and Equation 3 follow the respective proportionalities exp[−*i*(*m*θ + ω*t*)] and exp[*i*(*m*θ − ω*t*)], thus signifying counter-rotating propagations [15,20]. Consider the Maxwell's equations 
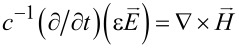
 and 
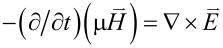
, with *c* as the speed of light. The variables in the interior and exterior are denoted by superscripts "−" and "+", respectively. For instance, the electric permittivity is denoted by ε^±^. For the sake of simplicity, we keep constant in this study *n*^−^ = 2 and *n*^+^ = 1 (vacuum). Furthermore, we introduce normalized variables and operators: ρ ≡ *kr* and 
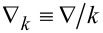
. Hence, the Maxwell's equations become 
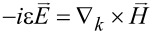
 and 
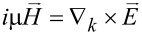
, thus expressing the in-plane components in terms of the axial ones.

With the Helmholtz operator 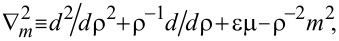 both axial field components are governed by 
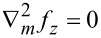
 and 
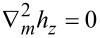
. Across the thin layer, the interface conditions for the fields lead to 
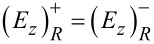
 and 
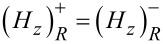
. In addition, the analysis across the thin layer provides us with another set of jump conditions: 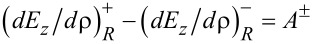 and 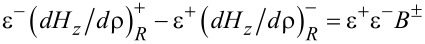 [20–21], where the two parameters *A*^±^ and B^±^ account for what happens within the thin layer at *r* = *R* as indicated in Figure 1a. Although we are not concerned with the second set of jump conditions as in [22], there is a more serious argument about the possible electrodynamics within the thin layer in [23].

Now we introduce 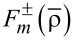 through

[4]
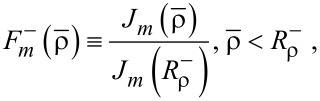


[5]
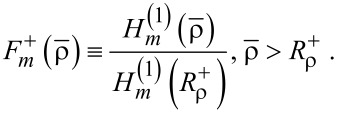


It is convenient to define a new set of radial coordinates 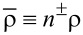, for the interior and the exterior. Then, *J_m_*(n^−^ρ) is the Bessel function of the first order for the interior, whereas 
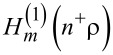
 is the Hankel function of first order for the exterior [1–2,22]. Additionally, we redefine the thin-layer position from the viewpoint of 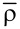 such that 
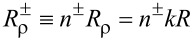
. Therefore, we have the respective ranges of validity: 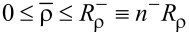 and 
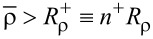
. Second, we introduce the gradient functions of the first and second order:

[6]
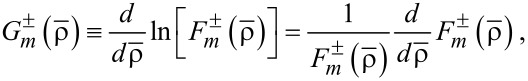


[7]
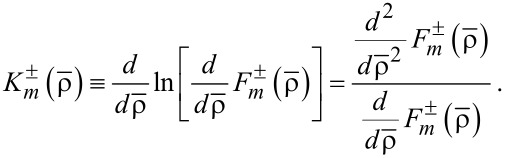


See Figure S1.1 and Figure S1.2 of Supporting Information File 1 for the graphs of 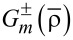 and 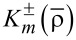. Solely with the two continuity relations 
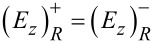
 and 
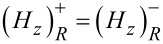
, we arrive at a pair of solutions 
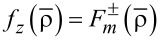
 and 
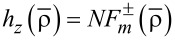
 to the desired axial fields [22]. Here, the refractive-index ratio *N* is defined by:

[1]
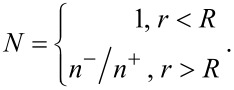


For our data of *n*^−^ = 2 and *n*^+^ = 1, *N* = 1 and *N* = 2 in the interior and the exterior, respectively. This kind of ratio plays a crucial role in stereometamaterials [10].

Consider the EM energy density, *w*, defined by 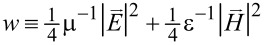 [2,4]. According to Equation 2 and Equation 3, we find from Equations 3–5:

[8]
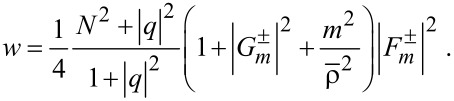


**Figure 2:**
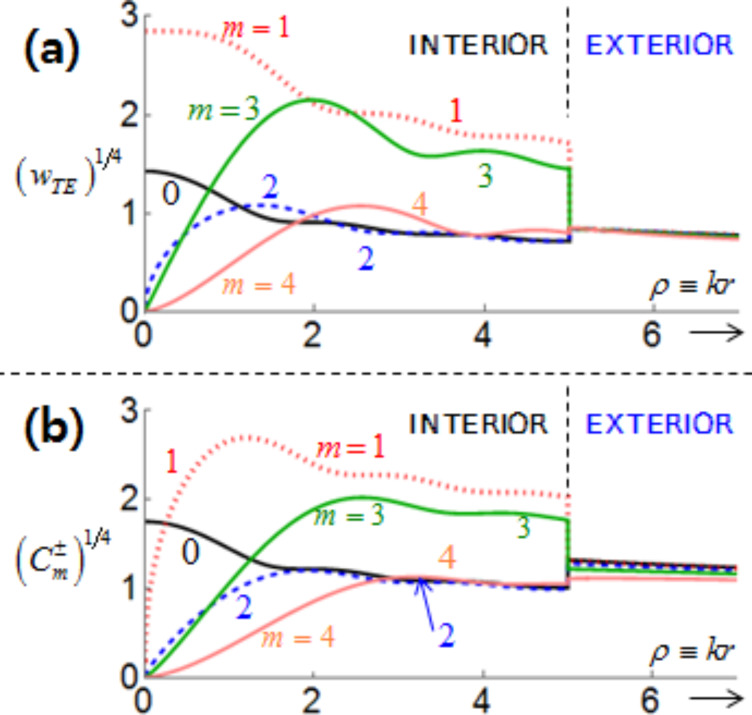
(a) Energy density 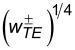. (b) The scaled coefficient 
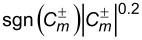
 of the optical chirality *C* for the counter-rotational case. Both are displayed against ρ ≡ *kr* ≡ ω·*r*/*c*. The data are *R*_ρ_ ≡ *kR* ≡ 5 and *m* = 0,1,2,3,4.

Hence, *w* can be separated into the TE and TM modes [1,3]. See Section S2 of Supporting Information File 1 for details of its derivation. Note that the energy density stays the same for both rotational cases. Figure 2a shows the energy density 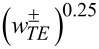 with 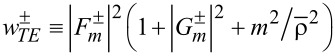 plotted against ρ ≡ *kr* ≡ ω·*r*/*c* with *R*_ρ_ ≡ *kR* ≡ 5 for *m* = 0,1,2,3,4. Here, 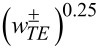 is plotted instead of 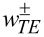 scaled for better contrast. For readers comfortable with the un-scaled 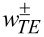, its plot is provided in Section S2 of Supporting Information File 1, in which it turns out that 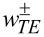 is hard to read due to its widely varying magnitude over ρ especially for *m* = 2,4. In addition, consider the "helicity" σ and "chirality" χ [4]:

[9]
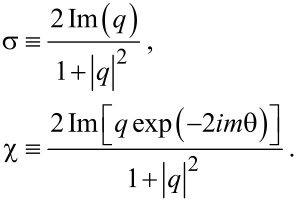


Hence, σ = χ if exp(−2*im*θ) = 1. The optical chirality is 
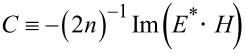
, and it is evaluated to be 
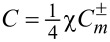
 [12]. Here, the chirality coefficient is defined by

[10]
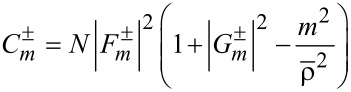


See Section S3 of Supporting Information File 1 for details. As a result, a TE–TM hybrid mode is essential for a non-zero chirality. As a reference, for the co-rotational case we found that 
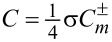
 with 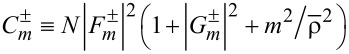, which is thus proportional to the energy density *w* in Equation 8 with regard to the radial dependence.

Figure 2b displays the scaled coefficient 
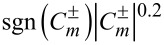
, which is quite similar to *w* in Figure 2a. One notable exception is the curves with *m* = 1 at ρ = 0 in Figure 2, where w ≠ 0 in Figure 2a but 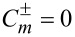 in Figure 2b. With 
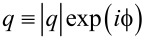
, *q* = 1 refers to axial components of the electric and magnetic fields being parallel, whereas *q* = −1 denotes that the two are anti-parallel. On the other hand, the two axial components are out of phase for *q* = ±*i* [4]. In general, the chirality assumes θ**-**dependent values, for instance, χ ≡ cos(2*m*θ) for *q* = *i* from Equation 9.

**Figure 3:**
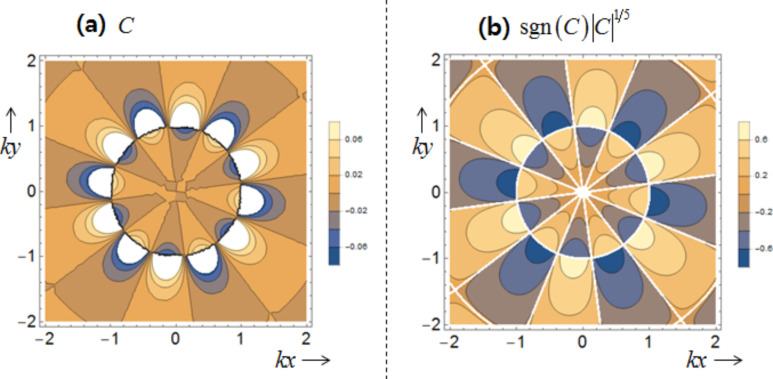
(a) A contour plot of the optical chirality *C*. (b) A contour plot of the scaled optical chirality sgn(*C*)|*C*|^1/5^. Both are plotted in the (*kx*,*ky*)-plane for the counter-rotational case. The data are *R*_ρ_ ≡ *kR* = 1, *m* = 3, and *q* = *i*. We have used the commercial software Mathematica^®^ for drawing these figures so that both negative and positive saturated values are forced to take on the same white color in panel (a). However, one can easily identify the corresponding proper signs from panel (b).

Figure 3 presents contour plots of the unscaled *C* in panel (a), and the scaled sgn(*C*)|*C*|^1/5^ in the (*kx*,*ky*)-plane for the counter-rotational case in panel (b). The data are *R*_ρ_ ≡ *kR* = 1, *m* = 3, and *q* = *i*. The white region in panel (a) signifies extremely high values of *C*. Most of the time the scaled values in panel (b) offer a better overview. Because *m* = 3, the chirality pattern repeats six times per revolution. We clearly observe an inhomogeneous pattern in both radial and azimuthal directions [6]. See Figure S3.1 of Supporting Information File 1 for the corresponding chirality pattern for the co-rotational case, in which only a radial inhomogeneity is observed. See also Figure S3.2 of Supporting Information File 1 for the graphs of sgn(*C*)|*C*|^1/5^ for the counter-rotational case when *m* = 1.

### Poynting vectors and trajectories

The Poynting vector is defined by 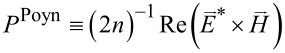. This formula is derived by averaging the energy flow over one temporal cycle for a given frequency, and therefore it applies to both rotational cases [21]. First, we express 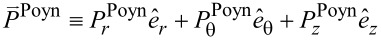 in terms of the field vectors provided in Equation 2 and Equation 3. Second, the resulting formulas are treated in terms of their solutions via Equations 3–5 as follows:

[11]
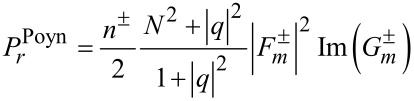


[12]
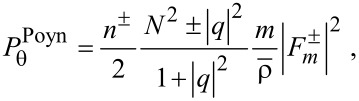


[13]
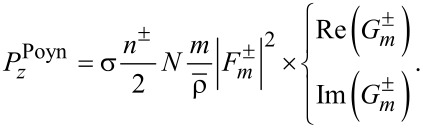


See Section S4 of Supporting Information File 1 for a detailed procedure. For both rotational cases, the radial components remain the same as in Equation 11. That is the centrifugal forces remaining the same for both co-rotating and counter-rotating particles [3,18].

In the numerator *N*^2^ ± |*q*|^2^ of of Equation 12, *N*^2^ + |*q*|^2^ and *N*^2^ − |*q*|^2^ refer to the co- and counter-rotational cases, respectively. As a result, 
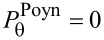
 if |*q*| = *N* from Equation 12 for the counter-rotational case, whereas 
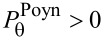
 for the co-rotational case. It is quite natural that the counter-rotational propagations of the TE and TM modes could cancel each other in the azimuthal direction, if the magnitude of their coupling constant |*q*| is equal to the ratio of the dielectric constants *N*. However, according to Equation 1, the special condition |*q*| = *N* does not hold true to both interior and exterior if *N* ≠ 1.

For 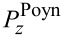 in Equation 13, the last factors 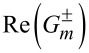 and 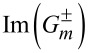 refer to the co- and counter-rotational cases, respectively. As shown in Figure S1.1 of Supporting Information File 1, we know that 
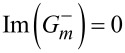
 in the interior, whereas 
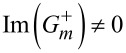
 in the exterior. As a consequence, for the counter-rotational case, 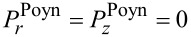 in the interior, whereas they are non-zero in the exterior. For the co-rotational case, 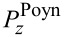 does not vanish both in the interior and exterior, since 
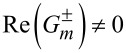
 for all 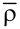 in general. In addition, it is clear that both 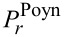 and 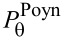 are separable into their correspnding TE and TM modes.

A set of additional characteristics is observed. First from Equation 12 and Equation 13, it is seen that 
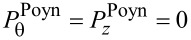
 for *m* = 0, namely, if the state is azimuthally stationary. Second, all the components vanish as ρ → 0 except if *m* = 0. It is because 
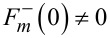
 for *m* = 0, whereas 
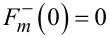
 for *m* > 0. Therefore, there is zero intensity or a phase singularity at the coordinate origin for *m* > 0 [2]. Third, the role of the helicity σ defined in Equation 9 remains the same for both rotational cases. In other words, σ indicates how strong the helical motion is. Lying in the direction normal to the wave propagations, 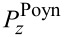 is certainly indicative of spins.

Through 
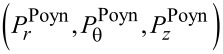
 provided in Equations 11–13, the trajectories traversed by the PVs are easily expressed in terms of 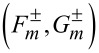 as a function of ρ [22]. First, consider the interior for the counter-rotational case, where 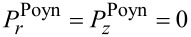. Taken together, the PV flows make two-dimensional circular patterns infinite times [22], according to the trajectory *m*(θ – θ_0_) = *c*(*t* – *t*_0_) with (θ_0_, *t*_0_) constant.

Now consider 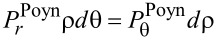 based on Equation 11 and Equation 12, which reads as follows in the exterior:

[14]
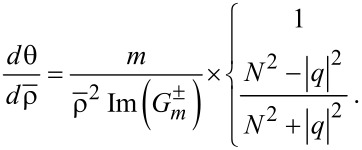


**Figure 4:**
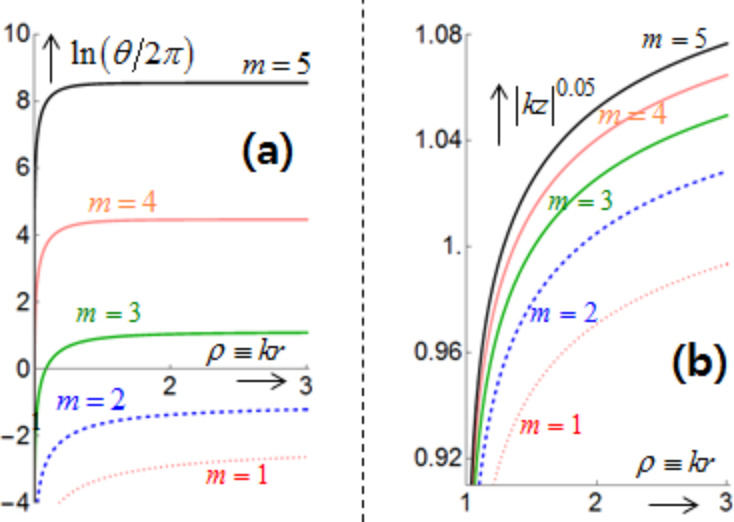
(a) The angle ln(θ/2π). (b) The axial displacement |*kz*|^0.05^ with *q* = *i*. Both are plotted versus ρ ≡ *kr* > 1 in the exterior for *m* = 1,2,3,4,5 and *R*_ρ_ ≡ *kR* = 1.

Here, the upper and lower expressions correspond respectively to the co- and counter-rotational cases. With initial condition θ = 0 at *R*_ρ_ ≡ *kR* = 1, we then numerically integrate Equation 14 to obtain θ(ρ). Note that θ(ρ) depends strongly on |*q*|. Figure 4a shows the resulting ln(θ/2π) versus *n*^+^ρ ≡ *n*^+^*kr*. We thus find that ln(θ/2π) increases sharply with ρ for short distances with ρ > *kR*. Thereafter, ln(θ/2π) approaches a certain limit at a fast rate as ρ → ∞. Besides, Equation 14 indicates no dependence on |*q*| for the co-rotational case. Next, consider 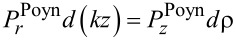 in the axial direction on the basis of Equation 11 and Equation 13, which reads

[15]
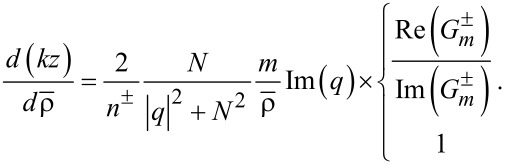


Here, the upper and lower expressions correspond respectively to the co- and counter-rotational cases. With the initial condition *z*(*R*_ρ_) = 0, Equation 15 is analytically integrated to show helical trajectories for the co-rotational case as fully discussed in [23]. In contrast, for the counter-rotational case Equation 15 is integrated to produce 
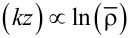
 so that the axial distance increases monotonically with 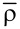 for *m*Im(*q*) > 0. Figure 4b shows (*kz*) versus ρ ≡ *kr* in conformance with the logarithmically increasing trajectories. Both panels of Figure 4 show clearly increasing displacements in both azimuthal and axial directions with higher values of *m*. As regards the dependence on *q* in Equation 15, *q* = ±*iA,* with *A* being a generic real constant, give a pair of trajectories of opposite helical directions, left-handed for *q* = +*iA*, and right-handed for *q* = −*iA*. This feature makes a basis on which to sense and possibly sort out enantiomers [5,10,12].

**Figure 5:**
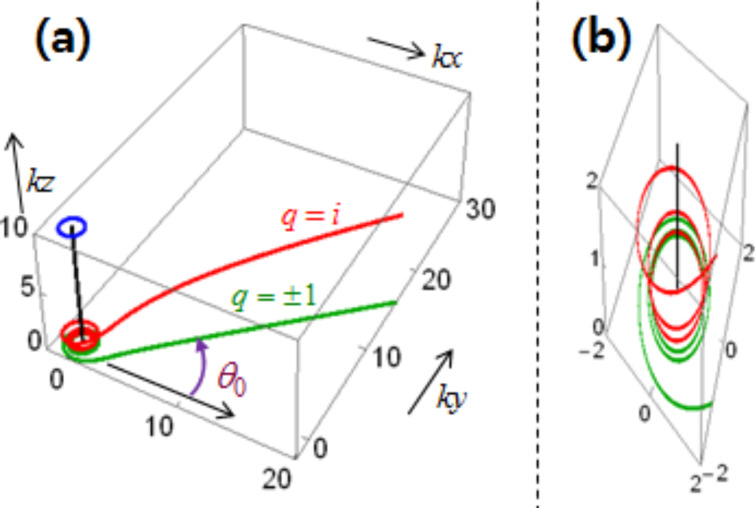
(a) Helical trajectories for the exterior in the (*kx*,*ky*,*kz*)-space. It is given that *R*_ρ_ ≡ *kR* = 1 and *m* = 3. (b) A zoom-in view around the *z*-axis.

Figure 5 displays one set of three-dimensional trajectories in the (*kx*,*ky*,*kz*)-space. Figure 5a shows trajectories over *kR* ≤ *kr* = ρ ≤ 40 for *R*_ρ_ ≡ *kR* = 1 and *m* = 3. For visual aid, we added a straight vertical line at (*x*,*y*) = (0,0) over 0 ≤ *kz* ≤ 10 in black color along with a circle of unit radius at *kz* = 10 in blue color. Now the red curve is the trajectory for *q* = *i*, whereas the green curve is that for *q* = ±1. Incidentally, the latter curve is just the projection of the former onto the *xy*-plane.

From the listed data for Figure 4a, the red curve for *q* = *i* has a value of θ/2π = 3.11 at ρ = 40. The green projected curve reaches an asymptotic line as ρ → ∞, as indicated by the angle θ_0_ ≈ 360° × mod(3.11,1) = 39.7°. The curve for *q* = −*i* lies below the *xy*-plane and is thus not shown. Figure 5b is a zoom-in view of Figure 5a around the origin and the cylindrical axis, which exhibits more clearly the helical trajectory (in red) and its projection (in green) [3,9]. For the co-rotational case, we find similar helical trajectories, which however exist in both interior and exterior as can be inferred from Equation 13, see [23].

### Angular momentum and spins

With the superscript "tot" referring to "total", we define the vector 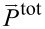 of the total energy flow density (FD) or total linear momentum [3]:

[16]
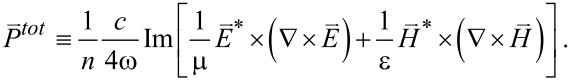


It turns out that 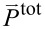 is identical to the Poynting vector 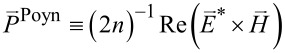 in the dielectric media in either interior or exterior, viz., 
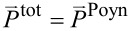
. Moreover, 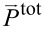 can be decomposed into the orbital (canonical) FD 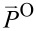 (with the superscript "O") and the spin FD 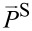 (with the superscript "S"). In other words, 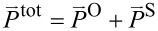 as proved in Section S5 of Supporting Information File 1 [2–5,7], in which not only Maxwell's equations but also several generic vector identities are used. This equality holds true to both rotational cases.

The orbital FD is defined by


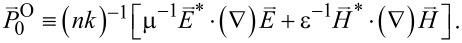


From the fact that the field variables are independent of *z*, we easily deduce that 
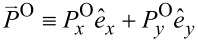
 has only in-plane components without the axial component 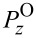. For instance, its electric-field contributions are found as follows with 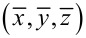 ≡ *nk*(*x*,*y*,*z*).

[17]
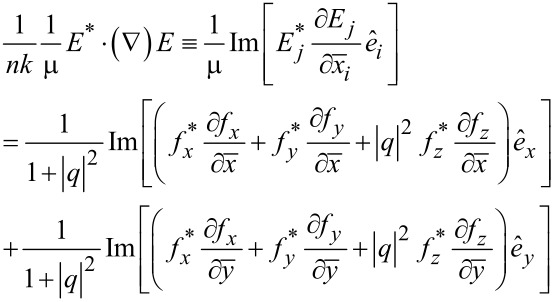


By this way, we go further to express 
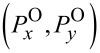
 in terms of (*f_z_*,*h_z_*) and its first- and second-order partial derivatives. See Section S6 of Supporting Information File 1 for detailed derivations and the resulting formulas.

The next step is to find 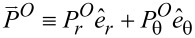 in the polar coordinates through the transformation from Cartesian to polar coordinates. They are

[18]
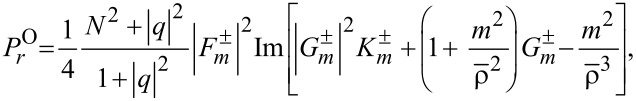


[19]
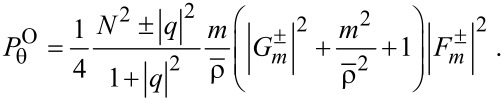


This derivation of 
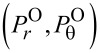
 from 
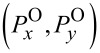
 is rather complicated so that all the details are provided in Sections S7 and S8 of Supporting Information File 1. In particular, the even or odd numbers of differentiations play significant roles in determining the resulting formulas [3,6]. Thus, we find in Equation 18 that 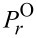 remains the same for both rotational cases. In Equation 19 for 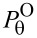, the factors *N*^2^ ± |*q*|^2^ refer respectively to the co- and counter-rotational cases. It is interesting that 
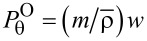
 for the co-rotational case, namely, the azimuthal component of the orbital FD is 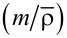-times the energy density *w*, the latter being defined in Equation 8.

As we have done for the Poynting-vector trajectories in Equation 14 [6], we find the following trajectory in polar coordinates from Equation 18 and Equation 19:

[20]
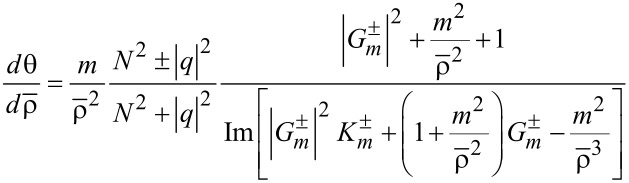


The sign change in *N*^2^ ± |*q*|^2^ takes place only for 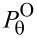 in Equation 19, the orbital FD in the azimuthal direction. This sign change is related to the Coriolis force in the azimuthal direction, when dealing with the vector Laplacian 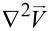 in classical fluid dynamics [18], where 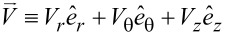 is the velocity vector. It is also related to the vector potential for the kinetic momentum in the Hamiltonian, in which the light-matter interactions lead to the quantized Landau levels in quantum mechanics [19]. See Section S7 of Supporting Information File 1 for details.

**Figure 6:**
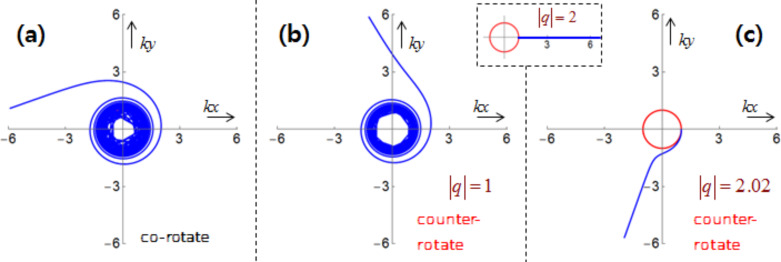
Trajectories of 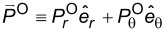 in the (*kx*,*ky*)-plane. (a) The co-rotational case. (b) The counter-rotational case with |*q*| = 1. (inset) The counter-rotational case with |*q*| = 2. (c) The counter-rotational case with |*q*| = 2.02.

As with 
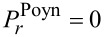
, 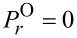 in the interior for the counter-rotational case, as seen from the vanishing denominator of Equation 20. It is because both 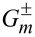 and 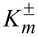 are real numbers in the interior. As a result, the trajectories are just circular. In other words, Equation 20 is valid only in the exterior with 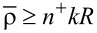. Figure 6 displays the trajectories of 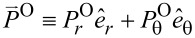 in the (*kx*,*ky*)-plane. All the trajectories are set to start at (*kx*,*ky*) = (1,0) for which *kR* = 1 is specified. Figure 6a shows the co-rotational case in which there is no dependence on *q*. Figure 6b shows the counter-rotational case with |*q*| = 1, whereas Figure 6c shows the counter-rotational case with |*q*| = 2.02. The inset between Figure 6b and Figure 6c shows the counter-rotational case with |*q*| = 2. Since n^−^ = 2 (for a material such as silicon dioxide) and *n*^+^ = 1 (vacuum), *N* ≡ *n*^−^/*n*^+^ = 2. Therefore, the value |*q*| = 2 delineates the neutral state without any propagations in the azimuthal direction as shown by the inset, where the trajectory is just a radial straight line emanating from the starting point.

Figure 6a shows counter-clockwise rotations before reaching an asymptotic azimuthal direction for the co-rotational case [23]. Figure 6b also shows counter-clockwise rotations before reaching another asymptotic azimuthal direction for the counter-rotational case. This counter-clockwise rotation is clear from the factor *N*^2^ − |*q*|^2^ = 2^2^ – 1^2^ = 3 > 0 in Equation 20. In contrast, Figure 6c shows a less-than-one cycle clockwise rotation before reaching yet another asymptotic azimuthal direction for the counter-rotational case. This clockwise rotation is again clear from the factor *N*^2^ − |*q*|^2^ = 2^2^ – 2.02^2^ = −0.0804 < 0 in Equation 20. Figure 4 of [3] will be a good reference as regards such axonometric projections. The red circle of a unit radius on Figure 6c indicates the position of the thin layer between the interior and exterior. From the position of this unit circle, we can tell where the starting circles are situated in both Figure 6a and Figure 6b. It appears in particular on Figure 6a that the initial portions of the trajectory cross this unit circle several times. But, these crossings result from the coarse integration steps taken by the commercial software Mathematica^®^, which we have used for all computations.

The angular momentum (AM) 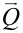 is defined to be the cross product of a position vector 
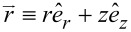
 with the linear momentum 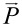 [3–4]. For the orbital part, 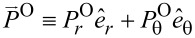, thereby giving rise to 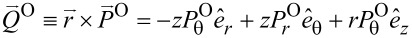.

Let us turn to the spin 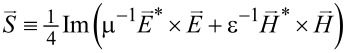 for which the spin flow density (FD) is defined by 
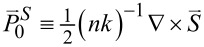
. As derived in full details in Section S9 of Supporting Information File 1, the three components 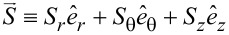 are found as follows:

[21]
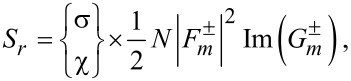


[22]
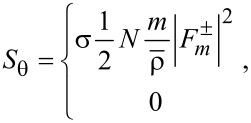


[23]
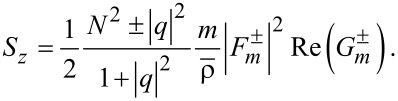


**Figure 7:**
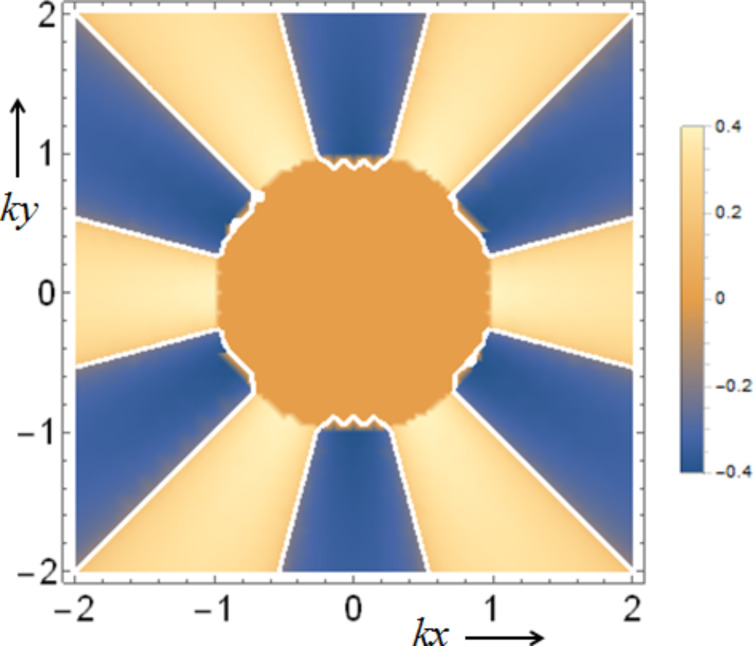
A density plot of the scaled spin in the radial direction sgn(*S_r_*)|*S_r_*|^1/5^ in the (*kx*,*ky*)-plane in the case of a counter-rotation. The data are *R*_ρ_ ≡ *kR* = 1, *m* = 3, and *q* = *i*.

In each component, the upper and lower parts refer to the co- and counter-rotational cases, respectively. First, *S_r_* in Equation 21 for both rotational cases appears to be very similar except for the multiplying factors σ and χ for the co- and counter-rotational cases. From their definitions in Equation 9, the difference between σ and χ is the difference between the factors Im(*q*) and Im[*q* exp(−2*im*θ)] with the latter being dependent on θ. Figure 7 presents a density plot of the scaled spin in the radial direction sgn(*S_r_*)|*S_r_*|^1/5^ in the (*kx*,*ky*)-plane in the case of a counter-rotation. The data are *R*_ρ_ ≡ *kR* = 1, *m* = 3, and *q* = *i*. Because *m* = 3, the chirality pattern repeats six times per revolution. We clearly observe an inhomogeneous pattern in both radial and azimuthal directions. In the interior, we have simply *S_r_* = 0 as seen from the zero level in Figure 7. In the exterior, not only Im(*q*) ≠ 0 but also the spatial inhomogeneity of 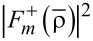 as given in Equation 8 are required for *S_r_* ≠ 0 because of the factor 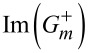 in Equation 21 [4].

Second, 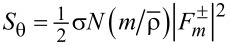 for the co-rotational case, thus being non-zero in general. But, it is surprising that *S*_θ_ = 0 for the counter-rotational case in both interior and exterior. To check this fact, one needs to carefully follow the steps presented in Section S9 of Supporting Information File 1. The fact that *S*_θ_ = 0 for the counter-rotational case is quite understandable from the perspective of the mixed nature of this component. However, we also find it still curious, because the two counter-rotating waves are neither of the same magnitude nor of the same phase in general. In other words, any signature of counter-rotations annihilates the spins in the azimuthal direction, no matter what the relative strength of the counter-rotating fields is [3,6]. As a consequence, the azimuthal component of the spin vector is indicative of whether the TE and TM modes are mixed up or not. This fact gives us a possibility of harnessing the azimuthal spins as binary on-off states rather than continuous signals, with the pair of rotational directions serving as a tuning parameter.

Third, *S_z_* for the co-rotational case has the factor N^2^ + |*q*|^2^, whereas *S_z_* for the counter-rotational case has the factor N^2^ − |*q*|^2^. This fact is similar to the case with 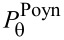, the azimuthal component of the Poynting vector, listed in Equation 12. In fact, *S_z_* is closely related to 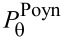 through 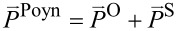 and 
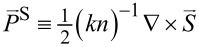
. Focusing on the counter-rotational case, we notice that the helicity-dependent radial components *S_r_* is not separable into the TM and TE modes due to the non-rectilinear in-plane propagations [4], whereas the axial spin component *S_z_* is separable.

**Figure 8:**
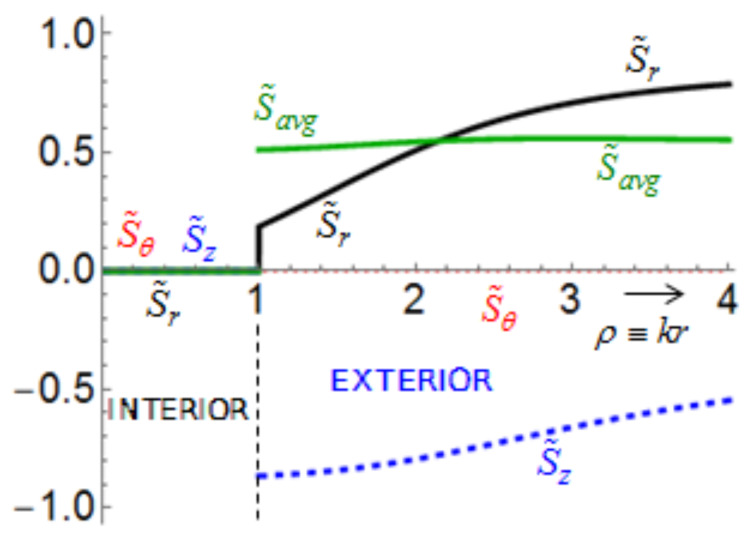
Spin components 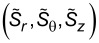 for the counter-rotational case plotted over 0 ≤ ρ ≡ *kr* ≤ 4 for *m* = 3, *R*_ρ_ ≡ *kR* = 1, and *q* = (1 + *i*)/√2. The scaled variable is 
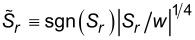
, for instance.

We define the normalized spin 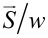 per photon [3]. For a better viewing, we then introduce scaled parameters, for instance, 
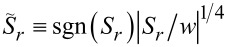
. Figure 8 presents such scaled parameters 
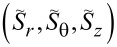
 over 0 ≤ ρ ≡ *kr* ≤ 4 with *R*_ρ_ ≡ *kR* = 1 and *m* = 3. In particular, we provided a non-trivial coupling *q* = (1 + *i*)/√2. Furthermore, we set exp(−2*im*θ) = 1 in Equation 9 for convenience so that σ = χ. We find then through numerical computations that |*S_r_*| < *w* and |*S_z_*| < *w* [1]. The average spin 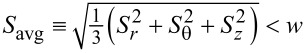 is plotted as the green curve. All the curves in Figure 8 display discontinuities in the radial profiles across the thin layer. From Equation 23, it follows that *S_z_*(ρ) = 0 in the interior for the counter-rotational case, because *N* = 1 from Equation 1 and |*q*| = 1. In comparison, *S_z_*(ρ) < 0 in the exterior. These sudden jumps in the spin components take place due to the thin layer [2], where excitations are supplied. It is obvious that *S*_avg_ is larger in the co-rotational case than that of the counter-rotational case due to *S*_θ_(ρ) = 0 for the counter-rotational case. See Figure S9.1 of Supporting Information File 1 for the graphs of 
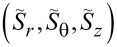
 for the co-rotational case. Figure S9.2–9.4 of Supporting Information File 1 provide further results from parametric studies.

Meanwhile, the spin flow density (FD) is readily computed from 
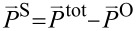
. Because 
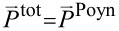
 and 
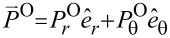
, 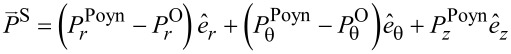 [4–5]. Therefore, 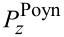 is unique to the spin FD. The spin vector is related to the spin FD through 
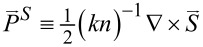
 with *k* ≡ ω/*c*. As a result, 
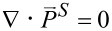
, and the in-plane spin vector 
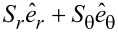
 contributes to 
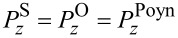
. Similarly to the orbital FD, we can formulate the spin AM 
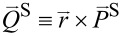
 to be 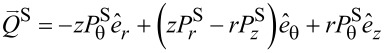 [3].

## Discussion

**Figure 9:**
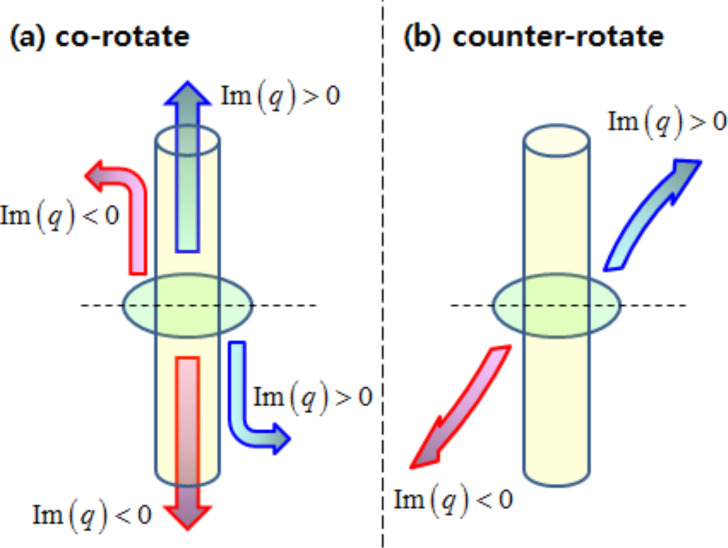
Schematic for the Poynting-vector flows. (a) Co-rotational case. (b) Counter-rotational case. Spin sifters can be devised according to the helicity σ.

For a good part of this study, we have seen the ubiquitous role of Im(*q*) through both helicity σ ≡ 2(1 + |*q*|^2^)^−1^ Im(*q*) and chirality χ ≡ 2(1 + |*q*|^2^)^−1^ Im[*q* exp(−2*im*θ)] in Equation 9. We summarized the behavior of the Poynting-vector (PV) trajectories in Figure 9. Figure 9a is drawn on the basis of Equation 14 and Equation 15 for the co-rotational case, as fully discussed in [23].

Similarly, Figure 9b is drawn on the basis of Equation 14 and Equation 15 for the counter-rotational case. Figure 9b can be inferred from Figure 5 as well. In particular, it is noteworthy that in the interior there are PV flows for the co-rotational case in Figure 9a, but there are none for the counter-rotational case in Figure 9b. In comparison, there are PV flows in the exterior for both rotational cases. However, there are certain asymptotic limits on the axial levels for the co-rotational case as indicated by the sharply turning arrows in Figure 9a. See [23] for detailed discussions for the co-rotational case. In contrast, the ever-progressing levels in Figure 9b indicate either a logarithmic increase or a logarithmic decrease of the axial displacements for the counter-rotational case.

In Figure 9, helical transports along the PV trajectories are hidden behind all the thick arrows. In the exterior in both panels, the PV of photons with *Im*(*q*) > 0 flows axially on one axial side (blue colors). On the other hand, the PV of photons with *Im*(*q*) < 0 flow outward on the opposite axial side (red color). As a consequence, we could activate spin sifters for photons, if proper means for collecting photons are placed at appropriate locations and directions. Presumably, such spin sifters are more efficient for the co-rotational case than for the counter-rotational case, because of the fixed asymptotic axial levels for the co-rotational case. If enantiomers are transported along such PV trajectories, there is a possibility of their being sorted out according to the helicities, namely either upward or downward.

This pair of multiplying factors *N*^2^ ± |*q*|^2^ correspond roughly to the two extreme cases of the coupled SRR dimers [10] for which the twist angle serves as something like *q*. It is more interesting that the Lagrangian energy for the electric mode changes its sign as ±|*q*|^2^ for the TE mode, whereas the Lagrangian energy for the magnetic mode stays the same as in *N*^2^. Consider further the following ratio in its original dimensional terms:

[24]
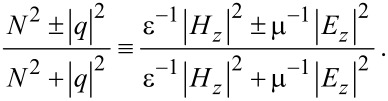


As a result, if we measure the azimuthal component 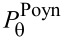 in Equation 12 of the PV in both rotational cases, we could infer the magnetic energy ε^−1^|*H_z_*|^2^ from the measurable electric energy μ^−1^|*E_z_*|^2^. This idea can be traced back to the concept of the Stokes parameters [17]. In addition, because we have solved the Maxwell's equation self-consistently by use of the functions 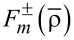, 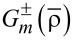, and 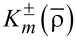 in Equations 3–6, not only the dipoles but also all the higher-order multipoles have been taken into account [10]. In addition, the recurrent appearances of the gradient functions 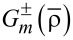 and 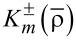 in many places so far corroborate the importance of the spatial inhomogeneity of the structured light.

There is a difference between the artificially structured metamaterials (ASMs) [7] and our artificially structured light (ASL) [6]. In the case of ASM, artificial meta-atoms are fabricated and then put into arrays or clusters, thus forming meta-molecules. In contrast, our ASL in the sense of artificial operating states could be generated from within the thin annular cylindrical zone as in Figure 1a. The thin annular zone may have to be in turn composed of artificially structured (meta)materials with optical gain. Let us count how many levels of key parameters we have. First, the coupling constant *q* accounts for the TE and TM modes. Second, the signs in front of +*m* and −*m* account for the co- and counter-rotations. Third, |*m*| accounts for the rotational strength. Fourth, the superscripts + and − account for the interior and exterior, which refers essentially to a spatial inhomogeneity. Roughly speaking, we have thus a four-level system [6].

For the counter-rotational case, the disappearance of the azimuthal component of spins *S*_θ_ = 0 in Equation 22 was unexpected. We have checked all the details leading to its derivation in Section S9 of Supporting Information File 1 multiple times. To further validate our theoretical results, let us consider a differential counter-rotational case, where the TE and TM waves obey their respective propagation factors as follows.

[25]
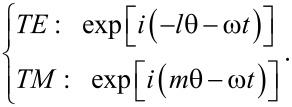


Here, *l,m* > 0 are integers. In words, the TE waves rotate clockwise, whereas TM waves rotate counter-clockwise. The fields variables in Equation 2 and Equation 3 are appropriately modified by employing 
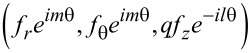
 and 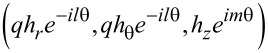 instead of their respective counterparts. In addition, there is one more correction to one of the two Maxwell’s equations, namely, 
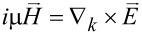
 that involves the space derivatives of the electric field. Once more, all the details for this portion are provided in Section S10 of Supporting Information File 1. We thus end up with the corresponding azimuthal component of spins as follows:

[26]
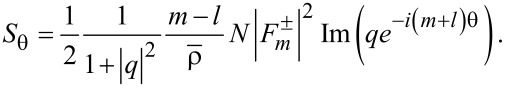


For the counter-rotational case with the same magnitudes of the angular speeds, viz., *l* = *m*, we hence recover *S*_θ_ = 0. It is rather hard to find the difference *m* − *l* in the azimuthal mode indices in various formulas for the photon spins in [3]. See, for instance, Equations (3.39) and (4.9) of [3], although their treatments proceed mostly in the paraxial approximation.

## Conclusion

To conclude, we examined coupled TE and TM waves, which are driven by the dynamics within a thin layer. We focused here on the two waves propagating in counter-rotations, thus revealing various wave characteristics such as both spin and orbital parts of angular momentum. In comparison to our previous results for the co-rotating waves, the enhancements and annihilations caused by the counter-rotations show up in different ways depending on various wave characteristics. In turns out that the azimuthal spin can serve as a probe into the chiral property of photons depending on the co- or counter-rotations. The concept of spin sifters envisaged by the trajectories of the Poynting-vector flows is also differently interpretable depending on the mutual rotational directions.

## Supporting Information

Some of the necessary formulations have already been presented in our preceding study on the co-rotating EM waves [23]. Therefore, in this Supporting Information we present all the detailed solutions not only for the co-rotational case but also for the counter-rotational case. Therefore, we hope that our article is self-consistent without reference to [23].

File 1 Mathematical derivations.
