# Supplementary material for: Spin annihilations of and spin sifters for transverse electric and transverse magnetic waves in co- and counter-rotations
Source: Beilstein J Nanotechnol. 2014 Oct 28;5:1887–98. doi: 10.3762/bjnano.5.199 (PMC4222439; doi:10.3762/bjnano.5.199)
Supplement: File 1 — Mathematical derivations. [file Beilstein_J_Nanotechnol-05-1887-s001.pdf]

Supporting Information

for

Spin annihilations of and spin sifters for transverse electric and transverse magnetic waves in co- and counter-rotations

Hyoung-In Lee*[1,3] and Jinsik Mok[2]

Address: [1]Research Institute of Mathematics, Seoul National University, Seoul, 151-747 Korea, [2]Dept. of Mathematics, Sunmoon University, Asan, Chungnam 336-708 Korea and [3]School of Computational Sciences, Korea Institute for Advanced Study, Seoul Korea

Email: Hyoung-In Lee - hileesam@naver.com
* Corresponding author

Mathematical derivations

This Supporting Information consists of 10 sections. Most of the derivations and discussions are carried out twice: first for the co-rotational case, and second for the counter-rotational case. The purpose of this Supporting Information is to keep all the intermediate steps as detailed as possible, because one is liable to commit mistakes in dealing with complex parameters and variables and their complex conjugates. In particular, the Sections S6-S8 are targeted at finding the orbital part of angular momentum with $\vec{E}^* \cdot (\nabla) \vec{E} \equiv E_j^* \left(\partial E_j / \partial x_i \right) \hat{e}_i$ and $\vec{H}^* \cdot (\nabla) \vec{H} \equiv H_j^* \left(\partial H_j / \partial x_i \right) \hat{e}_i$ in the polar coordinate, which we believe has never been explicitly attempted.

S1. Fundamental Formulas

Consider the Maxwell's equations $-i\varepsilon \vec{E} = \nabla_k \times \vec{H}$ and $i\mu \vec{H} = \nabla_k \times \vec{E}$ with $\nabla_k \equiv k^{-1} \nabla$.

Here, all the field variables follow the proportionality $\exp(-i\omega t)$ for both rotational cases.

For simplicity, consider dielectric media without loss. Both (H_r, H_θ) are thus related to H_z

through $H_r = -i(\mu\rho)^{-1}(\partial E_z/\partial\theta)$ and $H_\theta = i\mu^{-1}\partial E_z/\partial\rho$ for the TE mode. For the TM mode, both (E_r, E_θ) are expressible likewise in terms of E_z through $E_r = i(\varepsilon\rho)^{-1}(\partial H_z/\partial\theta)$ and $E_\theta = -i\varepsilon^{-1} dH_z/d\rho$. Let us group these four relations in the frequency domain by

$$\begin{cases} TE: \quad H_r = -i\dfrac{1}{\mu\rho}\dfrac{\partial E_z}{\partial\theta}, \quad H_\theta = i\dfrac{1}{\mu}\dfrac{\partial E_z}{\partial\rho} \\ TM: \quad E_r = i\dfrac{1}{\varepsilon\rho}\dfrac{\partial H_z}{\partial\theta}, \quad E_\theta = -i\dfrac{1}{\varepsilon}\dfrac{\partial H_z}{\partial\rho} \end{cases}. \tag{S1.1}$$

For the co-rotational case, the combined electric and magnetic fields are hence given below.

$$\begin{cases} \dfrac{\vec{E}}{\sqrt{\mu}} = \dfrac{1}{\sqrt{1+|q|^2}}\left(f_r e^{im\theta}, f_\theta e^{im\theta}, q f_z e^{im\theta}\right) \\ \dfrac{\vec{H}}{\sqrt{\varepsilon}} = \dfrac{1}{\sqrt{1+|q|^2}}\left(q h_r e^{im\theta}, q h_\theta e^{im\theta}, h_z e^{im\theta}\right) \end{cases}. \tag{S1.2}$$

For this co-rotational case, Eq. (S1.1) translate into

$$\begin{cases} TE: \quad h_r = +\dfrac{m}{\bar{\rho}} f_z, \quad h_\theta = i\dfrac{df_z}{d\bar{\rho}} \\ TM: \quad f_r = -\dfrac{m}{\bar{\rho}} h_z, \quad f_\theta = -i\dfrac{dh_z}{d\bar{\rho}} \end{cases}. \tag{S1.3}$$

For the counter-rotational case, the combined electric and magnetic fields are written below.

$$\begin{cases} \dfrac{\vec{E}}{\sqrt{\mu}} = \dfrac{1}{\sqrt{1+|q|^2}}\left(f_r e^{im\theta}, f_\theta e^{im\theta}, q f_z e^{-im\theta}\right) \\ \dfrac{\vec{H}}{\sqrt{\varepsilon}} = \dfrac{1}{\sqrt{1+|q|^2}}\left(q h_r e^{-im\theta}, q h_\theta e^{-im\theta}, h_z e^{im\theta}\right) \end{cases}. \tag{S1.4}$$

For this counter-rotational case, Eq. (S1.1) translate into

$$\begin{cases} TE: \; h_r = -\dfrac{m}{\bar{\rho}} f_z, \;\; h_\theta = i\dfrac{df_z}{d\bar{\rho}}; \\ TM: \;\; f_r = -\dfrac{m}{\bar{\rho}} h_z, \;\; f_\theta = -i\dfrac{dh_z}{d\bar{\rho}} \end{cases}. \qquad \text{(S1.5)}$$

Note the difference that $h_r = (m/\bar{\rho}) f_z$ for the co-rotational TE mode in Eq. (S1.3), whereas $h_r = -(m/\bar{\rho}) f_z$ for the counter-rotational TE mode in Eq. (S1.5). But, the other three relations remain the same.

For a generic complex variable A, we make use of a set of the simple complex-variable identities $\mathrm{Im}(A^*) = -\mathrm{Im}(A)$, $A + A^* = 2\,\mathrm{Re}(A)$, $\mathrm{Re}(iA) = -\mathrm{Im}(A)$, and $\mathrm{Im}(iA) = \mathrm{Re}(A)$ among others. In addition, we need to take caution in taking the following steps $h_\theta^* = \left[i(df_z/d\bar{\rho})\right]^* = -i(df_z^*/d\bar{\rho})$ for $h_\theta = i(df_z/d\bar{\rho})$. Similarly, $f_\theta^* = i(dh_z^*/d\bar{\rho})$ for $f_\theta = -i(dh_z/d\bar{\rho})$. Meanwhile, $|f_z|^2 \equiv f_z^* f_z$ and $|h_z|^2 \equiv h_z^* h_z$.

We recall that the axial profiles $f_z(\bar{\rho}) = F_m^\pm(\bar{\rho})$ and $h_z(\bar{\rho}) = NF_m^\pm(\bar{\rho})$ have been constructed to satisfy the continuity relations $E_z^- = E_z^+$ and $H_z^- = H_z^+$ across the thin layer at $\rho = R_\rho \equiv kR$.

$$\begin{cases} F_m^-(\bar{\rho}) \equiv \dfrac{J_m(\bar{\rho})}{J_m(R_\rho^-)}, \;\; \bar{\rho} < R_\rho^- \\ F_m^+(\bar{\rho}) \equiv \dfrac{H_m^{(1)}(\bar{\rho})}{H_m^{(1)}(R_\rho^+)}, \;\; \bar{\rho} > R_\rho^+ \end{cases}. \qquad \text{(S1.6)}$$

Here, $J_m(\bar{\rho})$ and $H_m^{(1)}(\bar{\rho})$ are the Bessel functions of first kind and Hankel functions of the first kind, respectively.

Thanks to these normalization schemes, it is specified that $F_m^-(\rho = R_\rho) = 1 = F_m^+(\rho = R_\rho)$. In addition, $F_m^\pm(\bar{\rho}) = F_{-m}^\pm(\bar{\rho})$, although $J_m(\bar{\rho}) = (-1)^m J_{-m}(\bar{\rho})$ and

$H_m^{(1)}(\bar{\rho}) = (-1)^m H_{-m}^{(1)}(\bar{\rho})$. This fact $F_m^{\pm}(\bar{\rho}) = F_{-m}^{\pm}(\bar{\rho})$ will be repeatedly employed in dealing with the counter-rotational case. We will also utilize the notation that $F_m^{\pm,*} \equiv \left(F_m^{\pm}\right)^*$.

In addition, we define the first-order gradient function $G_m^{\pm}$ and the second-order gradient function $K_m^{\pm}(\bar{\rho})$ in the radial direction.

$$G_m^{\pm}(\bar{\rho}) \equiv \frac{d}{d\bar{\rho}} \ln\left[F_m^{\pm}(\bar{\rho})\right] = \frac{1}{F_m^{\pm}(\bar{\rho})} \frac{d}{d\bar{\rho}} F_m^{\pm}(\bar{\rho}), \tag{S1.7}$$

$$K_m^{\pm}(\bar{\rho}) \equiv \frac{d}{d\bar{\rho}} \ln\left[\frac{d}{d\bar{\rho}} F_m^{\pm}(\bar{\rho})\right] = \frac{\frac{d^2}{d\bar{\rho}^2} F_m^{\pm}(\bar{\rho})}{\frac{d}{d\bar{\rho}} F_m^{\pm}(\bar{\rho})}. \tag{S1.8}$$

To emphasize that these gradients take non-zero value only for spatially inhomogeneous fields, we may call these gradients "inhomogeneity gradients".

In order to compute $G_m^{\pm}(\bar{\rho})$ and $K_m^{\pm}(\bar{\rho})$, we need to have evaluate derivatives of the Bessel functions of first kind and Hankel functions of the first kind. For the Bessel functions, the recursion relations are

$$\begin{cases} \dfrac{dJ_0(\bar{\rho})}{d\bar{\rho}} = -J_1(\bar{\rho}) \\ \dfrac{d^2 J_0(r)}{dr^2} = -\dfrac{dJ_1(r)}{dr} \end{cases}, \tag{S1.9}$$

$$\begin{cases} \dfrac{dJ_m(\bar{\rho})}{d\bar{\rho}} = J_{m-1}(\bar{\rho}) - \dfrac{m}{\bar{\rho}} J_m(\bar{\rho}) \\ \dfrac{d^2 J_m(\bar{\rho})}{d\bar{\rho}^2} = \dfrac{dJ_{m-1}(\bar{\rho})}{d\bar{\rho}} - \dfrac{m}{\bar{\rho}} \dfrac{dJ_m(\bar{\rho})}{d\bar{\rho}} + \dfrac{m}{\bar{\rho}^2} J_m(\bar{\rho}) \end{cases}, \quad m > 0. \tag{S1.10}$$

The Hankel functions follow a similar set of rules for derivatives. In addition, we need to pay special attentions to the azimuthally stationary case with $m = 0$.

Figure S1.1 presents the radial profiles of $G_m^{\pm}(\bar{\rho})$ plotted against $\rho \equiv kr \equiv \omega r/c$ with

$R_\rho \equiv kR = 1$. In particular, the azimuthally stationary state with $m = 0$ is drawn in a solid black curve. Panels (a) and (b) display $\mathrm{Re}\left(G_m^{\pm}\right)$ and $\mathrm{Im}\left(G_m^{\pm}\right)$, respectively. In particular, the fact that $\mathrm{Im}\left(G_m^{-}\right) = 0$ in the interior will play a dominant role in characterizing various wave properties such as angular momentum and spins.

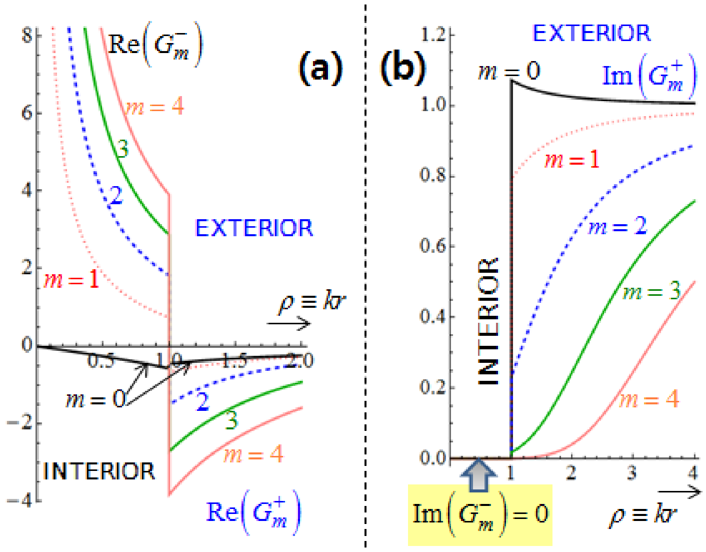

Figure S1.1: The radial profiles of the first-order gradient function $G_m^{\pm}(\rho)$ with the thin layer located at $R_\rho \equiv kR = 1$ and for $m = 0,1,2,3,4$. (a) Real parts. (b) Imaginary parts.

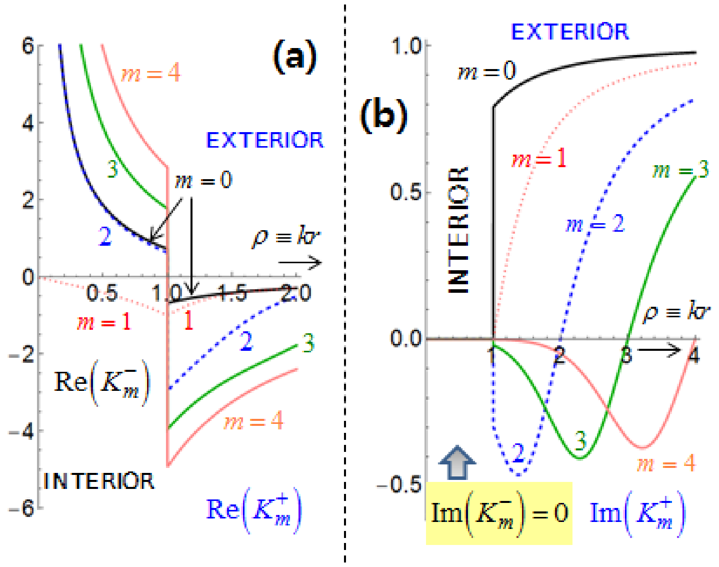

Figure S1.2: The radial profiles of the second-order gradient function $K_m^{\pm}(\rho)$ with the thin layer located at $R_\rho \equiv kR = 1$ and for $m = 0,1,2,3,4$. (a) Real parts. (b) Imaginary parts.

Figure S1.2 displays a similar pair for $K_m^{\pm}(\bar{\rho})$. In short, all the forthcoming formulas end up with expressions involving the functions $F_m^{\pm}(\bar{\rho})$, $G_m^{\pm}(\bar{\rho})$, and $K_m^{\pm}(\bar{\rho})$ provided in Eqs. (S1.6)-(S1.8). In similarity to $\mathrm{Im}\left(G_m^{-}\right)=0$, we notice $\mathrm{Im}\left(K_m^{-}\right)=0$ in the interior, which will be useful for the forthcoming Eq. (S8.24) in evaluating the radial component of the orbital angular momentum.

In addition, we will employ the "helicity" σ and "chirality" χ.

$$\sigma \equiv \frac{2\,\mathrm{Im}(q)}{1+|q|^2}, \quad \chi \equiv \frac{2\,\mathrm{Im}\left[q\exp(-2im\theta)\right]}{1+|q|^2}. \tag{S1.11}$$

Of course, $\chi=\sigma$ for $\exp(-2im\theta)=1$.

S2. Energy Density

The energy density defined by $w \equiv \frac{1}{4}\mu^{-1}\left|\vec{E}\right|^2+\frac{1}{4}\varepsilon^{-1}\left|\vec{H}\right|^2$ is fully written to be

$$\begin{aligned} w &= \frac{1}{4}\frac{1}{1+|q|^2}\left[\left|\left(f_r^* e^{-im\theta}, f_\theta^* e^{-im\theta}, q^* f_z^* e^{im\theta}\right)\right|^2+\left|\left(qh_r e^{-im\theta}, qh_\theta e^{-im\theta}, h_z e^{im\theta}\right)\right|^2\right] \\ &= \frac{1}{4}\frac{1}{1+|q|^2}\left(|f_r|^2+|f_\theta|^2+|q|^2|f_z|^2+|q|^2|h_r|^2+|q|^2|h_\theta|^2+|h_z|^2\right) \end{aligned}. \tag{S2.1}$$

By either Eq. (S1.3) or Eq. (S1.5), Eq. (S2.1) is cast into the following formula in terms of (f_z, h_z).

$$w=\frac{1}{4}\frac{1}{1+|q|^2}\left(\frac{m^2}{\bar{\rho}^2}|h_z|^2+\left|\frac{dh_z}{d\bar{\rho}}\right|^2+|q|^2|f_z|^2+|q|^2\frac{m^2}{\bar{\rho}^2}|f_z|^2+|q|^2\left|\frac{df_z}{d\bar{\rho}}\right|^2+|h_z|^2\right) \tag{S2.2}$$

The final step is to call upon the pair of solutions $f_z(\bar{\rho})=F_m^{\pm}(\bar{\rho})$ and $h_z(\bar{\rho})=NF_m^{\pm}(\bar{\rho})$ to find

$$w = \frac{1}{4}\frac{N^2+|q|^2}{1+|q|^2}\left(1+\left|G_m^\pm\right|^2+\frac{m^2}{\bar{\rho}^2}\right)\left|F_m^\pm\right|^2 . \tag{S2.3}$$

From another viewpoint, w is separable into w_{TE} and w_{TM} for the TE and TM modes.

$$w = \frac{|q|^2}{1+|q|^2}w_{TE} + \frac{1}{1+|q|^2}w_{TM} . \tag{S2.4}$$

We then obtain $w_{TE}^\pm = \frac{1}{4}\left(1+\left|G_m^\pm\right|^2+m^2\bar{\rho}^{-2}\right)\left|F_m^\pm\right|^2$ and $w_{TM}^+ = N^2 w_{TE}^+$. It is obvious that w remains the same for both rotating cases.

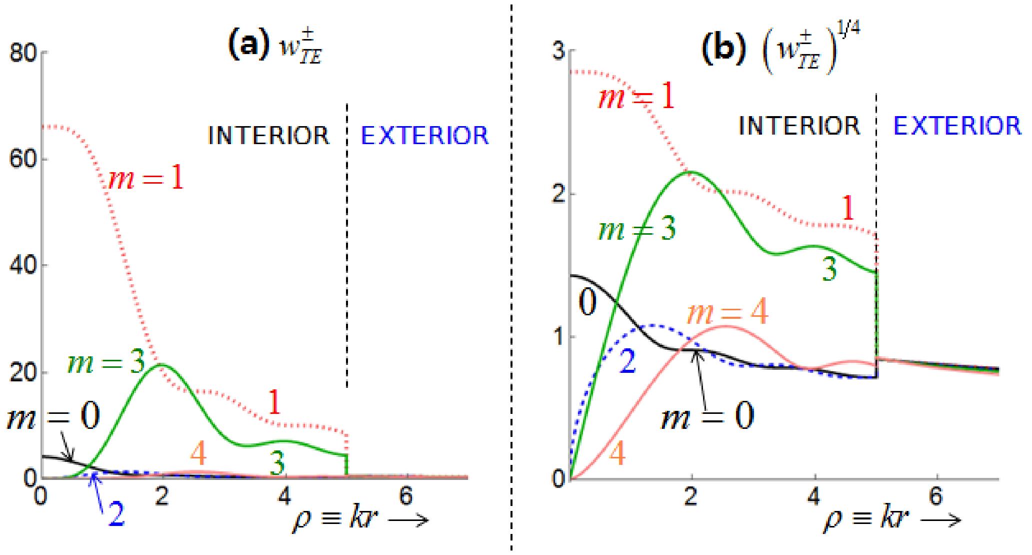

Figure S2: (a) Energy density $w_{TE}^\pm$ for the TE waves. (b) The scaled energy density $\left(w_{TE}^\pm\right)^{1/4}$ for the TE waves. Both are plotted against $\rho \equiv kr \equiv \omega r/c$ with the data $R_\rho \equiv kR = 5$. Curves are drawn with varying azimuthal index $m = 0,1,2,3,4$.

Figure S2(a) displays the nergy density $w_{TE}^\pm$ for the TE waves plotted against $\rho \equiv kr \equiv \omega r/c$. The data is $R_\rho \equiv kR = 5$. Curves are drawn with varying azimuthal index $m = 0,1,2,3,4$. It turns out that the different curves are difficult to resolve among them.

Therefore, Fig. S2(b) shows the scaled energy density $\left(w_{TE}^{\pm}\right)^{1/4}$ instead of just $w_{TE}^{\pm}$. We find that on the $\left(w_{TE}^{\pm}\right)^{1/4}$-scale, the effects of varying m are easily discernible.

S3. Optical Chirality

The optical chirality $C \equiv -\left(2n\right)^{-1}\mathrm{Im}\left(\vec{E}^{*}\cdot\vec{H}\right)$ is fully expressed as follows for the co-rotational case.

$$C = -\frac{1}{2}\frac{1}{1+|q|^2}\mathrm{Im}\left[\left(f_r^{*}e^{-im\theta}, f_\theta^{*}e^{-im\theta}, q^{*}f_z^{*}e^{-im\theta}\right)\bullet\left(qh_r e^{im\theta}, qh_\theta e^{im\theta}, h_z e^{im\theta}\right)\right]. \tag{S3.1}$$

Upon scalar multiplication, the exponential factors $e^{\pm im\theta}$ are cancelled so that

$$\begin{aligned} C &= -\frac{1}{2}\frac{1}{1+|q|^2}\mathrm{Im}\left[\left(qf_r^{*}h_r + qf_\theta^{*}h_\theta + q^{*}f_z^{*}h_z\right)\right] \\ &= \frac{1}{2}\frac{1}{1+|q|^2}\mathrm{Im}\left[q\left(-f_r^{*}h_r - f_\theta^{*}h_\theta + h_z^{*}f_z\right)\right] \end{aligned}. \tag{S3.2}$$

By Eq. (S1.3), Eq. (S3.2) is cast into the following formula only in terms of $\left(f_z, h_z\right)$.

$$\begin{aligned} C &= \frac{1}{2}\frac{1}{1+|q|^2}\mathrm{Im}\left[q\left(\frac{m}{\bar{\rho}}h_z^{*}\frac{m}{\bar{\rho}}f_z - i\frac{dh_z^{*}}{d\bar{\rho}}i\frac{df_z}{d\bar{\rho}} + h_z^{*}f_z\right)\right] \\ &= \frac{1}{2}\frac{1}{1+|q|^2}\mathrm{Im}\left[q\left(\frac{m^2}{\bar{\rho}^2}h_z^{*}f_z + \frac{dh_z^{*}}{d\bar{\rho}}\frac{df_z}{d\bar{\rho}} + h_z^{*}f_z\right)\right] \end{aligned}. \tag{S3.3}$$

Recalling that $\sigma \equiv 2\left(1+|q|^2\right)^{-1}\mathrm{Im}\left(q\right)$, the final step is to call upon the pair of solutions $f_z\left(\bar{\rho}\right) = F_m^{\pm}\left(\bar{\rho}\right)$ and $h_z\left(\bar{\rho}\right) = NF_m^{\pm}\left(\bar{\rho}\right)$ to arrive at

$$C = \frac{1}{2}\frac{N}{1+|q|^2}\mathrm{Im}\left(q\right)\left|F_m^{\pm}\right|^2\left(1+\left|G_m^{\pm}\right|^2 + \frac{m^2}{\bar{\rho}^2}\right), \tag{S3.4}$$

$$C = \tfrac{1}{4}\sigma C_m^{\pm}, \qquad C_m^{\pm} = N\left|F_m^{\pm}\right|^2\left(1+\left|G_m^{\pm}\right|^2+\frac{m^2}{\bar{\rho}^2}\right). \tag{S3.5}$$

As expected, the chirality coefficient is positive for all $\bar{\rho}$, namely, $C_m^{\pm} > 0$. In addition, C does not explicitly depend on θ. Most importantly, $C_m^{\pm}(\bar{\rho})$ is proportional to the energy density $w(\bar{\rho})$ in Eq. (S2.3) in its radial dependence.

Figure S3.1 presents contour plots of the unscaled C in (a), and the scaled $\operatorname{sgn}(C)|C|^{1/5}$ on the (kx, ky)-plane for the co-rotational case. The data are $R_\rho \equiv kR = 1$ and $q = i$. We clearly observe only a radial inhomogeneity. The white region in panel (a) signifies higher values of optical chirality. Therefore, the scaled one in panel (b) offers a better view most of the time.

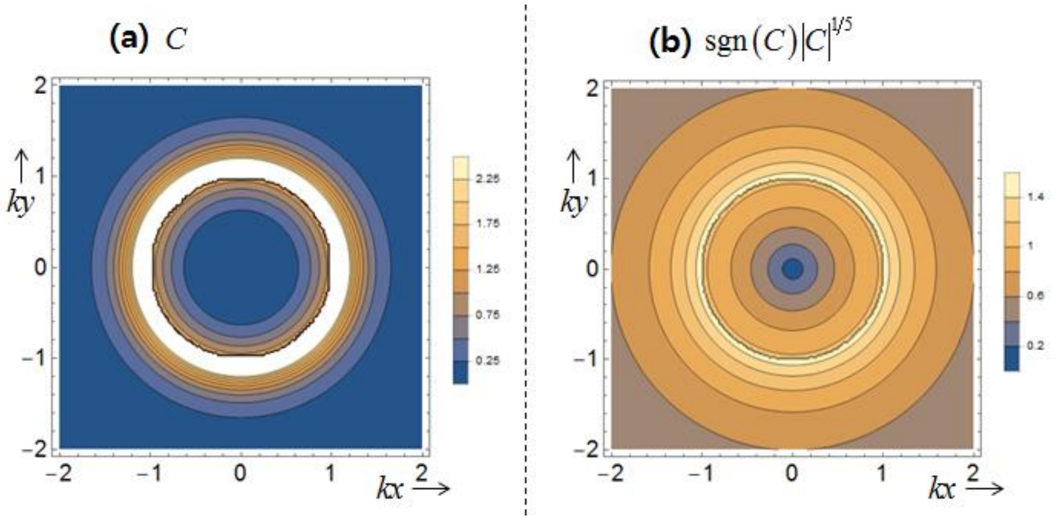

Figure S3.1: (a) A contour plot of the optical chirality C. (b) A contour plot of the scaled optical chirality $\operatorname{sgn}(C)|C|^{1/5}$. Both are plotted on the (kx, ky)-plane for the co-rotational case. The data are $R_\rho \equiv kR = 1$, $m = 3$, and $q = i$.

For the counter-rotational case, the optical chirality $C \equiv -(2n)^{-1}\operatorname{Im}\left(\vec{E}^*\cdot\vec{H}\right)$ is fully expressed as follows.

$$C = -\frac{1}{2}\frac{1}{1+|q|^2}\,\mathrm{Im}\left[\left(f_r^* e^{-im\theta}, f_\theta^* e^{-im\theta}, q^* f_z^* e^{im\theta}\right)\bullet\left(q h_r e^{-im\theta}, q h_\theta e^{-im\theta}, h_z e^{im\theta}\right)\right]. \tag{S3.6}$$

Even upon simplification, the azimuthal angle survives explicitly such that

$$\begin{aligned} C &= -\frac{1}{2}\frac{1}{1+|q|^2}\,\mathrm{Im}\left[\left(q e^{-2im\theta} f_r^* h_r + q e^{-2im\theta} f_\theta^* h_\theta + q^* e^{2im\theta} f_z^* h_z\right)\right] \\ &= \frac{1}{2}\frac{1}{1+|q|^2}\,\mathrm{Im}\left[q e^{-2im\theta}\left(-f_r^* h_r - f_\theta^* h_\theta + h_z^* f_z\right)\right] \end{aligned}. \tag{S3.7}$$

By Eq. (S1.5), Eq. (S3.7) is cast into the following formula in terms of $\left(f_z, h_z\right)$.

$$\begin{aligned} C &= \frac{1}{2}\frac{1}{1+|q|^2}\,\mathrm{Im}\left[q e^{-2im\theta}\left(-\frac{-m}{\bar\rho} h_z^* \frac{-m}{\bar\rho} f_z - i\frac{dh_z^*}{d\bar\rho} i\frac{df_z}{d\bar\rho} + h_z^* f_z\right)\right] \\ &= \frac{1}{2}\frac{1}{1+|q|^2}\,\mathrm{Im}\left[q e^{-2im\theta}\left(-\frac{m^2}{\bar\rho^2} f_z^* h_z + \frac{dh_z^*}{d\bar\rho}\frac{df_z}{d\bar\rho} + f_z^* h_z\right)\right] \end{aligned}. \tag{S3.8}$$

The final step is to call upon the pair of solutions $f_z\left(\bar\rho\right) = F_m^\pm\left(\bar\rho\right)$ and $h_z\left(\bar\rho\right) = N F_m^\pm\left(\bar\rho\right)$ to arrive at

$$C = \tfrac{1}{4}\chi C_m^\pm,\ \ C_m^\pm \equiv N\left|F_m^\pm\right|^2\left(1+\left|G_m^\pm\right|^2 - \frac{m^2}{\bar\rho^2}\right). \tag{S3.9}$$

Here, χ is the chirality defined in Eq. (S1.11). As expected, the azimuthal term $-m^2/\bar\rho^2$ in $C_m^\pm$ for this counter-rotational case changes its sign, in comparison to $+m^2/\bar\rho^2$ in Eq. (S3.5) for the co-rotational case.

Figure S3.2 shows a contour plot of $C\left(\bar\rho,\theta\right)$ translated into $C\left(kx,ky\right)$ on the $\left(kx,ky\right)$-plane for $m=1$ with the data $R_\rho \equiv kR = 1$ and $q = i$. Here, the pattern repeats twice because of the factor $\cos\left(2im\theta\right)$ with $m=1$.

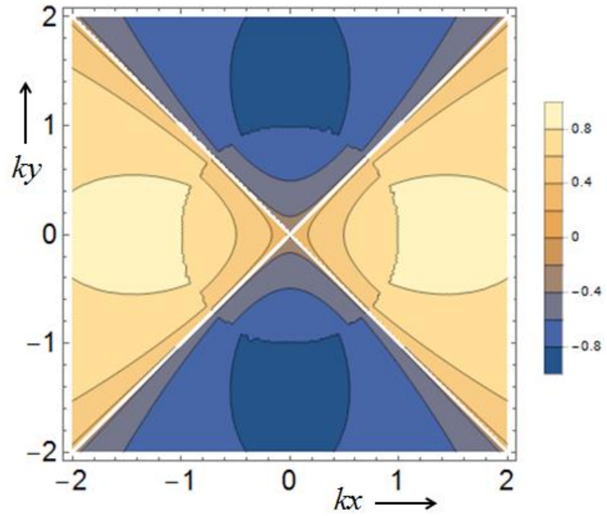

Figure S3.2: A contour plot for the scaled optical chirality $\mathrm{sgn}(C)|C|^{0.2}$ on the (kx, ky)-plane for the counter-rotational case. The data are $R_\rho \equiv kR = 1$, $m = 1$, and $q = i$.

S4. Poynting Vector

For the co-rotational case, the Poynting vector $\vec{P}^{Poyn} \equiv (2n)^{-1}\mathrm{Re}\left(\vec{E}^* \times \vec{H}\right)$ is fully written to be

$$\vec{P}^{Poyn} = \frac{n^{\pm}}{2}\frac{1}{1+|q|^2}\mathrm{Re}\left[\left(f_r^* e^{-im\theta}, f_\theta^* e^{-im\theta}, q^* f_z^* e^{-im\theta}\right) \times \left(q h_r e^{im\theta}, q h_\theta e^{im\theta}, h_z e^{im\theta}\right)\right]. \quad \text{(S4.1)}$$

As a result, we obtain the component-wise relations for $\vec{P}^{Poyn} = P_r^{Poyn}\hat{e}_r + P_\theta^{Poyn}\hat{e}_\theta + P_z^{Poyn}\hat{e}_z$ as follows.

$$\begin{cases} P_r^{Poyn} = \dfrac{n^{\pm}}{2}\dfrac{1}{1+|q|^2}\mathrm{Re}\left(f_\theta^* h_z - |q|^2 f_z^* h_\theta\right) \\ P_\theta^{Poyn} = \dfrac{n^{\pm}}{2}\dfrac{1}{1+|q|^2}\mathrm{Re}\left(|q|^2 f_z^* h_r - f_r^* h_z\right) \\ P_z^{Poyn} = \dfrac{n^{\pm}}{2}\dfrac{1}{1+|q|^2}\mathrm{Re}\left[q\left(f_r^* h_\theta - f_\theta^* h_r\right)\right] \end{cases} . \quad \text{(S4.2)}$$

Note that $e^{im\theta}$ cancels out $e^{-im\theta}$ in every terms. By Eq. (S1.3), these three are cast into the following relations in terms of $\left(f_z, h_z\right)$.

$$\begin{cases} P_r^{Poyn} = \dfrac{n^\pm}{2}\dfrac{1}{1+|q|^2}\mathrm{Re}\left(i\dfrac{dh_z^*}{d\bar{\rho}}h_z - i|q|^2 f_z^* \dfrac{df_z}{d\bar{\rho}}\right) \\ P_\theta^{Poyn} = \dfrac{n^\pm}{2}\dfrac{1}{1+|q|^2}\mathrm{Re}\left(|q|^2 f_z^* \dfrac{m}{\bar{\rho}} f_z + \dfrac{m}{\bar{\rho}} h_z^* h_z\right) \\ P_z^{Poyn} = \dfrac{n^\pm}{2}\dfrac{1}{1+|q|^2}\mathrm{Re}\left[q\left(-\dfrac{m}{\bar{\rho}}h_z^* i\dfrac{df_z}{d\bar{\rho}} - i\dfrac{dh_z^*}{d\bar{\rho}}\dfrac{m}{\bar{\rho}} f_z\right)\right] \end{cases}. \tag{S4.3}$$

With the help of various relations for complex variables, these three are further simplified.

$$\begin{cases} P_r^{Poyn} = \dfrac{n^\pm}{2}\dfrac{1}{1+|q|^2}\mathrm{Im}\left(|q|^2 f_z^* \dfrac{df_z}{d\bar{\rho}} - \dfrac{dh_z^*}{d\bar{\rho}}h_z\right) \\ P_\theta^{Poyn} = \dfrac{n^\pm}{2}\dfrac{|h_z|^2+|q|^2|f_z|^2}{1+|q|^2}\dfrac{m}{\bar{\rho}} \\ P_z^{Poyn} = \dfrac{n^\pm}{2}\dfrac{1}{1+|q|^2}\dfrac{m}{\bar{\rho}}\mathrm{Im}\left[q\left(h_z^* \dfrac{df_z}{d\bar{\rho}} + \dfrac{dh_z^*}{d\bar{\rho}} f_z\right)\right] \end{cases}. \tag{S4.4}$$

The final step is to call upon the pair of solutions $f_z\left(\bar{\rho}\right) = F_m^\pm\left(\bar{\rho}\right)$ and $h_z\left(\bar{\rho}\right) = NF_m^\pm\left(\bar{\rho}\right)$ to find

$$\begin{cases} P_r^{Poyn} = \dfrac{n^\pm}{2}\dfrac{1}{1+|q|^2}\mathrm{Im}\left(|q|^2 F_m^{\pm*}\dfrac{dF_m^\pm}{d\bar{\rho}} - N^2\dfrac{dF_m^{\pm*}}{d\bar{\rho}}F_m^\pm\right) \\ P_\theta^{Poyn} = \dfrac{n^\pm}{2}\dfrac{N^2+|q|^2}{1+|q|^2}\dfrac{m}{\bar{\rho}}\left|F_m^\pm\right|^2 \\ P_z^{Poyn} = \dfrac{n^\pm}{2}\dfrac{1}{1+|q|^2}\dfrac{m}{\bar{\rho}}\mathrm{Im}\left[qN\left(F_m^{\pm*}\dfrac{dF_m^\pm}{d\bar{\rho}} + \dfrac{dF_m^{\pm*}}{d\bar{\rho}}F_m^\pm\right)\right]. \end{cases}. \tag{S4.5}$$

By way of the helicity $\sigma \equiv \left(1+|q|^2\right)^{-1} 2\,\mathrm{Im}\left(q\right)$, two of Eq. (S4.5) get further down to

$$\begin{cases} P_r^{Poyn} = \dfrac{n^{\pm}}{2} \dfrac{|q|^2 + N^2}{1+|q|^2} \left|F_m^{\pm}\right|^2 \mathrm{Im}\left(G_m^{\pm}\right) \\ P_z^{Poyn} = \sigma \dfrac{n^{\pm}}{2} N \dfrac{m}{\bar{\rho}} \left|F_m^{\pm}\right|^2 \mathrm{Re}\left(G_m^{\pm}\right) \end{cases}. \tag{S4.6}$$

The trajectories for the Poynting-vector flows are evaluated from the following differential forms.

$$\frac{\bar{\rho} d\theta}{d\bar{\rho}} = \frac{P_\theta^{Poyn}}{P_r^{Poyn}}, \quad \frac{d(kz)}{d\bar{\rho}} = \frac{1}{n^{\pm}} \frac{P_z^{Poyn}}{P_r^{Poyn}}. \tag{S4.7}$$

We then rewrite these two relations into the following differential trajectories with $\bar{\rho}$ as a parameter, based on Eqs. (S4.5) and (S4.6).

$$\begin{cases} \dfrac{d\theta}{d\bar{\rho}} = \dfrac{m}{\bar{\rho}^2 \mathrm{Im}\left(G_m^{\pm}\right)} \\ \dfrac{d(kz)}{d\bar{\rho}} = \dfrac{2}{n^{\pm}} \dfrac{N}{|q|^2 + N^2} \dfrac{m}{\bar{\rho}} \dfrac{\mathrm{Re}\left(G_m^{\pm}\right)\mathrm{Im}(q)}{\mathrm{Im}\left(G_m^{\pm}\right)} \\ \dfrac{d(kz)}{d\theta} = \dfrac{2}{n^{\pm}} \dfrac{N}{|q|^2 + N^2} \bar{\rho}\, \mathrm{Re}\left(G_m^{\pm}\right)\mathrm{Im}(q) \end{cases}. \tag{S4.8}$$

For the counter-rotational case, the Poynting vector $\vec{P}^{Poyn} \equiv (2n)^{-1} \mathrm{Re}\left(\vec{E}^* \times \vec{H}\right)$ is fully written to be

$$\vec{P}^{Poyn} = \frac{n^{\pm}}{2} \frac{1}{1+|q|^2} \mathrm{Re}\left[\left(f_r^* e^{-im\theta}, f_\theta^* e^{-im\theta}, q^* f_z^* e^{im\theta}\right) \times \left(q h_r e^{-im\theta}, q h_\theta e^{-im\theta}, h_z e^{im\theta}\right)\right]. \tag{S4.9}$$

As a result, we obtain the component-wise relations for $\vec{P}^{Poyn} = P_r^{Poyn}\hat{e}_r + P_\theta^{Poyn}\hat{e}_\theta + P_z^{Poyn}\hat{e}_z$ as in (S4.2). Note that $e^{im\theta}$ cancels out $e^{-im\theta}$ in every terms. By Eq. (S1.5), these three are cast into the following relations in terms of (f_z, h_z).

$$\begin{cases} P_r^{Poyn} = \dfrac{n^{\pm}}{2}\dfrac{1}{1+|q|^2}\mathrm{Re}\left(i\dfrac{dh_z^*}{d\bar{\rho}}h_z - i|q|^2 f_z^* \dfrac{df_z}{d\bar{\rho}} \right) \\ P_\theta^{Poyn} = \dfrac{n^{\pm}}{2}\dfrac{1}{1+|q|^2}\mathrm{Re}\left(-|q|^2 f_z^* \dfrac{m}{\bar{\rho}} f_z + \dfrac{m}{\bar{\rho}} h_z^* h_z \right) \\ P_z^{Poyn} = \dfrac{n^{\pm}}{2}\dfrac{1}{1+|q|^2}\mathrm{Re}\left[q\left(-\dfrac{m}{\bar{\rho}} h_z^* i \dfrac{df_z}{d\bar{\rho}} - i\dfrac{dh_z^*}{d\bar{\rho}} \dfrac{-m}{\bar{\rho}} f_z \right)\right] \end{cases} . \tag{S4.10}$$

Notice the sign change in the expression for P_θ^{Poyn} and P_z^{Poyn} (in red colors) in the above equation. With the help of various relations for complex variables, these three are further simplified.

$$\begin{cases} P_r^{Poyn} = \dfrac{n^{\pm}}{2}\dfrac{1}{1+|q|^2}\mathrm{Im}\left(|q|^2 f_z^* \dfrac{df_z}{d\bar{\rho}} - \dfrac{dh_z^*}{d\bar{\rho}} h_z \right) \\ P_\theta^{Poyn} = \dfrac{n^{\pm}}{2}\dfrac{|h_z|^2 - |q|^2 |f_z|^2}{1+|q|^2}\dfrac{m}{\bar{\rho}} \\ P_z^{Poyn} = \dfrac{n^{\pm}}{2}\dfrac{1}{1+|q|^2}\dfrac{m}{\bar{\rho}}\mathrm{Im}\left[q\left(h_z^* \dfrac{df_z}{d\bar{\rho}} - \dfrac{dh_z^*}{d\bar{\rho}} f_z \right)\right] \end{cases} . \tag{S4.11}$$

The final step is to call upon the pair of solutions $f_z(\bar{\rho}) = F_m^{\pm}(\bar{\rho})$ and $h_z(\bar{\rho}) = NF_m^{\pm}(\bar{\rho})$ to find

$$\begin{cases} P_r^{Poyn} = \dfrac{n^{\pm}}{2}\dfrac{1}{1+|q|^2}\mathrm{Im}\left(|q|^2 F_m^{\pm *} \dfrac{dF_m^{\pm}}{d\bar{\rho}} - N^2 \dfrac{dF_m^{\pm *}}{d\bar{\rho}} F_m^{\pm} \right) \\ P_\theta^{Poyn} = \dfrac{n^{\pm}}{2}\dfrac{N^2 - |q|^2}{1+|q|^2}\dfrac{m}{\bar{\rho}}\left|F_m^{\pm}\right|^2 \\ P_z^{Poyn} = \dfrac{n^{\pm}}{2}\dfrac{1}{1+|q|^2}\dfrac{m}{\bar{\rho}}\mathrm{Im}\left[qN\left(F_m^{\pm *} \dfrac{dF_m^{\pm}}{d\bar{\rho}} - \dfrac{dF_m^{\pm *}}{d\bar{\rho}} F_m^{\pm} \right)\right]. \end{cases} . \tag{S4.12}$$

By way of the helicity $\sigma \equiv \left(1+|q|^2\right)^{-1} 2\,\mathrm{Im}(q)$, two of Eq. (S4.12) get further down to

$$\begin{cases} P_r^{Poyn} = \frac{n^{\pm}}{2}\frac{|q|^2+N^2}{1+|q|^2}\left|F_m^{\pm}\right|^2 \operatorname{Im}\left(G_m^{\pm}\right) \\ P_z^{Poyn} = \sigma\frac{n^{\pm}}{2}N\frac{m}{\bar{\rho}}\left|F_m^{\pm}\right|^2 \operatorname{Im}\left(G_m^{\pm}\right) \end{cases}. \tag{S4.13}$$

It should be noticed that $P_z^{Poyn} \propto \operatorname{Re}\left(G_m^{\pm}\right)$ in Eq. (S4.6) and $P_z^{Poyn} \propto \operatorname{Im}\left(G_m^{\pm}\right)$ in Eq. (S4.13) respectively for the co- and counter-rotational cases.

The trajectories for the Poynting-vector flows are evaluated from Eq. (S4.7). We then rewrite these two relations into the following differential trajectories with $\bar{\rho}$ as a parameter, based on Eqs. (S4.12) and (S4.13).

$$\begin{cases} \frac{d\theta}{d\bar{\rho}} = \frac{N^2-|q|^2}{N^2+|q|^2}\frac{m}{\bar{\rho}^2 \operatorname{Im}\left(G_m^{\pm}\right)} \\ \frac{d(kz)}{d\bar{\rho}} = \frac{2}{n^{\pm}}\frac{N}{|q|^2+N^2}\frac{m}{\bar{\rho}}\operatorname{Im}(q) \\ \frac{d(kz)}{d\theta} = \frac{2}{n^{\pm}}\frac{N}{N^2-|q|^2}\bar{\rho}\operatorname{Im}\left(G_m^{\pm}\right)\operatorname{Im}(q) \end{cases}. \tag{S4.14}$$

S5. Energy Flow Density and Poynting Vector

Via $k \equiv \omega/c$ and the Maxwell's equations $-i\varepsilon\vec{E} = \nabla_k \times \vec{H}$ and $i\mu\vec{H} = \nabla_k \times \vec{E}$ with $\nabla_k \equiv k^{-1}\nabla$, we have the total energy flow density (FD) $\vec{P}^{tot}$ as follows.

$$\begin{aligned} \vec{P}^{tot} &\equiv \frac{1}{4n}\operatorname{Im}\left[\frac{1}{\mu}\vec{E}^*\times\left(\nabla_k\times\vec{E}\right)+\frac{1}{\varepsilon}\vec{H}^*\times\left(\nabla_k\times\vec{H}\right)\right] \\ &= \frac{1}{4n}\operatorname{Im}\left[\frac{1}{\mu}\vec{E}^*\times\left(i\mu\vec{H}\right)+\frac{1}{\varepsilon}\vec{H}^*\times\left(-i\varepsilon\vec{E}\right)\right] \\ &= \frac{1}{4n}\operatorname{Im}\left(i\vec{E}^*\times\vec{H}-i\vec{H}^*\times\vec{E}\right) = \frac{1}{4n}\operatorname{Im}\left(i\vec{E}^*\times\vec{H}+i\vec{E}\times\vec{H}^*\right) \\ &= \frac{1}{4n}\operatorname{Re}\left(\vec{E}^*\times\vec{H}+\vec{E}\times\vec{H}^*\right) = \frac{1}{2n}\operatorname{Re}\left(\vec{E}^*\times\vec{H}\right) \equiv \vec{P}^{Poyn} \end{aligned}. \tag{S5.1}$$

As a result, the total energy flow density (FD) is identical to the Poynting vector (PV), namely, $\vec{P}^{tot} = \vec{P}^{Poyn}$ for dielectric media. This fact holds true to both rotational cases.

Meanwhile, the decomposition of $\vec{P}^{tot}$ into its orbital FD $\vec{P}^{O}$ and spin FD $\vec{P}^{S}$ is easier to handle in the Cartesian coordinates by use of the repeated indices.

$$nk\vec{P}^{O} \equiv \frac{1}{\mu}\mathrm{Im}\left[\vec{E}^{*}\cdot(\nabla)\vec{E}\right] + \frac{1}{\varepsilon}\mathrm{Im}\left[\vec{H}^{*}\cdot(\nabla)\vec{H}\right], \tag{S5.2}$$

$$nk\vec{P}^{S} \equiv \frac{1}{2\mu}\nabla\times\mathrm{Im}\left(\vec{E}^{*}\times\vec{E}\right) + \frac{1}{2\varepsilon}\nabla\times\mathrm{Im}\left(\vec{H}^{*}\times\vec{H}\right). \tag{S5.3}$$

In order prove the identity $\vec{P}^{tot} = \vec{P}_0^{O} + \vec{P}_0^{S}$, let us prove the following identity for the electric field first.

$$\mathrm{Im}\left[\vec{E}^{*}\times\left(\nabla\times\vec{E}\right)\right] = \mathrm{Im}\left[\vec{E}^{*}\cdot(\nabla)\vec{E}\right] + \tfrac{1}{2}\nabla\times\mathrm{Im}\left(\vec{E}^{*}\times\vec{E}\right). \tag{S5.4}$$

To prove Eq. (S5.4), consider $\tfrac{1}{2}\nabla\times\left(\vec{E}^{*}\times\vec{E}\right)$ first.

$$\begin{aligned}
&\tfrac{1}{2}\nabla\times\left(\vec{E}^{*}\times\vec{E}\right) = \tfrac{1}{2}\varepsilon_{ljk}\frac{\partial}{\partial x_j}\left(\vec{E}^{*}\times\vec{E}\right)_k \hat{e}_l \\
&= \tfrac{1}{2}\varepsilon_{ljk}\frac{\partial}{\partial x_j}\left(\varepsilon_{kmn}E_m^{*}E_n\right)\hat{e}_l = \tfrac{1}{2}\varepsilon_{kmn}\varepsilon_{klj}\frac{\partial\left(E_m^{*}E_n\right)}{\partial x_j}\hat{e}_l \\
&= \tfrac{1}{2}\frac{\partial}{\partial x_j}\left[\left(\delta_{ml}\delta_{nj} - \delta_{mj}\delta_{nl}\right)E_m^{*}E_n\right]\hat{e}_l = \tfrac{1}{2}\frac{\partial}{\partial x_j}\left(E_l^{*}E_j - E_j^{*}E_l\right)\hat{e}_l
\end{aligned} \; . \tag{S5.5}$$

Note that δ_{ij} and ε_{ljk} are the Kronecker and Levi-Civita symbols, respectively. Besides, $\hat{e}_i$ is the unit basis vector of the Cartesian coordinates. We made use of the identity $\varepsilon_{kmn}\varepsilon_{klj} = \delta_{ml}\delta_{nj} - \delta_{mj}\delta_{nl}$. Hence, taking the imaginary parts on both sides of Eq. (S5.5) leads to

$$\tfrac{1}{2}\nabla\times\mathrm{Im}\left(\vec{E}^{*}\times\vec{E}\right)=\tfrac{1}{2}\frac{\partial}{\partial x_j}\mathrm{Im}\left(E_l^{*}E_j-E_j^{*}E_l\right)\hat{e}_l$$

$$=\frac{\partial}{\partial x_j}\mathrm{Im}\left(E_l^{*}E_j\right)\hat{e}_l=\mathrm{Im}\left(\frac{\partial E_l^{*}}{\partial x_j}E_j\hat{e}_l\right)+\mathrm{Im}\left(E_l^{*}\frac{\partial E_j}{\partial x_j}\hat{e}_l\right). \qquad \text{(S5.6)}$$

For dielectric media without space charges, we have $\partial E_j/\partial x_j\equiv\nabla\cdot\vec{E}=0$. As a result, Eq. (S5.6) is reduced to

$$\tfrac{1}{2}\nabla\times\mathrm{Im}\left(\vec{E}^{*}\times\vec{E}\right)+\mathrm{Im}\left(E_j^{*}\frac{\partial E_l}{\partial x_j}\hat{e}_l\right)=0. \qquad \text{(S5.7)}$$

On the other hand, consider next $\vec{E}^{*}\times\left(\nabla\times\vec{E}\right)$.

$$\vec{E}^{*}\times\left(\nabla\times\vec{E}\right)=\vec{E}_j^{*}\left(\nabla\times\vec{E}\right)_k\varepsilon_{ljk}\hat{e}_l=\vec{E}_j^{*}\left(\varepsilon_{kmn}\frac{\partial E_n}{\partial x_m}\right)\varepsilon_{ljk}\hat{e}_l$$

$$=\varepsilon_{kmn}\varepsilon_{klj}\vec{E}_j^{*}\frac{\partial E_n}{\partial x_m}\hat{e}_l=\left(\delta_{ml}\delta_{nj}-\delta_{mj}\delta_{nl}\right)\vec{E}_j^{*}\frac{\partial E_n}{\partial x_m}\hat{e}_l=\vec{E}_j^{*}\frac{\partial E_j}{\partial x_l}\hat{e}_l-\vec{E}_j^{*}\frac{\partial E_l}{\partial x_j}\hat{e}_l. \qquad \text{(S5.8)}$$

Because of the defining relation $\vec{E}^{*}\cdot\left(\nabla\right)\vec{E}\equiv E_j^{*}\left(\partial E_j/\partial x_i\right)\hat{e}_i$, the combination of Eqs. (S5.7) and (S5.8) ushers us to the desired identity in Eq. (S5.4).

In similarity to Eq. (5.4), we could prove its magnetic counterpart.

$$\mathrm{Im}\left[\vec{H}^{*}\times\left(\nabla\times\vec{H}\right)\right]=\mathrm{Im}\left[\vec{H}^{*}\cdot\left(\nabla\right)\vec{H}\right]+\tfrac{1}{2}\nabla\times\mathrm{Im}\left(\vec{H}^{*}\times\vec{H}\right). \qquad \text{(S5.9)}$$

As a consequence of Eqs. (S5.2), (S5.3), (S5.4), (S5.9), we have proved that $\vec{P}^{tot}=\vec{P}_0^{O}+\vec{P}_0^{S}$. It is worth emphasizing that the identity $\vec{P}^{tot}=\vec{P}_0^{O}+\vec{P}_0^{S}$ has been proved not by the identity $\vec{E}^{*}\times\left(\nabla\times\vec{E}\right)=\vec{E}^{*}\cdot\left(\nabla\right)\vec{E}+\tfrac{1}{2}\nabla\times\left(\vec{E}^{*}\times\vec{E}\right)$ for complex fields, but by its respective imaginary parts. In addition, we should remark that the divergence-free conditions $\nabla\cdot\vec{E}=0$ and

$\nabla\cdot\vec{H}=0$ have been incorporated in the procedure. In other words, homogeneous dielectric media are assumed without any presence of space charges.

S6. Orbital Flow Density in the Cartesian Coordinates

Now, let us further process the orbital part $\vec{P}^O$ for our particular waves described by $f_z(\bar{\rho})=F_m^{\pm}(\bar{\rho})$ and $h_z(\bar{\rho})=NF_m^{\pm}(\bar{\rho})$. We rely on the Maxwell's equations $-i\varepsilon\vec{E}=\nabla_k\times\vec{H}$ and $i\mu\vec{H}=\nabla_k\times\vec{E}$ with $\nabla_k\equiv k^{-1}\nabla$, expressed in the frequency domain via the proportionality $\exp(-i\omega t)$. For dimensional reasons, let us introduce $(\bar{x},\bar{y},\bar{z})\equiv nk(x,y,z)$. By this way, we obtain $(\bar{x},\bar{y})\equiv\bar{\rho}(\cos\theta,\sin\theta)$ based on the previously introduced radial coordinate $\bar{\rho}\equiv n^{\pm}kr$.

For axial-coordinate-independent field variables, we reduce $-i\varepsilon\vec{E}=\nabla_k\times\vec{H}$ and $i\mu\vec{H}=\nabla_k\times\vec{E}$ to the following.

$$\begin{cases} TE: \ \ h_x=-i\dfrac{\partial f_z}{\partial\bar{y}}, \ \ h_y=i\dfrac{\partial f_z}{\partial\bar{x}} \\ TM: \ \ f_x=i\dfrac{\partial h_z}{\partial\bar{y}}, \ \ f_y=-i\dfrac{\partial h_z}{\partial\bar{x}} \end{cases}. \tag{S6.1}$$

Consider the electric field first by noticing that $\vec{E}^*\cdot(\nabla)\vec{E}\equiv E_j^*\left(\partial E_j/\partial x_i\right)\hat{e}_i$. Here, one is prone to making a grave mistake when setting $\mathrm{Im}\left[E_j^*\left(\partial E_j/\partial x_i\right)\hat{e}_i\right]=\mathrm{Im}\left[\tfrac{1}{2}\left(\partial/\partial x_i\right)\left(E_j^*E_j\right)\hat{e}_i\right]=\mathrm{Im}\left[\left(\partial/\partial x_i\right)\left(\tfrac{1}{2}\left|\vec{E}\right|^2\right)\hat{e}_i\right]=0$, which is absolutely incorrect!. In other words, although $\left|\vec{E}\right|^2\equiv E_j^*E_j$, we find that

$$E_j^*\frac{\partial E_j}{\partial x_i}\hat{e}_i\neq\tfrac{1}{2}\frac{\partial\left(E_j^*E_j\right)}{\partial x_i}\hat{e}_i. \tag{S6.2}$$

Nonetheless, there is a more informative interpretation for $\vec{E}^* \cdot (\nabla)\vec{E}$ if we write the electric field as $E_j \equiv |E_j| \exp(i\psi_j)$ with ψ_j as its real-valued phase. As a result,

$$\begin{aligned}
\vec{E}^* \cdot (\nabla)\vec{E} &\equiv E_j^* \frac{\partial E_j}{\partial x_i}\hat{e}_i = \sum_j |E_j| \exp(-i\psi_j) \frac{\partial\left[|E_j|\exp(i\psi_j)\right]}{\partial x_i}\hat{e}_i \\
&= \sum_j |E_j| \exp(-i\psi_j)\exp(i\psi_j)\frac{\partial|E_j|}{\partial x_i}\hat{e}_i + \sum_j |E_j|^2 \exp(-i\psi_j)\frac{\partial\left[\exp(i\psi_j)\right]}{\partial x_i}\hat{e}_i \\
&= |E_j|\frac{\partial|E_j|}{\partial x_i}\hat{e}_i + i\sum_j |E_j|^2 \exp(-i\psi_j)\exp(i\psi_j)\frac{\partial\psi_j}{\partial x_i}\hat{e}_i \\
&= \tfrac{1}{2}\frac{\partial|\vec{E}|^2}{\partial x_i}\hat{e}_i + i|E_j|^2\frac{\partial\psi_j}{\partial x_i}\hat{e}_i
\end{aligned} \quad . \qquad \text{(S6.3)}$$

This expansion make a correction for the above-mentioned mistake in Eq. (S6.2). Let us explain the summation conventions employed here. Any double indices mean a summation over that index as in the Einstein's notation. For the triple (3 times) or quartic (4 times) indices, we employ the summation symbol $\sum_j$ in an explicit way. Therefore,

$$\begin{cases}
|\vec{E}|^2 \equiv E_j^* E_j \equiv \sum_j E_j^* E_j \\
|E_j|^2 \dfrac{\partial\psi_j}{\partial x_i}\hat{e}_i \equiv \sum_j\left(E_j^* E_j \dfrac{\partial\psi_j}{\partial x_i}\hat{e}_i\right) \equiv \sum_i\left[\sum_j\left(E_j^* E_j\dfrac{\partial\psi_j}{\partial x_i}\hat{e}_i\right)\right]
\end{cases} . \qquad \text{(S6.4)}$$

By this way, we have separated $\vec{E}^* \cdot (\nabla)\vec{E}$ into its real and imaginary parts. The first term is real-valued, and it is exactly what we have mentioned in the previous paragraph for warning for Eq. (S6.2). The second additional term is imaginary-valued, and it is what we should not miss out. This second term is the intensity of the electric field multiplied by the phase gradient. We cannot emphasize too strongly the importance of the phase gradients in the business of angular momentum. Besides, we could treat $\vec{H}^* \cdot (\nabla)\vec{H}$ in a similar way.

Now, we proceed with the electric-field part in terms of the normalized variables.

$$
\begin{aligned}
&\frac{1}{nk}\frac{1}{\mu}\vec{E}^{*}\cdot(\nabla)\vec{E} \equiv \frac{1}{\mu}\mathrm{Im}\left[E_j^{*}\frac{\partial E_j}{\partial x_i}\hat{e}_i\right] \\
&= \frac{1}{\mu}\mathrm{Im}\left[\left(E_x^{*}\frac{\partial E_x}{\partial x}+E_y^{*}\frac{\partial E_y}{\partial x}+E_z^{*}\frac{\partial E_z}{\partial x}\right)\hat{e}_x\right] \\
&+\frac{1}{\mu}\mathrm{Im}\left[\left(E_x^{*}\frac{\partial E_x}{\partial y}+E_y^{*}\frac{\partial E_y}{\partial y}+E_z^{*}\frac{\partial E_z}{\partial y}\right)\hat{e}_y\right] \\
&= \frac{1}{1+|q|^2}\mathrm{Im}\left[\left(f_x^{*}\frac{\partial f_x}{\partial x}+f_y^{*}\frac{\partial f_y}{\partial x}+|q|^2 f_z^{*}\frac{\partial f_z}{\partial x}\right)\hat{e}_x\right] \\
&+\frac{1}{1+|q|^2}\mathrm{Im}\left[\left(f_x^{*}\frac{\partial f_x}{\partial y}+f_y^{*}\frac{\partial f_y}{\partial y}+|q|^2 f_z^{*}\frac{\partial f_z}{\partial y}\right)\hat{e}_y\right]
\end{aligned}
\quad . \qquad \text{(S6.5)}
$$

Here, we utilized either Eq. (S1.2) or (S1.4) and the axial-coordinate independence of the electric field.

Similarly, we treat the magnetic field to obtain

$$
\begin{aligned}
&\frac{1}{nk}\frac{1}{\varepsilon}\vec{H}^{*}\cdot(\nabla)\vec{H} \equiv \frac{1}{\varepsilon}\mathrm{Im}\left[H_j^{*}\frac{\partial H_j}{\partial x_i}\hat{e}_i\right] \\
&= \frac{1}{\varepsilon}\mathrm{Im}\left[\left(H_x^{*}\frac{\partial H_x}{\partial x}+H_y^{*}\frac{\partial H_y}{\partial x}+H_z^{*}\frac{\partial H_z}{\partial x}\right)\hat{e}_x\right] \\
&+\frac{1}{\varepsilon}\mathrm{Im}\left[\left(H_x^{*}\frac{\partial H_x}{\partial y}+H_y^{*}\frac{\partial H_y}{\partial y}+H_z^{*}\frac{\partial H_z}{\partial y}\right)\hat{e}_y\right] \\
&= \frac{1}{1+|q|^2}\mathrm{Im}\left[\left(|q|^2 h_x^{*}\frac{\partial h_x}{\partial x}+|q|^2 h_y^{*}\frac{\partial h_y}{\partial x}+h_z^{*}\frac{\partial h_z}{\partial x}\right)\hat{e}_x\right] \\
&+\frac{1}{1+|q|^2}\mathrm{Im}\left[\left(|q|^2 h_x^{*}\frac{\partial h_x}{\partial y}+|q|^2 h_y^{*}\frac{\partial h_y}{\partial y}+h_z^{*}\frac{\partial h_z}{\partial y}\right)\hat{e}_y\right]
\end{aligned}
\quad . \qquad \text{(S6.6)}
$$

We expect instantly that both $\vec{E}^{*}\cdot(\nabla)\vec{E}$ in Eq. (S6.5) and $\vec{H}^{*}\cdot(\nabla)\vec{H}$ in Eq. (S6.6) do not differentiate between the co- and counter-rotational cases, because all complex variables show up in complex-conjugate pairs. Another observation is that there is no interference term, since only $|q|^2$ shows up. Hence, the orbital FD is separable into the TE and TM modes. Summing up Eqs. (S6.5) and (S6.6),

$$
\begin{aligned}
\vec{P}^{O} &\equiv \frac{1}{4nk\mu}\operatorname{Im}\left[\vec{E}^{*}\cdot(\nabla)\vec{E}\right]+\frac{1}{4nk\varepsilon}\operatorname{Im}\left[\vec{H}^{*}\cdot(\nabla)\vec{H}\right] \\
&= \frac{1}{4}\frac{1}{1+|q|^{2}}\operatorname{Im}\left[\left(f_x^{*}\frac{\partial f_x}{\partial\overline{x}}+f_y^{*}\frac{\partial f_y}{\partial\overline{x}}+|q|^{2}f_z^{*}\frac{\partial f_z}{\partial\overline{x}}\right)\hat{e}_x\right] \\
&+\frac{1}{4}\frac{1}{1+|q|^{2}}\operatorname{Im}\left[\left(f_x^{*}\frac{\partial f_x}{\partial\overline{y}}+f_y^{*}\frac{\partial f_y}{\partial\overline{y}}+|q|^{2}f_z^{*}\frac{\partial f_z}{\partial\overline{y}}\right)\hat{e}_y\right] \\
&+\frac{1}{4}\frac{1}{1+|q|^{2}}\operatorname{Im}\left[\left(|q|^{2}h_x^{*}\frac{\partial h_x}{\partial\overline{x}}+|q|^{2}h_y^{*}\frac{\partial h_y}{\partial\overline{x}}+h_z^{*}\frac{\partial h_z}{\partial\overline{x}}\right)\hat{e}_x\right] \\
&+\frac{1}{4}\frac{1}{1+|q|^{2}}\operatorname{Im}\left[\left(|q|^{2}h_x^{*}\frac{\partial h_x}{\partial\overline{y}}+|q|^{2}h_y^{*}\frac{\partial h_y}{\partial\overline{y}}+h_z^{*}\frac{\partial h_z}{\partial\overline{y}}\right)\hat{e}_y\right]
\end{aligned}
\quad . \tag{S6.7}
$$

Let us go further to separate $\vec{P}^{O}$ into four parts.

$$
\vec{P}^{O}=\frac{1}{1+|q|^{2}}\left(|q|^{2}P_x^{O,TE}+P_x^{O,TM}\right)\hat{e}_x+\frac{1}{1+|q|^{2}}\left(|q|^{2}P_y^{O,TE}+P_y^{O,TM}\right)\hat{e}_y\,. \tag{S6.8}
$$

Here, the idea is to collect terms respectively for the TE and TM modes in the two in-plane Cartesian directions $(\overline{x},\overline{y})$.

$$
\begin{cases}
P_x^{O,TE}\equiv\frac{1}{4}\operatorname{Im}\left(h_x^{*}\frac{\partial h_x}{\partial\overline{x}}+h_y^{*}\frac{\partial h_y}{\partial\overline{x}}+f_z^{*}\frac{\partial f_z}{\partial\overline{x}}\right) \\
P_x^{O,TM}\equiv\frac{1}{4}\operatorname{Im}\left(f_x^{*}\frac{\partial f_x}{\partial\overline{x}}+f_y^{*}\frac{\partial f_y}{\partial\overline{x}}+h_z^{*}\frac{\partial h_z}{\partial\overline{x}}\right) \\
P_y^{O,TE}\equiv\frac{1}{4}\operatorname{Im}\left(h_x^{*}\frac{\partial h_x}{\partial\overline{y}}+h_y^{*}\frac{\partial h_y}{\partial\overline{y}}+f_z^{*}\frac{\partial f_z}{\partial\overline{y}}\right) \\
P_y^{O,TM}\equiv\frac{1}{4}\operatorname{Im}\left(f_x^{*}\frac{\partial f_x}{\partial\overline{y}}+f_y^{*}\frac{\partial f_y}{\partial\overline{y}}+h_z^{*}\frac{\partial h_z}{\partial\overline{y}}\right)
\end{cases}
. \tag{S6.9}
$$

Here, the additional superscripts "TE" and "TM" refer respectively to the TE and TM modes. We can check a partial validity of these items by finding that the respective sets (h_x,h_y,f_z) and (f_x,f_y,h_z) appear in the items representing the TE and TM modes, respectively.

Via either Eq. (S1.3) or Eq. (S1.5), the above Eq. (S6.9) can be expressed solely in terms of the two axial components $\left(f_z, h_z\right)$.

$$\begin{cases} P_x^{O,TE} \equiv \frac{1}{4}\mathrm{Im}\left(\frac{\partial f_z^*}{\partial x}\frac{\partial^2 f_z}{\partial x^2} + \frac{\partial f_z^*}{\partial y}\frac{\partial^2 f_z}{\partial x \partial y} + f_z^* \frac{\partial f_z}{\partial x}\right) \\ P_x^{O,TM} \equiv \frac{1}{4}\mathrm{Im}\left(\frac{\partial h_z^*}{\partial x}\frac{\partial^2 h_z}{\partial x^2} + \frac{\partial h_z^*}{\partial y}\frac{\partial^2 h_z}{\partial x \partial y} + h_z^* \frac{\partial h_z}{\partial x}\right) \\ P_y^{O,TE} \equiv \frac{1}{4}\mathrm{Im}\left(\frac{\partial f_z^*}{\partial x}\frac{\partial^2 f_z}{\partial x \partial y} + \frac{\partial f_z^*}{\partial y}\frac{\partial^2 f_z}{\partial y^2} + f_z^* \frac{\partial f_z}{\partial y}\right) \\ P_y^{O,TM} \equiv \frac{1}{4}\mathrm{Im}\left(\frac{\partial h_z^*}{\partial x}\frac{\partial^2 h_z}{\partial x \partial y} + \frac{\partial h_z^*}{\partial y}\frac{\partial^2 h_z}{\partial y^2} + h_z^* \frac{\partial h_z}{\partial y}\right) \end{cases}. \tag{S6.10}$$

We find that the imaginary unit do not explicitly appear in these four defining equations due to self-cancellations. As a consequence, Eqs. (S6.10) holds true for both rotational cases.

In the Cartesian coordinates, Eq. (S6.10) lends itself easily to a vector form. To this goal, the terms in Eq. (S6.10) are plugged back into Eq. (S6.8).

$$\begin{aligned} \vec{P}^O &= \frac{|q|^2}{1+|q|^2}\left(P_x^{O,TE}\hat{e}_x + P_y^{O,TE}\hat{e}_y\right) + \frac{1}{1+|q|^2}\left(P_x^{O,TM}\hat{e}_x + P_y^{O,TM}\hat{e}_y\right) \\ &= \frac{1}{4}\frac{|q|^2}{1+|q|^2}\mathrm{Im}\left[\begin{array}{l} \left(\frac{\partial f_z^*}{\partial x}\frac{\partial^2 f_z}{\partial x^2} + \frac{\partial f_z^*}{\partial y}\frac{\partial^2 f_z}{\partial x \partial y} + f_z^* \frac{\partial f_z}{\partial x}\right)\hat{e}_x \\ +\left(\frac{\partial f_z^*}{\partial x}\frac{\partial^2 f_z}{\partial x \partial y} + \frac{\partial f_z^*}{\partial y}\frac{\partial^2 f_z}{\partial y^2} + f_z^* \frac{\partial f_z}{\partial y}\right)\hat{e}_y \end{array}\right] \\ &+ \frac{1}{4}\frac{1}{1+|q|^2}\mathrm{Im}\left[\begin{array}{l} \left(\frac{\partial h_z^*}{\partial x}\frac{\partial^2 h_z}{\partial x^2} + \frac{\partial h_z^*}{\partial y}\frac{\partial^2 h_z}{\partial x \partial y} + h_z^* \frac{\partial h_z}{\partial x}\right)\hat{e}_x \\ +\left(\frac{\partial h_z^*}{\partial x}\frac{\partial^2 h_z}{\partial x \partial y} + \frac{\partial h_z^*}{\partial y}\frac{\partial^2 h_z}{\partial y^2} + h_z^* \frac{\partial h_z}{\partial y}\right)\hat{e}_y \end{array}\right] \end{aligned}. \tag{S6.11}$$

At this moment, it is helpful to introduce the following in-plane gradient for a generic vector $\vec{A} \equiv \left(A_x, A_y\right)$ in the Cartesian coordinates.

$$\bar{\nabla}_{\perp}\vec{A} \equiv \frac{\partial A_x}{\partial \bar{x}}\hat{e}_x + \frac{\partial A_y}{\partial \bar{y}}\hat{e}_y . \tag{S6.12}$$

Hence, Eq. (S6.11) is reorganized as follows.

$$\begin{aligned}\vec{P}^O &= \frac{1}{4}\frac{|q|^2}{1+|q|^2}\mathrm{Im}\left[\frac{\partial f_z^*}{\partial \bar{x}}\bar{\nabla}_{\perp}\left(\frac{\partial f_z}{\partial \bar{x}}\right) + \frac{\partial f_z^*}{\partial \bar{y}}\bar{\nabla}_{\perp}\left(\frac{\partial f_z}{\partial \bar{y}}\right) + f_z^*\bar{\nabla}_{\perp} f_z\right] \\ &+ \frac{1}{4}\frac{1}{1+|q|^2}\mathrm{Im}\left[\frac{\partial h_z^*}{\partial \bar{x}}\bar{\nabla}_{\perp}\left(\frac{\partial h_z}{\partial \bar{x}}\right) + \frac{\partial h_z^*}{\partial \bar{y}}\bar{\nabla}_{\perp}\left(\frac{\partial h_z}{\partial \bar{y}}\right) + h_z^*\bar{\nabla}_{\perp} h_z\right]\end{aligned} . \tag{S6.13}$$

Let us introduce another differential vector operator.

$$\left(\vec{A}^* \bullet \bar{\nabla}_{\perp}\right)\vec{A} \equiv A_x^*\bar{\nabla}_{\perp}A_x + A_y^*\bar{\nabla}_{\perp}A_y . \tag{S6.14}$$

This form $\left(\vec{A}^* \bullet \bar{\nabla}_{\perp}\right)\vec{A}$ is the convective-derivative vector, which often occurs in classical fluid dynamics. Eq. (S6.13) is then cast into

$$\begin{aligned}\vec{P}^O &= \frac{1}{4}\frac{|q|^2}{1+|q|^2}\mathrm{Im}\left\{\left[\left(\bar{\nabla}_{\perp} f_z^*\right)\bullet\bar{\nabla}_{\perp}\right]\left(\bar{\nabla}_{\perp} f_z\right) + f_z^*\bar{\nabla}_{\perp} f_z\right\} \\ &+ \frac{1}{4}\frac{1}{1+|q|^2}\mathrm{Im}\left\{\left[\left(\bar{\nabla}_{\perp} h_z^*\right)\bullet\bar{\nabla}_{\perp}\right]\left(\bar{\nabla}_{\perp} h_z\right) + h_z^*\bar{\nabla}_{\perp} h_z\right\} \\ &= \frac{1}{4}\frac{|q|^2}{1+|q|^2}\mathrm{Im}\left\{\left[\left(\bar{\nabla}_{\perp} f_z^*\right)\bullet\bar{\nabla}_{\perp} + f_z^*\right]\left(\bar{\nabla}_{\perp} f_z\right)\right\} \\ &+ \frac{1}{4}\frac{1}{1+|q|^2}\mathrm{Im}\left\{\left[\left(\bar{\nabla}_{\perp} h_z^*\right)\bullet\bar{\nabla}_{\perp} + h_z^*\right]\left(\bar{\nabla}_{\perp} h_z\right)\right\}\end{aligned} . \tag{S6.15}$$

This vector form cannot be easily translated into its counterpart in the polar coordinates. The aforementioned special forms $\vec{E}^* \cdot (\nabla)\vec{E} \equiv E_j^*\left(\partial E_j / \partial x_i\right)\hat{e}_i$ and $\vec{H}^* \cdot (\nabla)\vec{H} \equiv H_j^*\left(\partial H_j / \partial x_i\right)\hat{e}_i$ for the orbital flow density (FD) make the current investigation both unique and time-consuming. These forms are unlike the usual gradient and Laplacian operators, for which we find pertinent transformation rules readily from handbooks and

textbooks. The difficulty with these forms arises essentially from the fact that both $\vec{E}^* \cdot (\nabla)\vec{E}$ and $\vec{H}^* \cdot (\nabla)\vec{H}$ refer to differential forms operated not on scalar but on vector quantities. For this purpose, we have expressed the orbital FD in another vector form in (S6.15).

S7. Vector Laplacians

As another example of the operators on vectors, consider the following vector Laplacian for a generic vector $\vec{V}$.

$$\nabla^2\vec{V} \equiv \nabla^2 V_x \hat{e}_x + \nabla^2 V_y \hat{e}_y + \nabla^2 V_z \hat{e}_z , \tag{S7.1}$$

$$\nabla^2 V_x \equiv \left(\frac{\partial^2 V_x}{\partial x^2} + \frac{\partial^2 V_x}{\partial y^2} + \frac{\partial^2 V_x}{\partial y^2} \right) \hat{e}_x \text{, etc.} \tag{S7.2}$$

Here, $\vec{V} \equiv V_x \hat{e}_x + V_y \hat{e}_y + V_z \hat{e}_z$ or $\vec{V} \equiv (V_x, V_y, V_z)$ in a short-hand notation for the Cartesian coordinates. We can cast this vector in the cylindrical coordinates such that $\vec{V} \equiv V_r \hat{e}_r + V_\theta \hat{e}_\theta + V_z \hat{e}_z$ or $\vec{V} \equiv (V_r, V_\theta, V_z)$ in a short-hand notation for the cylindrical coordinates. The mathematical complications associated with $\nabla^2\vec{V}$ is that it is not easy to express $\nabla^2\vec{V}$ in the cylindrical coordinates. For instance, we may assume that $\vec{V} = (\nabla^2 V_r)\hat{e}_r + (\nabla^2 V_\theta)\hat{e}_\theta + (\nabla^2 V_z)\hat{e}_z$ with

$$\nabla^2 \equiv \frac{\partial^2}{\partial r^2} + \frac{1}{r}\frac{\partial}{\partial r} + \frac{1}{r^2}\frac{\partial^2}{\partial \theta^2} + \frac{\partial^2}{\partial z^2} . \tag{S7.3}$$

In this case, what is missing is the terms like the vector gradients $\nabla(\hat{e}_r, \hat{e}_\theta, \hat{e}_z)$ and the vector Laplacian $\nabla^2(\hat{e}_r, \hat{e}_\theta, \hat{e}_z)$. It is because, the unit vectors $(\hat{e}_r, \hat{e}_\theta, \hat{e}_z)$ for the cylindrical coordinates are spatially varying or inhomogeneous. We will get these extra terms in forthcoming Eq. (S7.17).

Instead, we would better deal with Eqs. (S7.1) and (S7.2). In this approach, let us generalize

$V_x \equiv V_x(x,y,z)$ so that it depends on all the three space coordinates. In order translate Eq. (S7.1) in the Cartesian coordinates into that in the cylindrical coordinates, let us consider several transformation rules.

$$\begin{cases} V_x = cV_r - sV_\theta \\ V_y = sV_r + cV_\theta \end{cases}, \tag{S7.4}$$

$$\begin{cases} \hat{e}_x = c\hat{e}_r - s\hat{e}_\theta \\ \hat{e}_y = s\hat{e}_r + c\hat{e}_\theta \end{cases}, \tag{S7.5}$$

$$\begin{cases} \dfrac{\partial}{\partial \overline{x}} = c\dfrac{\partial}{\partial \overline{\rho}} - \dfrac{s}{\overline{\rho}}\dfrac{\partial}{\partial \theta} \\ \dfrac{\partial}{\partial \overline{y}} = s\dfrac{\partial}{\partial \overline{\rho}} + \dfrac{c}{\overline{\rho}}\dfrac{\partial}{\partial \theta} \end{cases}. \tag{S7.6}$$

Here, we employed a short-hand notation for a pair of angles $c \equiv \cos\theta$ and $s \equiv \sin\theta$. All the three transformation rules in Eqs. (S7.4)-(S7.6) have the same transformation matrix, namely, $\begin{pmatrix} c & -s \\ s & c \end{pmatrix}$, which has a unit determinant.

Now, $\nabla^2\vec{V} \equiv \nabla^2 V_x\hat{e}_x + \nabla^2 V_y\hat{e}_y + \nabla^2 V_z\hat{e}_z$ in Eq. (S7.1) is expanded in full as follows.

$$\begin{aligned} \vec{V} &\equiv \nabla^2 V_x\hat{e}_x + \nabla^2 V_y\hat{e}_y + \nabla^2 V_z\hat{e}_z \\ &= \left(\frac{\partial^2 V_x}{\partial x^2} + \frac{\partial^2 V_x}{\partial y^2} + \frac{\partial^2 V_x}{\partial y^2}\right)\hat{e}_x + \left(\frac{\partial^2 V_y}{\partial x^2} + \frac{\partial^2 V_y}{\partial y^2} + \frac{\partial^2 V_y}{\partial y^2}\right)\hat{e}_y + \left(\frac{\partial^2 V_z}{\partial x^2} + \frac{\partial^2 V_z}{\partial y^2} + \frac{\partial^2 V_z}{\partial y^2}\right)\hat{e}_z \\ &= \left(\frac{\partial^2 V_x}{\partial r^2} + \frac{1}{r}\frac{\partial V_x}{\partial r} + \frac{1}{r^2}\frac{\partial^2 V_x}{\partial \theta^2} + \frac{\partial^2 V_x}{\partial z^2}\right)\hat{e}_x + \left(\frac{\partial^2 V_y}{\partial r^2} + \frac{1}{r}\frac{\partial V_y}{\partial r} + \frac{1}{r^2}\frac{\partial^2 V_y}{\partial \theta^2} + \frac{\partial^2 V_y}{\partial z^2}\right)\hat{e}_y \\ &+ \left(\frac{\partial^2 V_z}{\partial r^2} + \frac{1}{r}\frac{\partial V_z}{\partial r} + \frac{1}{r^2}\frac{\partial^2 V_z}{\partial \theta^2} + \frac{\partial^2 V_z}{\partial z^2}\right)\hat{e}_z \end{aligned}. \tag{S7.7}$$

This step works fine, since $\nabla^2 V_x$ and the likes are the scalar Laplacians, and therefore they can be written in polar coordinates without incurring any errors.

Since the axial term $\nabla^2 V_z\hat{e}_z$ does not change even in the polar coordinates, it will be not

considered any further. With the help of Eqs. (S7.4) and (S7.5), the first two terms $\nabla^2 V_x \hat{e}_x + \nabla^2 V_y \hat{e}_y$ of Eq. (7.7) is now transformed as follows.

$$\begin{aligned}
&\nabla^2 V_x \hat{e}_x + \nabla^2 V_y \hat{e}_y \\
&= \left[\frac{\partial^2 \left(cV_r - sV_\theta \right)}{\partial r^2} + \frac{1}{r} \frac{\partial \left(cV_r - sV_\theta \right)}{\partial r} + \frac{1}{r^2} \frac{\partial^2 \left(cV_r - sV_\theta \right)}{\partial \theta^2} + \frac{\partial^2 \left(cV_r - sV_\theta \right)}{\partial z^2} \right] \left(c\hat{e}_r - s\hat{e}_\theta \right) \\
&+ \left[\frac{\partial^2 \left(sV_r + cV_\theta \right)}{\partial r^2} + \frac{1}{r} \frac{\partial \left(sV_r + cV_\theta \right)}{\partial r} + \frac{1}{r^2} \frac{\partial^2 \left(sV_r + cV_\theta \right)}{\partial \theta^2} + \frac{\partial^2 \left(sV_r + cV_\theta \right)}{\partial z^2} \right] \left(s\hat{e}_r + c\hat{e}_\theta \right)
\end{aligned} \quad \text{(S7.8)}$$

At this point, let us define

$$\nabla^2 V_x \hat{e}_x + \nabla^2 V_y \hat{e}_y \equiv D_r \hat{e}_r + D_\theta \hat{e}_\theta . \quad \text{(S7.9)}$$

Collecting terms of the similar sorts in Eq. (S7.8), we ascertain

$$\begin{cases}
D_r \equiv \dfrac{\partial^2 \left(c^2 V_r - csV_\theta \right)}{\partial r^2} + \dfrac{1}{r} \dfrac{\partial \left(c^2 V_r - csV_\theta \right)}{\partial r} + \dfrac{c}{r^2} \dfrac{\partial^2 \left(cV_r - sV_\theta \right)}{\partial \theta^2} + \dfrac{\partial^2 \left(c^2 V_r - csV_\theta \right)}{\partial z^2} \\
+ \dfrac{\partial^2 \left(s^2 V_r + csV_\theta \right)}{\partial r^2} + \dfrac{1}{r} \dfrac{\partial \left(s^2 V_r + csV_\theta \right)}{\partial r} + \dfrac{s}{r^2} \dfrac{\partial^2 \left(sV_r + cV_\theta \right)}{\partial \theta^2} + \dfrac{\partial^2 \left(s^2 V_r + csV_\theta \right)}{\partial z^2} \\
D_\theta \equiv \dfrac{\partial^2 \left(-csV_r + s^2 V_\theta \right)}{\partial r^2} + \dfrac{1}{r} \dfrac{\partial \left(-csV_r + s^2 V_\theta \right)}{\partial r} + \dfrac{-s}{r^2} \dfrac{\partial^2 \left(cV_r - sV_\theta \right)}{\partial \theta^2} + \dfrac{\partial^2 \left(-csV_r + s^2 V_\theta \right)}{\partial z^2} \\
+ \dfrac{\partial^2 \left(csV_r + c^2 V_\theta \right)}{\partial r^2} + \dfrac{1}{r} \dfrac{\partial \left(csV_r + c^2 V_\theta \right)}{\partial r} + \dfrac{c}{r^2} \dfrac{\partial^2 \left(sV_r + cV_\theta \right)}{\partial \theta^2} + \dfrac{\partial^2 \left(csV_r + c^2 V_\theta \right)}{\partial z^2}
\end{cases} . \quad \text{(S7.10)}$$

Exploiting several cancellations, we obtain

$$\begin{cases}
D_r = \dfrac{\partial^2 V_r}{\partial r^2} + \dfrac{1}{r} \dfrac{\partial V_r}{\partial r} + \dfrac{\partial^2 V_r}{\partial z^2} + \dfrac{c}{r^2} \dfrac{\partial^2 \left(cV_r - sV_\theta \right)}{\partial \theta^2} + \dfrac{s}{r^2} \dfrac{\partial^2 \left(sV_r + cV_\theta \right)}{\partial \theta^2} \\
D_\theta = \dfrac{\partial^2 V_\theta}{\partial r^2} + \dfrac{1}{r} \dfrac{\partial V_\theta}{\partial r} + \dfrac{\partial^2 V_\theta}{\partial z^2} + \dfrac{-s}{r^2} \dfrac{\partial^2 \left(cV_r - sV_\theta \right)}{\partial \theta^2} + \dfrac{c}{r^2} \dfrac{\partial^2 \left(sV_r + cV_\theta \right)}{\partial \theta^2}
\end{cases} . \quad \text{(S7.11)}$$

While proceeding, we need to pay a special attention to the factors like $\left(\partial c / \partial \theta \right) \equiv \left(\partial / \partial \theta \right) \left(\cos\theta \right) = -\sin\theta$.

$$\begin{cases} D_r = \dfrac{\partial^2 V_r}{\partial r^2} + \dfrac{1}{r}\dfrac{\partial V_r}{\partial r} + \dfrac{\partial^2 V_r}{\partial z^2} + \dfrac{c}{r^2}\dfrac{\partial}{\partial\theta}\dfrac{\partial\left(cV_r - sV_\theta\right)}{\partial\theta} + \dfrac{s}{r^2}\dfrac{\partial}{\partial\theta}\dfrac{\partial\left(sV_r + cV_\theta\right)}{\partial\theta} \\ D_\theta = \dfrac{\partial^2 V_\theta}{\partial r^2} + \dfrac{1}{r}\dfrac{\partial V_\theta}{\partial r} + \dfrac{\partial^2 V_\theta}{\partial z^2} + \dfrac{-s}{r^2}\dfrac{\partial}{\partial\theta}\dfrac{\partial\left(cV_r - sV_\theta\right)}{\partial\theta} + \dfrac{c}{r^2}\dfrac{\partial}{\partial\theta}\dfrac{\partial\left(sV_r + cV_\theta\right)}{\partial\theta} \end{cases}. \tag{S7.12}$$

We take derivatives of the above with respect to the azimuthal angle to obtain

$$\begin{aligned} &\nabla^2 V_x \hat{e}_x + \nabla^2 V_y \hat{e}_y \equiv D_r \hat{e}_r + D_\theta \hat{e}_\theta \\ &= \begin{bmatrix} \dfrac{\partial^2 V_r}{\partial r^2} + \dfrac{1}{r}\dfrac{\partial V_r}{\partial r} + \dfrac{\partial^2 V_r}{\partial z^2} + \dfrac{c}{r^2}\dfrac{\partial}{\partial\theta}\left(-sV_r - cV_\theta + c\dfrac{\partial V_r}{\partial\theta} - s\dfrac{\partial V_\theta}{\partial\theta}\right) \\ + \dfrac{s}{r^2}\dfrac{\partial}{\partial\theta}\left(cV_r - sV_\theta + s\dfrac{\partial V_r}{\partial\theta} + c\dfrac{\partial V_\theta}{\partial\theta}\right) \end{bmatrix}\hat{e}_r \\ &+ \begin{bmatrix} \dfrac{\partial^2 V_\theta}{\partial r^2} + \dfrac{1}{r}\dfrac{\partial V_\theta}{\partial r} + \dfrac{\partial^2 V_\theta}{\partial z^2} + \dfrac{-s}{r^2}\dfrac{\partial}{\partial\theta}\left(-sV_r - cV_\theta + c\dfrac{\partial V_r}{\partial\theta} - s\dfrac{\partial V_\theta}{\partial\theta}\right) \\ + \dfrac{c}{r^2}\dfrac{\partial}{\partial\theta}\left(cV_r - sV_\theta + s\dfrac{\partial V_r}{\partial\theta} + c\dfrac{\partial V_\theta}{\partial\theta}\right) \end{bmatrix}\hat{e}_\theta \end{aligned}. \tag{S7.13}$$

Taking the azimuthal derivatives once more,

$$\begin{aligned} &\nabla^2 V_x \hat{e}_x + \nabla^2 V_y \hat{e}_y \equiv D_r \hat{e}_r + D_\theta \hat{e}_\theta \\ &= \begin{bmatrix} \dfrac{\partial^2 V_r}{\partial r^2} + \dfrac{1}{r}\dfrac{\partial V_r}{\partial r} + \dfrac{\partial^2 V_r}{\partial z^2} \\ + \dfrac{c}{r^2}\left(-cV_r + sV_\theta - s\dfrac{\partial V_r}{\partial\theta} - c\dfrac{\partial V_\theta}{\partial\theta} - s\dfrac{\partial V_r}{\partial\theta} - c\dfrac{\partial V_\theta}{\partial\theta} + c\dfrac{\partial^2 V_r}{\partial\theta^2} - s\dfrac{\partial^2 V_\theta}{\partial\theta^2}\right) \\ + \dfrac{s}{r^2}\left(-sV_r - cV_\theta + c\dfrac{\partial V_r}{\partial\theta} - s\dfrac{\partial V_\theta}{\partial\theta} + c\dfrac{\partial V_r}{\partial\theta} - s\dfrac{\partial V_\theta}{\partial\theta} + s\dfrac{\partial^2 V_r}{\partial\theta^2} + c\dfrac{\partial^2 V_\theta}{\partial\theta^2}\right) \end{bmatrix}\hat{e}_r \\ &+ \begin{bmatrix} \dfrac{\partial^2 V_\theta}{\partial r^2} + \dfrac{1}{r}\dfrac{\partial V_\theta}{\partial r} + \dfrac{\partial^2 V_\theta}{\partial z^2} \\ + \dfrac{-s}{r^2}\left(-cV_r + sV_\theta - s\dfrac{\partial V_r}{\partial\theta} - c\dfrac{\partial V_\theta}{\partial\theta} - s\dfrac{\partial V_r}{\partial\theta} - c\dfrac{\partial V_\theta}{\partial\theta} + c\dfrac{\partial^2 V_r}{\partial\theta^2} - s\dfrac{\partial^2 V_\theta}{\partial\theta^2}\right) \\ + \dfrac{c}{r^2}\left(-sV_r - cV_\theta + c\dfrac{\partial V_r}{\partial\theta} - s\dfrac{\partial V_\theta}{\partial\theta} + c\dfrac{\partial V_r}{\partial\theta} - s\dfrac{\partial V_\theta}{\partial\theta} + s\dfrac{\partial^2 V_r}{\partial\theta^2} + c\dfrac{\partial^2 V_\theta}{\partial\theta^2}\right) \end{bmatrix}\hat{e}_\theta \end{aligned}. \tag{S7.14}$$

Via the trigonometric equality $c^2+s^2=1$, the final simplification leads to

$$\begin{aligned}
&\nabla^2 V_x\hat{e}_x+\nabla^2 V_y\hat{e}_y \equiv D_r\hat{e}_r+D_\theta\hat{e}_\theta \\
&=\left(\frac{\partial^2 V_r}{\partial r^2}+\frac{1}{r}\frac{\partial V_r}{\partial r}+\frac{1}{r^2}\frac{\partial^2 V_r}{\partial\theta^2}+\frac{\partial^2 V_r}{\partial z^2}-\frac{V_r}{r^2}-\frac{2}{r^2}\frac{\partial V_\theta}{\partial\theta}\right)\hat{e}_r \; . \\
&+\left(\frac{\partial^2 V_\theta}{\partial r^2}+\frac{1}{r}\frac{\partial V_\theta}{\partial r}+\frac{1}{r^2}\frac{\partial^2 V_\theta}{\partial\theta^2}+\frac{\partial^2 V_\theta}{\partial z^2}-\frac{V_\theta}{r^2}+\frac{2}{r^2}\frac{\partial V_r}{\partial\theta}\right)\hat{e}_\theta
\end{aligned} \tag{S7.15}$$

Here, there are two extra terms. Both $-V_r/r^2$ and $-V_\theta/r^2$ are the centripetal terms. In addition, both $-2r^{-2}(\partial V_\theta/\partial\theta)$ and $+2r^{-2}(\partial V_r/\partial\theta)$ are the Corioli's terms. The equal-signed feature of the centripetal terms in the radial direction recurs in our photon problem. In a similar vein, the opposite-signed feature of the Corioli's terms in the azimuthal direction recurs in our photon problem. Eq. (S7.15) is nothing but

$$\nabla^2 V_x\hat{e}_x+\nabla^2 V_y\hat{e}_y=\left(\nabla^2 V_r-\frac{V_r}{r^2}-\frac{2}{r^2}\frac{\partial V_\theta}{\partial\theta}\right)\hat{e}_r+\left(\nabla^2 V_\theta-\frac{V_\theta}{r^2}+\frac{2}{r^2}\frac{\partial V_r}{\partial\theta}\right)\hat{e}_\theta \, . \tag{S7.16}$$

Meanwhile, Eq. (S7.15) is cast sometimes in the following form.

$$\begin{aligned}
&\nabla^2 V_x\hat{e}_x+\nabla^2 V_y\hat{e}_y \equiv D_r\hat{e}_r+D_\theta\hat{e}_\theta \\
&=\left\{\frac{\partial}{\partial r}\left[\frac{1}{r}\frac{\partial(rV_r)}{\partial r}\right]+\frac{1}{r^2}\frac{\partial^2 V_r}{\partial\theta^2}+\frac{\partial^2 V_r}{\partial z^2}-\frac{2}{r^2}\frac{\partial V_\theta}{\partial\theta}\right\}\hat{e}_r \; . \\
&+\left\{\frac{\partial}{\partial r}\left[\frac{1}{r}\frac{\partial(rV_\theta)}{\partial r}\right]\frac{1}{r^2}\frac{\partial^2 V_\theta}{\partial\theta^2}+\frac{\partial^2 V_\theta}{\partial z^2}+\frac{2}{r^2}\frac{\partial V_r}{\partial\theta}\right\}\hat{e}_\theta
\end{aligned} \tag{S7.17}$$

Eq. (S7.17) is in a more conservation-like form than Eq. (S7.15), although Eq. (S7.17) is still in a non-conservation form. This non-conservative nature of the vector Laplacian $\nabla^2\vec{V}$ in Eq. (S7.1) gave rise to all the extra terms, and it render our investigation into orbital part of angular momentum both difficult and challenging. We remark that this kind of complication arises from $\nabla^2\vec{V}$, the vector Laplacian, in contrary to the conventional scalar Laplacian. In a similar context, we have encountered the convective-derivative vector $(\bar{\nabla}_\perp f_z)\bullet\bar{\nabla}_\perp$ and $(\bar{\nabla}_\perp h_z)\bullet\bar{\nabla}_\perp$ in Eq. (S6.15) for the orbital FD.

We encounter this vector Laplacian while treating the interaction of charged particles with the electromagnetic field, where the vector potential shows up through the kinetic momentum term in the Hamiltonian for electrons. This Dirac equation or the Schrödinger equation in the presence of an external axial magnetic field helps to establish the discrete Landau levels. It is no surprise that the resulting formulation obtained in the condensed-matter physics help to constitute the basic model for what is recently called topological photonics. In the case of the Navier-Stokes equations in classical fluid dynamics, the vector Laplacian $\nabla^2\vec{V}$ appears as the diffusion of the velocity vector $\vec{V}$.

S8. Orbital Flow Density for the Rotating Cases

We remark that both rotating cases cannot be resolved in the Cartesian coordinates in a proper way. In order to differentiate between the co- and counter-rotating cases, we should resort hence to the polar coordinates. To this goal, let us treat Eq. (S6.8) given in the Cartesian coordinates into the polar coordinates via Eq. (S7.5).

$$
\begin{aligned}
\vec{P}^O &= \frac{1}{1+|q|^2}\left(|q|^2 P_x^{O,TE} + P_x^{O,TM}\right)\left(c\hat{e}_r - s\hat{e}_\theta\right) \\
&\quad + \frac{1}{1+|q|^2}\left(|q|^2 P_y^{O,TE} + P_y^{O,TM}\right)\left(s\hat{e}_r + c\hat{e}_\theta\right) \\
&= \frac{1}{1+|q|^2}\left(c|q|^2 P_x^{O,TE} + s|q|^2 P_y^{O,TE} + cP_x^{O,TM} + sP_y^{O,TM}\right)\hat{e}_r \\
&\quad + \frac{1}{1+|q|^2}\left(-s|q|^2 P_x^{O,TE} + c|q|^2 P_y^{O,TE} - sP_x^{O,TM} + cP_y^{O,TM}\right)\hat{e}_\theta \\
&= \frac{1}{1+|q|^2}\left[|q|^2\left(cP_x^{O,TE} + sP_y^{O,TE}\right) + cP_x^{O,TM} + sP_y^{O,TM}\right]\hat{e}_r \\
&\quad + \frac{1}{1+|q|^2}\left[|q|^2\left(-sP_x^{O,TE} + cP_y^{O,TE}\right) - sP_x^{O,TM} + cP_y^{O,TM}\right]\hat{e}_\theta
\end{aligned}
\quad . \qquad \text{(S8.1)}
$$

Our goal is to equate the above relation to the following relation in the polar coordinates.

$$\vec{P}^{O} = \frac{1}{1+|q|^2}\left(|q|^2 P_r^{O,TE} + P_r^{O,TM}\right)\hat{e}_r + \frac{1}{1+|q|^2}\left(|q|^2 P_\theta^{O,TE} + P_\theta^{O,TM}\right)\hat{e}_\theta . \tag{S8.2}$$

Therefore, the four coefficient functions in the polar coordinates are related to the other four in the Cartesian coordinates in the following way.

$$\begin{cases} P_r^{O,TE} = cP_x^{O,TE} + sP_y^{O,TE} \\ P_r^{O,TM} = cP_x^{O,TM} + sP_y^{O,TM} \\ P_\theta^{O,TE} = -sP_x^{O,TE} + cP_y^{O,TE} \\ P_\theta^{O,TM} = -sP_x^{O,TM} + cP_y^{O,TM} \end{cases} . \tag{S8.3}$$

Obviously, the transformation between $\left(P_x^{O,TE}, P_y^{O,TE}\right)$ and $\left(P_r^{O,TE}, P_\theta^{O,TE}\right)$ is the same as that between $\left(P_x^{O,TM}, P_y^{O,TM}\right)$ and $\left(P_r^{O,TM}, P_\theta^{O,TM}\right)$.

Consider the four members separately. First,

$$P_r^{O,TE} \equiv \tfrac{1}{4}\mathrm{Im}\left(\tilde{P}_r^{O,TE}\right) = cP_x^{O,TE} + sP_y^{O,TE} , \tag{S8.4}$$

$$\tilde{P}_r^{O,TE} \equiv c\left(\frac{\partial f_z^*}{\partial \overline{x}}\frac{\partial^2 f_z}{\partial \overline{x}^2} + \frac{\partial f_z^*}{\partial \overline{y}}\frac{\partial^2 f_z}{\partial \overline{x}\partial \overline{y}} + f_z^*\frac{\partial f_z}{\partial \overline{x}}\right) + s\left(\frac{\partial f_z^*}{\partial \overline{x}}\frac{\partial^2 f_z}{\partial \overline{x}\partial \overline{y}} + \frac{\partial f_z^*}{\partial \overline{y}}\frac{\partial^2 f_z}{\partial \overline{y}^2} + f_z^*\frac{\partial f_z}{\partial \overline{y}}\right). \tag{S8.5}$$

Via Eq. (S7.6), the following common operator is further treated.

$$\begin{aligned} c\frac{\partial}{\partial \overline{x}} + s\frac{\partial}{\partial \overline{y}} &= c\left(c\frac{\partial}{\partial \overline{\rho}} - \frac{s}{\overline{\rho}}\frac{\partial}{\partial \theta}\right) + s\left(s\frac{\partial}{\partial \overline{\rho}} + \frac{c}{\overline{\rho}}\frac{\partial}{\partial \theta}\right) \\ &= c^2\frac{\partial}{\partial \overline{\rho}} - \frac{cs}{\overline{\rho}}\frac{\partial}{\partial \theta} + s^2\frac{\partial}{\partial \overline{\rho}} + \frac{cs}{\overline{\rho}}\frac{\partial}{\partial \theta} = \left(c^2 + s^2\right)\frac{\partial}{\partial \overline{\rho}} = \frac{\partial}{\partial \overline{\rho}} \end{aligned} . \tag{S8.6}$$

Hence, we proceed with Eq. (S8.5) while keeping the order of differentiations to obtain

$$
\begin{aligned}
\tilde{P}_r^{O,TE} &= \frac{\partial f_z^*}{\partial \bar{x}}\frac{\partial}{\partial \bar{\rho}}\frac{\partial f_z}{\partial \bar{x}} + \frac{\partial f_z^*}{\partial \bar{y}}\frac{\partial}{\partial \bar{\rho}}\frac{\partial f_z}{\partial \bar{y}} + f_z^*\frac{\partial f_z}{\partial \bar{\rho}} \\
&= \left(c\frac{\partial f_z^*}{\partial \bar{\rho}} - \frac{s}{\bar{\rho}}\frac{\partial f_z^*}{\partial \theta}\right)\frac{\partial}{\partial \bar{\rho}}\left(c\frac{\partial f_z}{\partial \bar{\rho}} - \frac{s}{\bar{\rho}}\frac{\partial f_z}{\partial \theta}\right) \\
&+ \left(s\frac{\partial f_z^*}{\partial \bar{\rho}} + \frac{c}{\bar{\rho}}\frac{\partial f_z^*}{\partial \theta}\right)\frac{\partial}{\partial \bar{\rho}}\left(s\frac{\partial f_z}{\partial \bar{\rho}} + \frac{c}{\bar{\rho}}\frac{\partial f_z}{\partial \theta}\right) + f_z^*\frac{\partial f_z}{\partial \bar{\rho}} \\
&= \left(c\frac{\partial f_z^*}{\partial \bar{\rho}} - \frac{s}{\bar{\rho}}\frac{\partial f_z^*}{\partial \theta}\right)\left(c\frac{\partial^2 f_z}{\partial \bar{\rho}^2} - \frac{s}{\bar{\rho}}\frac{\partial^2 f_z}{\partial \bar{\rho}\partial \theta} + \frac{s}{\bar{\rho}^2}\frac{\partial f_z}{\partial \theta}\right) \\
&+ \left(s\frac{\partial f_z^*}{\partial \bar{\rho}} + \frac{c}{\bar{\rho}}\frac{\partial f_z^*}{\partial \theta}\right)\left(s\frac{\partial^2 f_z}{\partial \bar{\rho}^2} + \frac{c}{\bar{\rho}}\frac{\partial^2 f_z}{\partial \bar{\rho}\partial \theta} - \frac{c}{\bar{\rho}^2}\frac{\partial f_z}{\partial \theta}\right) + f_z^*\frac{\partial f_z}{\partial \bar{\rho}}
\end{aligned}
\quad . \qquad \text{(S8.7)}
$$

It is simplified further.

$$
\begin{aligned}
\tilde{P}_r^{O,TE} &= c^2\frac{\partial f_z^*}{\partial \bar{\rho}}\frac{\partial^2 f_z}{\partial \bar{\rho}^2} - \frac{cs}{\bar{\rho}}\frac{\partial f_z^*}{\partial \bar{\rho}}\frac{\partial^2 f_z}{\partial \bar{\rho}\partial \theta} + \frac{cs}{\bar{\rho}^2}\frac{\partial f_z^*}{\partial \bar{\rho}}\frac{\partial f_z}{\partial \theta} \\
&- \frac{cs}{\bar{\rho}}\frac{\partial f_z^*}{\partial \theta}\frac{\partial^2 f_z}{\partial \bar{\rho}^2} + \frac{s^2}{\bar{\rho}^2}\frac{\partial f_z^*}{\partial \theta}\frac{\partial^2 f_z}{\partial \bar{\rho}\partial \theta} - \frac{s^2}{\bar{\rho}^3}\frac{\partial f_z^*}{\partial \theta}\frac{\partial f_z}{\partial \theta} \\
&+ s^2\frac{\partial f_z^*}{\partial \bar{\rho}}\frac{\partial^2 f_z}{\partial \bar{\rho}^2} + \frac{cs}{\bar{\rho}}\frac{\partial f_z^*}{\partial \bar{\rho}}\frac{\partial^2 f_z}{\partial \bar{\rho}\partial \theta} - \frac{cs}{\bar{\rho}^2}\frac{\partial f_z^*}{\partial \bar{\rho}}\frac{\partial f_z}{\partial \theta} \\
&+ \frac{cs}{\bar{\rho}}\frac{\partial f_z^*}{\partial \theta}\frac{\partial^2 f_z}{\partial \bar{\rho}^2} + \frac{c^2}{\bar{\rho}^2}\frac{\partial f_z^*}{\partial \theta}\frac{\partial^2 f_z}{\partial \bar{\rho}\partial \theta} - \frac{c^2}{\bar{\rho}^3}\frac{\partial f_z^*}{\partial \theta}\frac{\partial f_z}{\partial \theta} + f_z^*\frac{\partial f_z}{\partial \bar{\rho}}
\end{aligned}
\quad . \qquad \text{(S8.8)}
$$

It simplifies once again to the final relation, thus leading to no explicit appearance of the azimuthal angle.

$$
\begin{cases}
\tilde{P}_r^{O,TE} = \dfrac{\partial f_z^*}{\partial \bar{\rho}}\dfrac{\partial^2 f_z}{\partial \bar{\rho}^2} + \dfrac{1}{\bar{\rho}^2}\dfrac{\partial f_z^*}{\partial \theta}\dfrac{\partial^2 f_z}{\partial \bar{\rho}\partial \theta} - \dfrac{1}{\bar{\rho}^3}\dfrac{\partial f_z^*}{\partial \theta}\dfrac{\partial f_z}{\partial \theta} + f_z^*\dfrac{\partial f_z}{\partial \bar{\rho}} \\
\tilde{P}_r^{O,TM} = \dfrac{\partial h_z^*}{\partial \bar{\rho}}\dfrac{\partial^2 h_z}{\partial \bar{\rho}^2} + \dfrac{1}{\bar{\rho}^2}\dfrac{\partial h_z^*}{\partial \theta}\dfrac{\partial^2 h_z}{\partial \bar{\rho}\partial \theta} - \dfrac{1}{\bar{\rho}^3}\dfrac{\partial h_z^*}{\partial \theta}\dfrac{\partial h_z}{\partial \theta} + h_z^*\dfrac{\partial h_z}{\partial \bar{\rho}}
\end{cases}
. \qquad \text{(S8.9)}
$$

For the second relation in Eq. (S8.9), we have taken an advantage of the symmetry between the TE and TM modes.

Second, the preceding entire procedure is repeated in the azimuthal direction.

$$P_\theta^{O,TE} \equiv \tfrac{1}{4}\mathrm{Im}\left(\tilde{P}_\theta^{O,TE}\right) = -sP_x^{O,TE} + cP_y^{O,TE}, \tag{S8.10}$$

$$\tilde{P}_\theta^{O,TE} \equiv -s\left(\frac{\partial f_z^*}{\partial \overline{x}}\frac{\partial^2 f_z}{\partial \overline{x}^2} + \frac{\partial f_z^*}{\partial \overline{y}}\frac{\partial^2 f_z}{\partial \overline{x}\partial \overline{y}} + f_z^*\frac{\partial f_z}{\partial \overline{x}}\right) + c\left(\frac{\partial f_z^*}{\partial \overline{x}}\frac{\partial^2 f_z}{\partial \overline{x}\partial \overline{y}} + \frac{\partial f_z^*}{\partial \overline{y}}\frac{\partial^2 f_z}{\partial \overline{y}^2} + f_z^*\frac{\partial f_z}{\partial \overline{y}}\right). \tag{S8.11}$$

Via Eq. (S7.6), the combination operator becomes now the azimuthal derivative.

$$\begin{aligned} &-s\frac{\partial}{\partial \overline{x}} + c\frac{\partial}{\partial \overline{y}} = -s\left(c\frac{\partial}{\partial \overline{\rho}} - \frac{s}{\overline{\rho}}\frac{\partial}{\partial \theta}\right) + c\left(s\frac{\partial}{\partial \overline{\rho}} + \frac{c}{\overline{\rho}}\frac{\partial}{\partial \theta}\right) \\ &= -cs\frac{\partial}{\partial \overline{\rho}} + \frac{s^2}{\overline{\rho}}\frac{\partial}{\partial \theta} + cs\frac{\partial}{\partial \overline{\rho}} + \frac{c^2}{\overline{\rho}}\frac{\partial}{\partial \theta} = \left(c^2 + s^2\right)\frac{1}{\overline{\rho}}\frac{\partial}{\partial \theta} = \frac{1}{\overline{\rho}}\frac{\partial}{\partial \theta} \end{aligned}. \tag{S8.12}$$

Hence, we proceed with Eq. (S8.11) while keeping the order of differentiations to obtain

$$\begin{aligned} \tilde{P}_\theta^{O,TE} &= \frac{\partial f_z^*}{\partial \overline{x}}\frac{1}{\overline{\rho}}\frac{\partial}{\partial \theta}\frac{\partial f_z}{\partial \overline{x}} + \frac{\partial f_z^*}{\partial \overline{y}}\frac{1}{\overline{\rho}}\frac{\partial}{\partial \theta}\frac{\partial f_z}{\partial \overline{y}} + f_z^*\frac{1}{\overline{\rho}}\frac{\partial f_z}{\partial \theta} \\ &= \left(c\frac{\partial f_z^*}{\partial \overline{\rho}} - \frac{s}{\overline{\rho}}\frac{\partial f_z^*}{\partial \theta}\right)\frac{1}{\overline{\rho}}\frac{\partial}{\partial \theta}\left(c\frac{\partial f_z}{\partial \overline{\rho}} - \frac{s}{\overline{\rho}}\frac{\partial f_z}{\partial \theta}\right) \\ &+ \left(s\frac{\partial f_z^*}{\partial \overline{\rho}} + \frac{c}{\overline{\rho}}\frac{\partial f_z^*}{\partial \theta}\right)\frac{1}{\overline{\rho}}\frac{\partial}{\partial \theta}\left(s\frac{\partial f_z}{\partial \overline{\rho}} + \frac{c}{\overline{\rho}}\frac{\partial f_z}{\partial \theta}\right) + f_z^*\frac{1}{\overline{\rho}}\frac{\partial f_z}{\partial \theta} \\ &= \left(c\frac{\partial f_z^*}{\partial \overline{\rho}} - \frac{s}{\overline{\rho}}\frac{\partial f_z^*}{\partial \theta}\right)\left(c\frac{1}{\overline{\rho}}\frac{\partial^2 f_z}{\partial \overline{\rho}\partial \theta} - \frac{s}{\overline{\rho}^2}\frac{\partial^2 f_z}{\partial \theta^2}\right) \\ &+ \left(s\frac{\partial f_z^*}{\partial \overline{\rho}} + \frac{c}{\overline{\rho}}\frac{\partial f_z^*}{\partial \theta}\right)\left(s\frac{1}{\overline{\rho}}\frac{\partial^2 f_z}{\partial \overline{\rho}\partial \theta} + \frac{c}{\overline{\rho}^2}\frac{\partial^2 f_z}{\partial \theta^2}\right) + f_z^*\frac{1}{\overline{\rho}}\frac{\partial f_z}{\partial \theta} \end{aligned}. \tag{S8.13}$$

Further simplifying,

$$\begin{aligned} \tilde{P}_\theta^{O,TE} &= \frac{c^2}{\overline{\rho}}\frac{\partial f_z^*}{\partial \overline{\rho}}\frac{\partial^2 f_z}{\partial \overline{\rho}\partial \theta} - \frac{cs}{\overline{\rho}^2}\frac{\partial f_z^*}{\partial \overline{\rho}}\frac{\partial^2 f_z}{\partial \theta^2} - \frac{cs}{\overline{\rho}^2}\frac{\partial f_z^*}{\partial \theta}\frac{\partial^2 f_z}{\partial \overline{\rho}\partial \theta} + \frac{s^2}{\overline{\rho}^3}\frac{\partial f_z^*}{\partial \theta}\frac{\partial^2 f_z}{\partial \theta^2} \\ &+ \frac{s^2}{\overline{\rho}}\frac{\partial f_z^*}{\partial \overline{\rho}}\frac{\partial^2 f_z}{\partial \overline{\rho}\partial \theta} + \frac{cs}{\overline{\rho}^2}\frac{\partial f_z^*}{\partial \overline{\rho}}\frac{\partial^2 f_z}{\partial \theta^2} + \frac{cs}{\overline{\rho}^2}\frac{\partial f_z^*}{\partial \theta}\frac{\partial^2 f_z}{\partial \overline{\rho}\partial \theta} + \frac{c^2}{\overline{\rho}^3}\frac{\partial f_z^*}{\partial \theta}\frac{\partial^2 f_z}{\partial \theta^2} + f_z^*\frac{1}{\overline{\rho}}\frac{\partial f_z}{\partial \theta} \end{aligned}. \tag{S8.14}$$

Finally, we get two relations without any explicit dependence on the azimuthal angle.

$$\begin{cases} \tilde{P}_\theta^{O,TE} = \dfrac{1}{\bar{\rho}}\dfrac{\partial f_z^*}{\partial \bar{\rho}}\dfrac{\partial^2 f_z}{\partial \bar{\rho}\partial \theta} + \dfrac{1}{\bar{\rho}^3}\dfrac{\partial f_z^*}{\partial \theta}\dfrac{\partial^2 f_z}{\partial \theta^2} + f_z^*\dfrac{1}{\bar{\rho}}\dfrac{\partial f_z}{\partial \theta} \\ \tilde{P}_\theta^{O,TM} = \dfrac{1}{\bar{\rho}}\dfrac{\partial h_z^*}{\partial \bar{\rho}}\dfrac{\partial^2 h_z}{\partial \bar{\rho}\partial \theta} + \dfrac{1}{\bar{\rho}^3}\dfrac{\partial h_z^*}{\partial \theta}\dfrac{\partial^2 h_z}{\partial \theta^2} + h_z^*\dfrac{1}{\bar{\rho}}\dfrac{\partial h_z}{\partial \theta} \end{cases}. \tag{S8.15}$$

In the second relation of Eq. (S8.15), we have similarly treated the TM mode. Hence, we have obtained the two desired in-plane components $\vec{P}^O \equiv P_r^O \hat{e}_r + P_\theta^O \hat{e}_\theta$.

Now, we are to replace the azimuthal derivatives with the azimuthal mode index according to $e^{\pm im\theta}$. Let us work on the co-rotational case first. On the basis of the field variables in Eq. (S1.2), we employ $\left(\partial f_z/\partial\theta\right) = imf_z$, $\left(\partial h_z/\partial\theta\right) = imh_z$, $\left(\partial f_z^*/\partial\theta\right) = -imf_z^*$, and $\left(\partial h_z^*/\partial\theta\right) = -imh_z^*$. For the TE mode, $\tilde{P}_r^{O,TE}$ in Eq. (S8.9) becomes

$$\begin{aligned} \tilde{P}_r^{O,TE} &= \frac{\partial f_z^*}{\partial\bar{\rho}}\frac{\partial^2 f_z}{\partial\bar{\rho}^2} + \frac{1}{\bar{\rho}^2}\frac{\partial f_z^*}{\partial\theta}\frac{\partial^2 f_z}{\partial\bar{\rho}\partial\theta} - \frac{1}{\bar{\rho}^3}\frac{\partial f_z^*}{\partial\theta}\frac{\partial f_z}{\partial\theta} + f_z^*\frac{\partial f_z}{\partial\bar{\rho}} \\ &= \frac{\partial f_z^*}{\partial\bar{\rho}}\frac{\partial^2 f_z}{\partial\bar{\rho}^2} + \frac{m^2}{\bar{\rho}^2} f_z^*\frac{\partial f_z}{\partial\bar{\rho}} - \frac{m^2}{\bar{\rho}^3}\left|f_z\right|^2 + f_z^*\frac{\partial f_z}{\partial\bar{\rho}} \\ &= \frac{\partial f_z^*}{\partial\bar{\rho}}\frac{\partial f_z}{\partial\bar{\rho}}\frac{\partial}{\partial\bar{\rho}}\left[\ln\left(\frac{\partial f_z}{\partial\bar{\rho}}\right)\right] + \left(1+\frac{m^2}{\bar{\rho}^2}\right)\left|f_z\right|^2\frac{\partial\ln\left(f_z\right)}{\partial\bar{\rho}} - \frac{m^2}{\bar{\rho}^3}\left|f_z\right|^2 . \\ &= \left|\frac{\partial f_z}{\partial\bar{\rho}}\right|^2\frac{\partial}{\partial\bar{\rho}}\left[\ln\left(\frac{\partial f_z}{\partial\bar{\rho}}\right)\right] + \left(1+\frac{m^2}{\bar{\rho}^2}\right)\left|f_z\right|^2\frac{\partial\ln\left(f_z\right)}{\partial\bar{\rho}} - \frac{m^2}{\bar{\rho}^3}\left|f_z\right|^2 \\ &= \left[\left|\frac{\partial\ln\left(f_z\right)}{\partial\bar{\rho}}\right|^2\frac{\partial}{\partial\bar{\rho}}\left[\ln\left(\frac{\partial f_z}{\partial\bar{\rho}}\right)\right] + \left(1+\frac{m^2}{\bar{\rho}^2}\right)\frac{\partial\ln\left(f_z\right)}{\partial\bar{\rho}} - \frac{m^2}{\bar{\rho}^3}\right]\left|f_z\right|^2 \end{aligned} \tag{S8.16}$$

We treat $\tilde{P}_r^{O,TM}$ in Eq. (S8.8) for the TM mode in a similar way.

$$
\begin{aligned}
\tilde{P}_r^{O,TM} &= \frac{\partial h_z^*}{\partial \bar{\rho}} \frac{\partial^2 h_z}{\partial \bar{\rho}^2} + \frac{1}{\bar{\rho}^2} \frac{\partial h_z^*}{\partial \theta} \frac{\partial^2 h_z}{\partial \bar{\rho} \partial \theta} - \frac{1}{\bar{\rho}^3} \frac{\partial h_z^*}{\partial \theta} \frac{\partial h_z}{\partial \theta} + h_z^* \frac{\partial h_z}{\partial \bar{\rho}} \\
&= \frac{\partial h_z^*}{\partial \bar{\rho}} \frac{\partial^2 h_z}{\partial \bar{\rho}^2} + \frac{m^2}{\bar{\rho}^2} h_z^* \frac{\partial h_z}{\partial \bar{\rho}} - \frac{m^2}{\bar{\rho}^3} h_z^* h_z + h_z^* \frac{\partial h_z}{\partial \bar{\rho}} \\
&= \frac{\partial h_z^*}{\partial \bar{\rho}} \frac{\partial h_z}{\partial \bar{\rho}} \frac{\partial}{\partial \bar{\rho}} \left[\ln\left(\frac{\partial h_z}{\partial \bar{\rho}} \right) \right] + \left(1 + \frac{m^2}{\bar{\rho}^2} \right) |h_z|^2 \frac{\partial \ln(h_z)}{\partial \bar{\rho}} - \frac{m^2}{\bar{\rho}^3} |h_z|^2 \quad . \qquad \text{(S8.17)} \\
&= \left| \frac{\partial h_z}{\partial \bar{\rho}} \right|^2 \frac{\partial}{\partial \bar{\rho}} \left[\ln\left(\frac{\partial h_z}{\partial \bar{\rho}} \right) \right] + \left(1 + \frac{m^2}{\bar{\rho}^2} \right) |h_z|^2 \frac{\partial \ln(h_z)}{\partial \bar{\rho}} - \frac{m^2}{\bar{\rho}^3} |h_z|^2 \\
&= \left[\left| \frac{\partial \ln(h_z)}{\partial \bar{\rho}} \right|^2 \frac{\partial}{\partial \bar{\rho}} \left[\ln\left(\frac{\partial h_z}{\partial \bar{\rho}} \right) \right] + \left(1 + \frac{m^2}{\bar{\rho}^2} \right) \frac{\partial \ln(h_z)}{\partial \bar{\rho}} - \frac{m^2}{\bar{\rho}^3} \right] |h_z|^2
\end{aligned}
$$

In both Eqs. (S8.16) and (S8.17), we notice that the azimuthal derivatives appear even numbers of times. Therefore, Eq. (T6.17) could follows directly from Eq. (T6.16) just by substituting f_z with h_z.

Meanwhile, consider the azimuthal components. For the TE mode, Eq.(S8.15) becomes

$$
\begin{aligned}
\tilde{P}_\theta^{O,TE} &= \frac{1}{\bar{\rho}} \frac{\partial f_z^*}{\partial \bar{\rho}} \frac{\partial^2 f_z}{\partial \bar{\rho} \partial \theta} + \frac{1}{\bar{\rho}^3} \frac{\partial f_z^*}{\partial \theta} \frac{\partial^2 f_z}{\partial \theta^2} + f_z^* \frac{1}{\bar{\rho}} \frac{\partial f_z}{\partial \theta} \\
&= \frac{im}{\bar{\rho}} \frac{\partial f_z^*}{\partial \bar{\rho}} \frac{\partial f_z}{\partial \bar{\rho}} + \frac{im^3}{\bar{\rho}^3} f_z^* f_z + f_z^* \frac{im}{\bar{\rho}} f_z \quad . \qquad \text{(S8.18)} \\
&= i \frac{m}{\bar{\rho}} \left(\frac{\partial f_z^*}{\partial \bar{\rho}} \frac{\partial f_z}{\partial \bar{\rho}} + \frac{m^2}{\bar{\rho}^2} |f_z|^2 + |f_z|^2 \right) = i \frac{m}{\bar{\rho}} \left(\left| \frac{\partial \ln(f_z)}{\partial \bar{\rho}} \right|^2 + \frac{m^2}{\bar{\rho}^2} + 1 \right) |f_z|^2
\end{aligned}
$$

Likewise, for the TM mode, Eq. (S8.15) becomes

$$
\begin{aligned}
\tilde{P}_\theta^{O,TM} &= \frac{1}{\bar{\rho}} \frac{\partial h_z^*}{\partial \bar{\rho}} \frac{\partial^2 h_z}{\partial \bar{\rho} \partial \theta} + \frac{1}{\bar{\rho}^3} \frac{\partial h_z^*}{\partial \theta} \frac{\partial^2 h_z}{\partial \theta^2} + h_z^* \frac{1}{\bar{\rho}} \frac{\partial h_z}{\partial \theta} \\
&= \frac{im}{\bar{\rho}} \frac{\partial h_z^*}{\partial \bar{\rho}} \frac{\partial h_z}{\partial \bar{\rho}} + \frac{im^3}{\bar{\rho}^3} h_z^* h_z + h_z^* \frac{im}{\bar{\rho}} h_z \quad . \qquad \text{(S8.19)} \\
&= i \frac{m}{\bar{\rho}} \left(\frac{\partial h_z^*}{\partial \bar{\rho}} \frac{\partial h_z}{\partial \bar{\rho}} + \frac{m^2}{\bar{\rho}^2} |h_z|^2 + |h_z|^2 \right) = i \frac{m}{\bar{\rho}} \left(\left| \frac{\partial \ln(h_z)}{\partial \bar{\rho}} \right|^2 + \frac{m^2}{\bar{\rho}^2} + 1 \right) |h_z|^2
\end{aligned}
$$

In both Eqs. (S8.18) and (S8.19), we notice that the azimuthal derivatives appear odd numbers of times. Therefore, Eq. (S8.19) could follow directly from Eq. (S8.18) just by substituting f_z with h_z.

Next, consider the counter-rotational case. On the basis of Eq. (S1.4) for the counter-rotational case, we proceed by replacing $\left(\partial f_z/\partial\theta\right)=-imf_z$, $\left(\partial h_z/\partial\theta\right)=imh_z$, $\left(\partial f_z^*/\partial\theta\right)=imf_z^*$, and $\left(\partial h_z^*/\partial\theta\right)=-imh_z^*$. However, the end formulas remain the same as those for the co-rotational cases when it comes to the radial components $\tilde{P}_r^{O,TE}$ and $\tilde{P}_r^{O,TM}$, because the azimuthal derivatives are applied to both a field variable and its conjugate within the identical term. The reason is again that the radial component is akin to the centripetal or centrifugal force so that it is independent of the direction of the azimuthal propagations. That is why we considered Eq. (S7.15) or Eq. (7.16) for a vector Laplacian to explicitly show the appearance of both $-V_r/r^2$ and $-V_\theta/r^2$ of equal signs.

In contrast, let us rework the azimuthal components $\tilde{P}_\theta^{O,TE}$ in Eq. (S8.18) and $\tilde{P}_\theta^{O,TM}$ in Eq. (S8.19) now for the counter-rotational case.

$$\begin{aligned}\tilde{P}_\theta^{O,TE}&=\frac{1}{\bar{\rho}}\frac{\partial f_z^*}{\partial\bar{\rho}}\frac{\partial^2 f_z}{\partial\bar{\rho}\partial\theta}+\frac{1}{\bar{\rho}^3}\frac{\partial f_z^*}{\partial\theta}\frac{\partial^2 f_z}{\partial\theta^2}+f_z^*\frac{1}{\bar{\rho}}\frac{\partial f_z}{\partial\theta}\\&=-\frac{im}{\bar{\rho}}\frac{\partial f_z^*}{\partial\bar{\rho}}\frac{\partial f_z}{\partial\bar{\rho}}-\frac{im^3}{\bar{\rho}^3}f_z^*f_z-f_z^*\frac{im}{\bar{\rho}}f_z\\&=-i\frac{m}{\bar{\rho}}\left(\frac{\partial f_z^*}{\partial\bar{\rho}}\frac{\partial f_z}{\partial\bar{\rho}}+\frac{m^2}{\bar{\rho}^2}\left|f_z\right|^2+\left|f_z\right|^2\right)=-i\frac{m}{\bar{\rho}}\left(\left|\frac{\partial\ln\left(f_z\right)}{\partial\bar{\rho}}\right|^2+\frac{m^2}{\bar{\rho}^2}+1\right)\left|f_z\right|^2\end{aligned}\quad, \tag{S8.20}$$

$$\begin{aligned}\tilde{P}_\theta^{O,TM}&=\frac{1}{\bar{\rho}}\frac{\partial h_z^*}{\partial\bar{\rho}}\frac{\partial^2 h_z}{\partial\bar{\rho}\partial\theta}+\frac{1}{\bar{\rho}^3}\frac{\partial h_z^*}{\partial\theta}\frac{\partial^2 h_z}{\partial\theta^2}+h_z^*\frac{1}{\bar{\rho}}\frac{\partial h_z}{\partial\theta}\\&=\frac{im}{\bar{\rho}}\frac{\partial h_z^*}{\partial\bar{\rho}}\frac{\partial h_z}{\partial\bar{\rho}}+\frac{im^3}{\bar{\rho}^3}h_z^*h_z+h_z^*\frac{im}{\bar{\rho}}h_z\\&=i\frac{m}{\bar{\rho}}\left(\frac{\partial h_z^*}{\partial\bar{\rho}}\frac{\partial h_z}{\partial\bar{\rho}}+\frac{m^2}{\bar{\rho}^2}\left|h_z\right|^2+\left|h_z\right|^2\right)=i\frac{m}{\bar{\rho}}\left(\left|\frac{\partial\ln\left(h_z\right)}{\partial\bar{\rho}}\right|^2+\frac{m^2}{\bar{\rho}^2}+1\right)\left|h_z\right|^2\end{aligned}\quad. \tag{S8.21}$$

Quite obviously, $\tilde{P}_\theta^{O,TE}$ for the counter-rotational case has its sign opposite to that for the co-

rotational case, since we assumed opposite rotating directions. In comparison, $\tilde{P}_\theta^{O,TM}$ remains the same, because we assumed the same rotating direction.

To summarize, the radial components of the orbital FD is found as follows for both rotational cases.

$$\begin{cases} P_r^{O,TE} = \tfrac{1}{4}\left| f_z \right|^2 \mathrm{Im}\left[\left| \frac{\partial \ln\left(f_z \right)}{\partial \bar{\rho}} \right|^2 \frac{\partial}{\partial \bar{\rho}}\left[\ln\left(\frac{\partial f_z}{\partial \bar{\rho}} \right) \right] + \left(1 + \frac{m^2}{\bar{\rho}^2} \right) \frac{\partial \ln\left(f_z \right)}{\partial \bar{\rho}} - \frac{m^2}{\bar{\rho}^3} \right] \\ P_r^{O,TM} = \tfrac{1}{4}\left| h_z \right|^2 \mathrm{Im}\left[\left| \frac{\partial \ln\left(h_z \right)}{\partial \bar{\rho}} \right|^2 \frac{\partial}{\partial \bar{\rho}}\left[\ln\left(\frac{\partial h_z}{\partial \bar{\rho}} \right) \right] + \left(1 + \frac{m^2}{\bar{\rho}^2} \right) \frac{\partial \ln\left(h_z \right)}{\partial \bar{\rho}} - \frac{m^2}{\bar{\rho}^3} \right] \end{cases}. \quad \text{(S8.22)}$$

The sign of the combined radial component cannot be easily figured out from these formulas alone, so that we would find it through numerical means. To this goal, let us now employ the solution profiles $f_z\left(\bar{\rho}\right) = F_m^{\pm}\left(\bar{\rho}\right)$ and $h_z\left(\bar{\rho}\right) = NF_m^{\pm}\left(\bar{\rho}\right)$. In addition, we will make use of the gradient functions $G_m^{\pm}\left(\bar{\rho}\right)$ and $K_m^{\pm}\left(\bar{\rho}\right)$ in Eq. (S1.7) and (S1.8). It will be helpful to recall the simple algebraic fact that $\left(\partial/\partial\bar{\rho}\right)\ln\left(f_z \right) = \left(\partial/\partial\bar{\rho}\right)\ln\left(h_z \right)$ and $\left(\partial/\partial\bar{\rho}\right)\ln\left(\partial f_z/\partial\bar{\rho}\right) = \left(\partial/\partial\bar{\rho}\right)\ln\left(\partial h_z/\partial\bar{\rho}\right)$.

Now, both $\tilde{P}_r^{O,TE}$ and $\tilde{P}_r^{O,TM}$ are expressible in terms of $F_m^{\pm}$, $G_m^{\pm}$, and $K_m^{\pm}$.

$$\begin{cases} P_r^{O,TE} = \tfrac{1}{4}\left| F_m^{\pm} \right|^2 \mathrm{Im}\left[\left| G_m^{\pm} \right|^2 K_m^{\pm} + \left(1 + \frac{m^2}{\bar{\rho}^2} \right) G_m^{\pm} - \frac{m^2}{\bar{\rho}^3} \right] \\ P_r^{O,TM} = N^2 P_r^{O,TE} \end{cases}. \quad \text{(S8.23)}$$

The radial component of the orbital FD is thus given by

$$P_r^O = \frac{1}{4}\frac{N^2 + \left| q \right|^2}{1 + \left| q \right|^2}\left| F_m^{\pm} \right|^2 \mathrm{Im}\left[\left| G_m^{\pm} \right|^2 K_m^{\pm} + \left(1 + \frac{m^2}{\bar{\rho}^2} \right) G_m^{\pm} - \frac{m^2}{\bar{\rho}^3} \right]. \quad \text{(S8.24)}$$

As expected, the argument of $\mathrm{Im}\left[\circ\right]$ in the above relation takes real values in the interior.

Consequently, $P_r^O = 0$ in the interior, where P_θ^O is non-zero. Consequently, the trajectories for the orbital FD are purely circular in the interior.

Let us turn to the azimuthal components. For the co-rotational case, the azimuthal components of the orbital FD is found as follows, when taking the respective imaginary parts.

$$\begin{cases} {}^{<}P_\theta^{O,TE} = \tfrac{1}{4}\dfrac{m}{\bar{\rho}}\left(\left|\dfrac{\partial \ln\left(f_z\right)}{\partial \bar{\rho}}\right|^2 + \dfrac{m^2}{\bar{\rho}^2} + 1\right)\left|f_z\right|^2 \\ {}^{<}P_\theta^{O,TM} = \tfrac{1}{4}\dfrac{m}{\bar{\rho}}\left(\left|\dfrac{\partial \ln\left(h_z\right)}{\partial \bar{\rho}}\right|^2 + \dfrac{m^2}{\bar{\rho}^2} + 1\right)\left|h_z\right|^2 \end{cases} . \qquad \text{(S8.25)}$$

Here, the superscripts "$<$" and "$>$" refer respectively to the clockwise and counter-clockwise rotations.

In comparison, for the counter-rotational case, the azimuthal components of the orbital FD is found as follows, when taking the respective imaginary parts.

$$\begin{cases} {}^{>}P_\theta^{O,TE} = -\tfrac{1}{4}\dfrac{m}{\bar{\rho}}\left(\left|\dfrac{\partial \ln\left(f_z\right)}{\partial \bar{\rho}}\right|^2 + \dfrac{m^2}{\bar{\rho}^2} + 1\right)\left|f_z\right|^2 \\ {}^{<}P_\theta^{O,TM} = \tfrac{1}{4}\dfrac{m}{\bar{\rho}}\left(\left|\dfrac{\partial \ln\left(h_z\right)}{\partial \bar{\rho}}\right|^2 + \dfrac{m^2}{\bar{\rho}^2} + 1\right)\left|h_z\right|^2 \end{cases} . \qquad \text{(S8.26)}$$

Now, the azimuthal functions are expressed in terms of $F_m^\pm$, $G_m^\pm$, and $K_m^\pm$.

$$\begin{cases} {}^{<}P_\theta^{O,TE} = \tfrac{1}{4}\dfrac{m}{\bar{\rho}}\left(\left|G_m^\pm\right|^2 + \dfrac{m^2}{\bar{\rho}^2} + 1\right)\left|F_m^\pm\right|^2 \\ {}^{<}P_\theta^{O,TM} = N^2\,{}^{<}P_\theta^{O,TE} \\ {}^{>}P_\theta^{O,TE} = -{}^{<}P_\theta^{O,TE} \\ {}^{<}P_\theta^{O,TM} = N^2\,{}^{<}P_\theta^{O,TE} \end{cases} . \qquad \text{(S8.27)}$$

The combined radial component of the orbital FD is given for the co-rotating and counter-rotational cases by

$$
\begin{aligned}
{}^{<<}P_\theta^O &= \frac{1}{4}\frac{N^2+|q|^2}{1+|q|^2}\frac{m}{\bar\rho}\left(\left|G_m^\pm\right|^2+\frac{m^2}{\bar\rho^2}+1\right)\left|F_m^\pm\right|^2 \\
{}^{><}P_\theta^O &= \frac{1}{4}\frac{N^2-|q|^2}{1+|q|^2}\frac{m}{\bar\rho}\left(\left|G_m^\pm\right|^2+\frac{m^2}{\bar\rho^2}+1\right)\left|F_m^\pm\right|^2
\end{aligned}
. \qquad \text{(S8.28)}
$$

For the co-rotational case, it is interesting that ${}^{<<}P_\theta^O = (m/\bar\rho)w$. In words, the azimuthal component of the orbital FD is $(m/\bar\rho)$-times the energy density w, the latter being defined in Eq. (S2.3). In addition, there is a possibility of a vanishing azimuthal component for the counter-rotational case if $N^2 = |q|^2$.

As with the trajectories for the Poynting vectors, we can find the trajectories for the orbital FD. Consider a trajectory formed by the orbital FD.

$$
\begin{aligned}
\frac{d\theta}{d\bar\rho} &= \frac{1}{\bar\rho}\frac{P_\theta^O}{P_r^O} = \frac{1}{\bar\rho}\frac{\frac{1}{4}\frac{N^2\pm|q|^2}{1+|q|^2}\frac{m}{\bar\rho}\left(\left|G_m^\pm\right|^2+\frac{m^2}{\bar\rho^2}+1\right)\left|F_m^\pm\right|^2}{\frac{1}{4}\frac{N^2+|q|^2}{1+|q|^2}\left|F_m^\pm\right|^2 \operatorname{Im}\left[\left|G_m^\pm\right|^2 K_m^\pm + \left(1+\frac{m^2}{\bar\rho^2}\right)G_m^\pm - \frac{m^2}{\bar\rho^3}\right]} \\
&= \frac{m}{\bar\rho^2}\frac{N^2\pm|q|^2}{N^2+|q|^2}\frac{\left|G_m^\pm\right|^2+\frac{m^2}{\bar\rho^2}+1}{\operatorname{Im}\left[\left|G_m^\pm\right|^2 K_m^\pm + \left(1+\frac{m^2}{\bar\rho^2}\right)G_m^\pm - \frac{m^2}{\bar\rho^3}\right]}
\end{aligned}
. \qquad \text{(S8.29)}
$$

As usual, the double signs $\pm$ refer to the co- and counter-rotational cases, respectively. We find that both trajectories do not depend on $\left|F_m^\pm\right|$, the function itself. Instead, they depend strongly on the logarithmic derivatives $G_m^\pm$ and $K_m^\pm$.

S9. Spin Vector

The spin vector is given by $\vec S \equiv \frac{1}{4}\operatorname{Im}\left(\mu^{-1}\vec E^* \times \vec E + \varepsilon^{-1}\vec H^* \times \vec H\right)$. For dielectric and non-

magnetic media,

$$\vec{S} \equiv \tfrac{1}{4}\operatorname{Im}\left(\mu^{-1}\vec{E}^* \times \vec{E} + \varepsilon^{-1}\vec{H}^* \times \vec{H}\right)$$
$$= \frac{1}{(2n)^2}\operatorname{Im}\left(\varepsilon\vec{E}^* \times \vec{E} + \mu\vec{H}^* \times \vec{H}\right) = \frac{1}{(2n)^2}\operatorname{Im}\left(n^2\vec{E}^* \times \vec{E} + \vec{H}^* \times \vec{H}\right). \quad \text{(S9.1)}$$

From the appearance of Eq. (S9.1), we may call $\vec{E}^* \times \vec{E}$ and $\vec{H}^* \times \vec{H}$ "intra-electric-field outer product" and "intra-magnetic-field outer product, respectively.

Consider first the co-rotational case according to Eq. (S1.2). For convenience, let us introduce

$$\vec{S} \equiv \frac{1}{4}\frac{1}{1+|q|^2}\operatorname{Im}\left(\vec{S}_0\right). \quad \text{(S9.2)}$$

It is thus convenient to examine $\vec{S}_0$ first.

$$\begin{aligned}\vec{S}_0 &= \left(f_r^* e^{-im\theta}, f_\theta^* e^{-im\theta}, q^* f_z^* e^{-im\theta}\right) \times \left(f_r e^{im\theta}, f_\theta e^{im\theta}, q f_z e^{im\theta}\right)\\ &+\left(q^* h_r^* e^{-im\theta}, q^* h_\theta^* e^{-im\theta}, h_z^* e^{-im\theta}\right) \times \left(q h_r e^{im\theta}, q h_\theta e^{im\theta}, h_z e^{im\theta}\right)\\ &=\left(f_\theta^* q f_z - q^* f_z^* f_\theta\right)\hat{e}_r + \left(q^* f_z^* f_r - f_r^* q f_z\right)\hat{e}_\theta + \left(f_r^* f_\theta - f_\theta^* f_r\right)\hat{e}_z\\ &+\left(q^* h_\theta^* h_z - h_z^* q h_\theta\right)\hat{e}_r + \left(h_z^* q h_r - q^* h_r^* h_z\right)\hat{e}_\theta + \left(q^* h_r^* q h_\theta - q^* h_\theta^* q h_r\right)\hat{e}_z\end{aligned} . \quad \text{(S9.3)}$$

We note that both $e^{im\theta}$ and $e^{-im\theta}$ cancel each other in every terms. We substitute Eq. (S1.3) into (S9.3) to obtain

$$\begin{aligned}\vec{S}_0 &= \left(qi\frac{dh_z^*}{d\bar{\rho}} f_z + iq^* f_z^* \frac{dh_z}{d\bar{\rho}}\right)\hat{e}_r + \left(-q^* f_z^* \frac{m}{\bar{\rho}} h_z + \frac{m}{\bar{\rho}} h_z^* q f_z\right)\hat{e}_\theta\\ &+\left(-\frac{m}{\bar{\rho}} h_z^* (-i)\frac{dh_z}{d\bar{\rho}} - i\frac{dh_z^*}{d\bar{\rho}}\frac{-m}{\bar{\rho}} h_z\right)\hat{e}_z + \left(-q^* i\frac{df_z^*}{d\bar{\rho}} h_z - q h_z^* i\frac{df_z}{d\bar{\rho}}\right)\hat{e}_r\\ &+\left(q h_z^* \frac{m}{\bar{\rho}} f_z - q^* \frac{m}{\bar{\rho}} f_z^* h_z\right)\hat{e}_\theta + |q|^2\left(\frac{m}{\bar{\rho}} f_z^* i\frac{df_z}{d\bar{\rho}} + i\frac{df_z^*}{d\bar{\rho}}\frac{m}{\bar{\rho}} f_z\right)\hat{e}_z\end{aligned} . \quad \text{(S9.4)}$$

Taking its imaginary part, we find

$$\begin{aligned}\operatorname{Im}\left(\vec{S}_0\right) &= \operatorname{Im}\left(qi\frac{dh_z^*}{d\bar{\rho}}f_z + iq^* f_z^* \frac{dh_z}{d\bar{\rho}}\right)\hat{e}_r + \operatorname{Im}\left(-q^* f_z^* \frac{m}{\bar{\rho}}h_z + \frac{m}{\bar{\rho}}h_z^* q f_z\right)\hat{e}_\theta \\ &+\operatorname{Im}\left(-\frac{m}{\bar{\rho}}h_z^*(-i)\frac{dh_z}{d\bar{\rho}} - i\frac{dh_z^*}{d\bar{\rho}}\frac{-m}{\bar{\rho}}h_z\right)\hat{e}_z \\ &+\operatorname{Im}\left(-q^* i\frac{df_z^*}{d\bar{\rho}}h_z - qh_z^* i\frac{df_z}{d\bar{\rho}}\right)\hat{e}_r + \operatorname{Im}\left(qh_z^*\frac{m}{\bar{\rho}}f_z - q^*\frac{m}{\bar{\rho}}f_z^* h_z\right)\hat{e}_\theta \\ &+|q|^2\operatorname{Im}\left(\frac{m}{\bar{\rho}}f_z^* i\frac{df_z}{d\bar{\rho}} + i\frac{df_z^*}{d\bar{\rho}}\frac{m}{\bar{\rho}}f_z\right)\hat{e}_z\end{aligned} \quad . \qquad \text{(S9.5)}$$

$$\begin{aligned}\operatorname{Im}\left(\vec{S}_0\right) &= 2\operatorname{Im}\left(qi\frac{dh_z^*}{d\bar{\rho}}f_z\right)\hat{e}_r + 2\frac{m}{\bar{\rho}}\operatorname{Im}\left(qh_z^* f_z\right)\hat{e}_\theta + 2\frac{m}{\bar{\rho}}\operatorname{Im}\left(ih_z^*\frac{dh_z}{d\bar{\rho}}\right)\hat{e}_z \\ &-2\operatorname{Im}\left(qi\frac{df_z}{d\bar{\rho}}h_z^*\right)\hat{e}_r + 2\frac{m}{\bar{\rho}}\operatorname{Im}\left(qh_z^* f_z\right)\hat{e}_\theta + 2\frac{m}{\bar{\rho}}|q|^2\operatorname{Im}\left(if_z^*\frac{df_z}{d\bar{\rho}}\right)\hat{e}_z\end{aligned} \quad . \qquad \text{(S9.6)}$$

Here, we have employed the fact that $\operatorname{Im}\left(A - A^*\right) = 2\operatorname{Im}\left(A\right)$ for a genetic complex variable A. Besides, we have tried to keep q rather than q^* in all the terms for the convenience in the ensuing manipulations. We now employ the fact that $\operatorname{Im}\left(iA\right) = \operatorname{Re}\left(A\right)$ for a genetic complex variable A. Eq. (S9.6) then simplifies further to

$$\begin{aligned}\tfrac{1}{2}\operatorname{Im}\left(\vec{S}_0\right) &= \operatorname{Re}\left(q\frac{dh_z^*}{d\bar{\rho}}f_z\right)\hat{e}_r + \frac{m}{\bar{\rho}}\operatorname{Im}\left(qh_z^* f_z\right)\hat{e}_\theta + \frac{m}{\bar{\rho}}\operatorname{Re}\left(h_z^*\frac{dh_z}{d\bar{\rho}}\right)\hat{e}_z \\ &-\operatorname{Re}\left(q\frac{df_z}{d\bar{\rho}}h_z^*\right)\hat{e}_r + \frac{m}{\bar{\rho}}\operatorname{Im}\left(qh_z^* f_z\right)\hat{e}_\theta + \frac{m}{\bar{\rho}}|q|^2\operatorname{Re}\left(f_z^*\frac{df_z}{d\bar{\rho}}\right)\hat{e}_z\end{aligned} \quad . \qquad \text{(S9.7)}$$

Therefore, from $\vec{S} = \tfrac{1}{4}\left(1+|q|^2\right)^{-1}\operatorname{Im}\left(\vec{S}_0\right)$ in Eq. (S9.2), we obtain

$$\begin{aligned}\vec{S} &= \frac{1}{4}\frac{1}{1+|q|^2}\operatorname{Im}\left(\vec{S}_0\right) = \frac{1}{2}\frac{1}{1+|q|^2}\operatorname{Re}\left[q\left(\frac{dh_z^*}{d\bar{\rho}}f_z - \frac{df_z}{d\bar{\rho}}h_z^*\right)\right]\hat{e}_r \\ &+\frac{1}{1+|q|^2}\frac{m}{\bar{\rho}}\operatorname{Im}\left(qh_z^* f_z\right)\hat{e}_\theta + \frac{1}{2}\frac{1}{1+|q|^2}\frac{m}{\bar{\rho}}\operatorname{Re}\left(h_z^*\frac{dh_z}{d\bar{\rho}} + |q|^2 f_z^*\frac{df_z}{d\bar{\rho}}\right)\hat{e}_z\end{aligned} \quad . \qquad \text{(S9.8)}$$

Expressed in terms of $F_m^{\pm}$ in Eq. (S1.6) and $G_m^{\pm}$ in Eq. (S1.7), the above becomes

$$\begin{aligned}
\vec{S} &= \frac{1}{2}\frac{N}{1+|q|^2}\mathrm{Re}\left[q\left(\frac{dF_m^{\pm,*}}{d\bar{\rho}}F_m^{\pm} - \frac{dF_m^{\pm}}{d\bar{\rho}}F_m^{\pm,*}\right)\right]\hat{e}_r \\
&+\frac{N}{1+|q|^2}\frac{m}{\bar{\rho}}\mathrm{Im}\left(qF_m^{\pm,*}F_m^{\pm}\right)\hat{e}_\theta + \frac{1}{2}\frac{N^2+|q|^2}{1+|q|^2}\frac{m}{\bar{\rho}}\mathrm{Re}\left(F_m^{\pm,*}\frac{dF_m^{\pm}}{d\bar{\rho}}\right)\hat{e}_z \\
&=\frac{1}{2}\frac{N}{1+|q|^2}\mathrm{Re}\left(-iq\right)2\,\mathrm{Im}\left(\frac{dF_m^{\pm}}{d\bar{\rho}}F_m^{\pm,*}\right)\hat{e}_r \\
&+\frac{N}{1+|q|^2}\frac{m}{\bar{\rho}}\left|F_m^{\pm}\right|^2\mathrm{Im}\left(q\right)\hat{e}_\theta + \frac{1}{2}\frac{N^2+|q|^2}{1+|q|^2}\frac{m}{\bar{\rho}}\left|F_m^{\pm}\right|^2\mathrm{Re}\left(G_m^{\pm}\right)\hat{e}_z \\
&=\frac{N}{1+|q|^2}\left|F_m^{\pm}\right|^2\mathrm{Im}\left(q\right)\mathrm{Im}\left(G_m^{\pm}\right)\hat{e}_r \\
&+\frac{N}{1+|q|^2}\frac{m}{\bar{\rho}}\left|F_m^{\pm}\right|^2\mathrm{Im}\left(q\right)\hat{e}_\theta + \frac{1}{2}\frac{N^2+|q|^2}{1+|q|^2}\frac{m}{\bar{\rho}}\left|F_m^{\pm}\right|^2\mathrm{Re}\left(G_m^{\pm}\right)\hat{e}_z
\end{aligned} \quad . \qquad \text{(S9.9)}$$

Here, we utilized the following fact for a genetic complex variable A and q.

$$\begin{cases} A^* - A = \mathrm{Re}(A) - i\,\mathrm{Im}(A) - \mathrm{Re}(A) - i\,\mathrm{Im}(A) = -2i\,\mathrm{Im}(A) \\ \mathrm{Re}(-iq) = \mathrm{Re}\left[-i\,\mathrm{Re}(q) + \mathrm{Im}(q)\right] = \mathrm{Im}(q) \end{cases} . \qquad \text{(S9.10)}$$

Finally, Eq. (S9.9) is reduced to

$$\begin{cases} S_r = \sigma\frac{1}{2}N\left|F_m^{\pm}\right|^2\mathrm{Im}\left(G_m^{\pm}\right) \\ S_\theta = \sigma\frac{1}{2}N\frac{m}{\bar{\rho}}\left|F_m^{\pm}\right|^2 \\ S_z = \frac{1}{2}\frac{N^2+|q|^2}{1+|q|^2}\frac{m}{\bar{\rho}}\left|F_m^{\pm}\right|^2\mathrm{Re}\left(G_m^{\pm}\right) \end{cases} . \qquad \text{(S9.11)}$$

We notice that the helicity-dependent in-plane components $\left(S_r, S_\theta\right)$ are not separable into the TM and TE modes due to non-rectilinear motions, whereas the axial spin component S_z

is separable. In the interior, we have simply $S_r = 0$. In the exterior, not only $\sigma \neq 0$ or $\mathrm{Im}(q) \neq 0$ but also the spatial inhomogeneity of $F_m^{\pm}(\rho)$ are required for $S_r \neq 0$ in addition to the non-zero factor $\mathrm{Im}(G_m^{\pm})$. By the same token, $S_\theta \neq 0$ for $m \neq 0$ in Eq. (21), which refers to an azimuthal inhomogeneity.

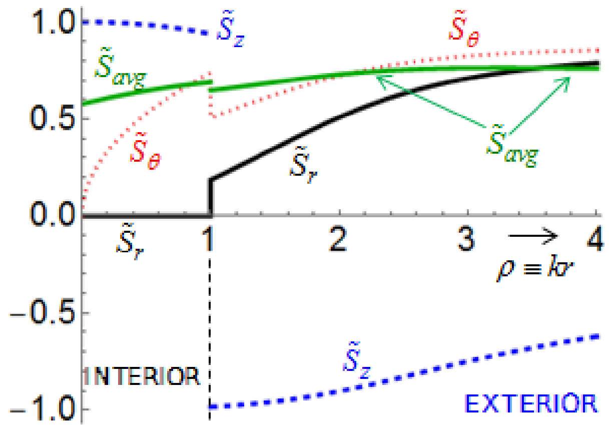

Figure S9.1: Spin components $(\tilde{S}_r, \tilde{S}_\theta, \tilde{S}_z)$ for the co-rotational case plotted over $0 \leq \rho \equiv kr \leq 4$ for $m = 3$, $R_\rho \equiv kR = 1$, and $q = (1+i)/\sqrt{2}$.

We define the normalized spin $\vec{S}/w$ per photon. For a better viewing, we introduce scaling, for instance, such that $\tilde{S}_r \equiv \mathrm{sgn}(S_r)|S_r/w|^{1/4}$. Figure S9.1 presents such $(\tilde{S}_r, \tilde{S}_\theta, \tilde{S}_z)$ over $0 \leq \rho \equiv kr \leq 4$ with $R_\rho \equiv kR = 1$ and $m = 3$. In particular, we provided a non-trivial coupling $q = (1+i)/\sqrt{2}$. Furthermore, we find through numerical computations that $|S_\theta| < w$ and $|S_z| < w$ as well. The average spin $S_{avg} \equiv \sqrt{\frac{1}{3}(S_r^2 + S_\theta^2 + S_z^2)} < w$ is plotted in green curve. All the curves in Figure S9.1 display discontinuities in the radial profiles across the thin layer. First, $S_z(\rho) > 0$ in the interior, whereas $S_z(\rho) < 0$ in the exterior. This spin flips take place due to the thin layer, where excitations are supplied. Second, $S_\theta(\rho) > 0$ over all $\rho \equiv kr$.

In addition, we find from Eq. (S9.11) the ratio of the in-plane components is found to be $S_r/S_\theta = m^{-1}\bar{\rho}\,\mathrm{Im}(G_m^{\pm})$. As a result, photon spins form circular streamlines in the interior or

$S_r/S_\theta = 0$ because $\mathrm{Im}\left(G_m^-\right) = 0$ according to Fig. S1.1. In comparison, $S_r/S_\theta > 0$, because $\mathrm{Im}\left(G_m^+\right) > 0$ according again to Fig. S1.1. Hence, the spin trajectories in the exterior are directed radially outward and they point to the counter-clockwise direction.

Let us turn to the counter-rotational case via Eq. (S1.4).

$$\begin{aligned}
\vec{S}_0 &= \left(f_r^* e^{-im\theta}, f_\theta^* e^{-im\theta}, q^* f_z^* e^{im\theta} \right) \times \left(f_r e^{im\theta}, f_\theta e^{im\theta}, q f_z e^{-im\theta} \right) \\
&+ \left(q^* h_r^* e^{im\theta}, q^* h_\theta^* e^{im\theta}, h_z^* e^{-im\theta} \right) \times \left(q h_r e^{-im\theta}, q h_\theta e^{-im\theta}, h_z e^{im\theta} \right) \\
&= \left(f_\theta^* q f_z e^{-2im\theta} - q^* f_z^* f_\theta e^{2im\theta} \right) \hat{e}_r + \left(q^* f_z^* f_r e^{2im\theta} - f_r^* q f_z e^{-2im\theta} \right) \hat{e}_\theta \\
&+ \left(f_r^* f_\theta - f_\theta^* f_r \right) \hat{e}_z \\
&+ \left(q^* h_\theta^* h_z e^{2im\theta} - h_z^* q h_\theta e^{-2im\theta} \right) \hat{e}_r + \left(h_z^* q h_r e^{-2im\theta} - q^* h_r^* h_z e^{2im\theta} \right) \hat{e}_\theta \\
&+ \left(q^* h_r^* q h_\theta - q^* h_\theta^* q h_r \right) \hat{e}_z
\end{aligned} \quad . \qquad \text{(S9.12)}$$

We note that both $e^{im\theta}$ and $e^{-im\theta}$ collaborate for the in-plane components so that squared azimuthal factor $\exp\left(\pm 2im\theta\right)$ continues to appear. However, they cancel each other in the axial terms. As a result, Eq. (S9.12) is quite different from its counterpart in Eq. (S9.3) for the co-rotational case.

To our great surprise, the following procedure leads us to an entirely radical result. To see this fact, let us substitute Eq. (S1.5) into (S9.12) to obtain

$$\begin{aligned}
\vec{S}_0 &= \left(qi \frac{dh_z^*}{d\bar{\rho}} f_z e^{-2im\theta} + iq^* f_z^* \frac{dh_z}{d\bar{\rho}} e^{2im\theta} \right) \hat{e}_r \\
&+ \left(-q^* f_z^* \frac{m}{\bar{\rho}} h_z e^{2im\theta} + \frac{m}{\bar{\rho}} h_z^* q f_z e^{-2im\theta} \right) \hat{e}_\theta + \left(-\frac{m}{\bar{\rho}} h_z^* (-i) \frac{dh_z}{d\bar{\rho}} - i \frac{dh_z^*}{d\bar{\rho}} \frac{-m}{\bar{\rho}} h_z \right) \hat{e}_z \\
&+ \left(-q^* i \frac{df_z^*}{d\bar{\rho}} h_z e^{2im\theta} - q h_z^* i \frac{df_z}{d\bar{\rho}} e^{-2im\theta} \right) \hat{e}_r \\
&+ \left(q h_z^* \frac{-m}{\bar{\rho}} f_z e^{-2im\theta} + q^* \frac{m}{\bar{\rho}} f_z^* h_z e^{2im\theta} \right) \hat{e}_\theta + |q|^2 \left(-\frac{m}{\bar{\rho}} f_z^* i \frac{df_z}{d\bar{\rho}} + i \frac{df_z^*}{d\bar{\rho}} \frac{-m}{\bar{\rho}} f_z \right) \hat{e}_z
\end{aligned} \quad . \qquad \text{(S9.13)}$$

Therefore, taking the imaginary part leads to

$$
\begin{aligned}
\mathrm{Im}\left(\vec{S}_0\right) &= \mathrm{Im}\left(qi\frac{dh_z^*}{d\bar{\rho}} f_z e^{-2im\theta} + iq^* f_z^* \frac{dh_z}{d\bar{\rho}} e^{2im\theta} \right)\hat{e}_r \\
&+ \frac{m}{\bar{\rho}}\mathrm{Im}\left(qh_z^* f_z e^{-2im\theta} - q^* h_z f_z^* e^{2im\theta} \right)\hat{e}_\theta + \frac{m}{\bar{\rho}}\mathrm{Im}\left(ih_z^* \frac{dh_z}{d\bar{\rho}} + i\frac{dh_z^*}{d\bar{\rho}} h_z \right)\hat{e}_z \\
&- \mathrm{Im}\left(qh_z^* i\frac{df_z}{d\bar{\rho}} e^{-2im\theta} + q^* i\frac{df_z^*}{d\bar{\rho}} h_z e^{2im\theta} \right)\hat{e}_r \\
&- \frac{m}{\bar{\rho}}\mathrm{Im}\left(qh_z^* f_z e^{-2im\theta} - q^* h_z f_z^* e^{2im\theta} \right)\hat{e}_\theta - |q|^2 \frac{m}{\bar{\rho}}\mathrm{Im}\left(if_z^* \frac{df_z}{d\bar{\rho}} + i\frac{df_z^*}{d\bar{\rho}} f_z \right)\hat{e}_z
\end{aligned} . \qquad \text{(S9.14)}
$$

If further processed, it gets to

$$
\begin{aligned}
\mathrm{Im}\left(\vec{S}_0\right) &= 2\,\mathrm{Im}\left(iq\frac{dh_z^*}{d\bar{\rho}} f_z e^{-2im\theta} \right)\hat{e}_r + 2\frac{m}{\bar{\rho}}\mathrm{Im}\left(qh_z^* f_z e^{-2im\theta} \right)\hat{e}_\theta \\
&+ \frac{m}{\bar{\rho}}\mathrm{Im}\left(ih_z^* \frac{dh_z}{d\bar{\rho}} + i\frac{dh_z^*}{d\bar{\rho}} h_z \right)\hat{e}_z \\
&-2\,\mathrm{Im}\left(iqh_z^* \frac{df_z}{d\bar{\rho}} e^{-2im\theta} \right)\hat{e}_r - 2\frac{m}{\bar{\rho}}\mathrm{Im}\left(qh_z^* f_z e^{-2im\theta} \right)\hat{e}_\theta \\
&- \frac{m}{\bar{\rho}}|q|^2\,\mathrm{Im}\left(if_z^* \frac{df_z}{d\bar{\rho}} + i\frac{df_z^*}{d\bar{\rho}} f_z \right)\hat{e}_z
\end{aligned} . \qquad \text{(S9.15)}
$$

Here, we have employed the fact that $\mathrm{Im}\left(A - A^*\right) = 2\,\mathrm{Im}\left(A\right)$ for a genetic complex variable A. Curiously enough, we find that the two terms of Eq. (S9.15) in the azimuthal direction cancel each other (as marked in red colors). The minute step involving this cancellation in the azimuthal direction will lead to the bizarre result as mentioned before.

Via $\mathrm{Im}\left(iA\right) = \mathrm{Re}\left(A\right)$ and $\mathrm{Re}\left(A\right) = \mathrm{Re}\left(A^*\right)$ for a genetic complex variable A, Eq. (S9.15) further simplifies to

$$\tfrac{1}{2}\mathrm{Im}\left(\vec{S}_0\right)=\mathrm{Re}\left(q\frac{dh_z^*}{d\bar{\rho}}f_z e^{-2im\theta}\right)\hat{e}_r+\frac{m}{\bar{\rho}}\mathrm{Re}\left(h_z^*\frac{dh_z}{d\bar{\rho}}\right)\hat{e}_z$$
$$-\mathrm{Re}\left(qh_z^*\frac{df_z}{d\bar{\rho}}e^{-2im\theta}\right)\hat{e}_r-\frac{m}{\bar{\rho}}|q|^2\,\mathrm{Re}\left(f_z^*\frac{df_z}{d\bar{\rho}}\right)\hat{e}_z \qquad . \qquad \text{(S9.16)}$$
$$=\mathrm{Re}\left[qe^{-2im\theta}\left(\frac{dh_z^*}{d\bar{\rho}}f_z-\frac{df_z}{d\bar{\rho}}h_z^*\right)\right]\hat{e}_r+\frac{m}{\bar{\rho}}\mathrm{Re}\left(h_z^*\frac{dh_z}{d\bar{\rho}}-|q|^2 f_z^*\frac{df_z}{d\bar{\rho}}\right)\hat{e}_z$$

By $\vec{S}=\tfrac{1}{4}\left(1+|q|^2\right)^{-1}\mathrm{Im}\left(\vec{S}_0\right)$ in Eq. (S9.2), we go back to the spin vector to have

$$\vec{S}=\frac{1}{4}\frac{1}{1+|q|^2}\mathrm{Im}\left(\vec{S}_0\right)=\frac{1}{2}\frac{1}{1+|q|^2}\mathrm{Re}\left[qe^{-2im\theta}\left(\frac{dh_z^*}{d\bar{\rho}}f_z-\frac{df_z}{d\bar{\rho}}h_z^*\right)\right]\hat{e}_r$$
$$+\frac{1}{2}\frac{1}{1+|q|^2}\frac{m}{\bar{\rho}}\mathrm{Re}\left(h_z^*\frac{dh_z}{d\bar{\rho}}-|q|^2 f_z^*\frac{df_z}{d\bar{\rho}}\right)\hat{e}_z \qquad . \qquad \text{(S9.17)}$$

Expressed in terms of $F_m^{\pm}$ in Eq. (S1.6) and $G_m^{\pm}$ in Eq. (S1.7), Eq. (S9.17) is further simplified to

$$\vec{S}=\frac{1}{2}\frac{N}{1+|q|^2}\mathrm{Re}\left[qe^{-2im\theta}\left(\frac{dF_m^{\pm,*}}{d\bar{\rho}}F_m^{\pm}-\frac{dF_m^{\pm}}{d\bar{\rho}}F_m^{\pm,*}\right)\right]\hat{e}_r$$
$$+\frac{1}{2}\frac{N^2-|q|^2}{1+|q|^2}\frac{m}{\bar{\rho}}\mathrm{Re}\left(F_m^{\pm,*}\frac{dF_m^{\pm}}{d\bar{\rho}}\right)\hat{e}_z$$
$$=\frac{1}{2}\frac{N}{1+|q|^2}\mathrm{Re}\left(-iqe^{-2im\theta}\right)2\,\mathrm{Im}\left(\frac{dF_m^{\pm}}{d\bar{\rho}}F_m^{\pm,*}\right)\hat{e}_r+\frac{1}{2}\frac{N^2-|q|^2}{1+|q|^2}\frac{m}{\bar{\rho}}\left|F_m^{\pm}\right|^2\mathrm{Re}\left(G_m^{\pm}\right)\hat{e}_z \qquad . \qquad \text{(S9.18)}$$
$$=\frac{N}{1+|q|^2}\left|F_m^{\pm}\right|^2\mathrm{Im}\left(qe^{-2im\theta}\right)\mathrm{Im}\left(G_m^{\pm}\right)\hat{e}_r+\frac{1}{2}\frac{N^2-|q|^2}{1+|q|^2}\frac{m}{\bar{\rho}}\left|F_m^{\pm}\right|^2\mathrm{Re}\left(G_m^{\pm}\right)\hat{e}_z$$

Here, we utilized Eq. (S9.10) for a genetic complex variable A and q. Therefore, for the counter-rotational case, we obtain the following with the chirality χ defined in Eq. (S1.11).

$$\begin{cases} S_r = \chi \frac{1}{2} N \left| F_m^{\pm} \right|^2 \mathrm{Im}\left(G_m^{\pm} \right) \\ S_\theta = 0 \\ S_z = \frac{1}{2} \frac{N^2 - |q|^2}{1 + |q|^2} \frac{m}{\rho} \left| F_m^{\pm} \right|^2 \mathrm{Re}\left(G_m^{\pm} \right) \end{cases} . \tag{S9.19}$$

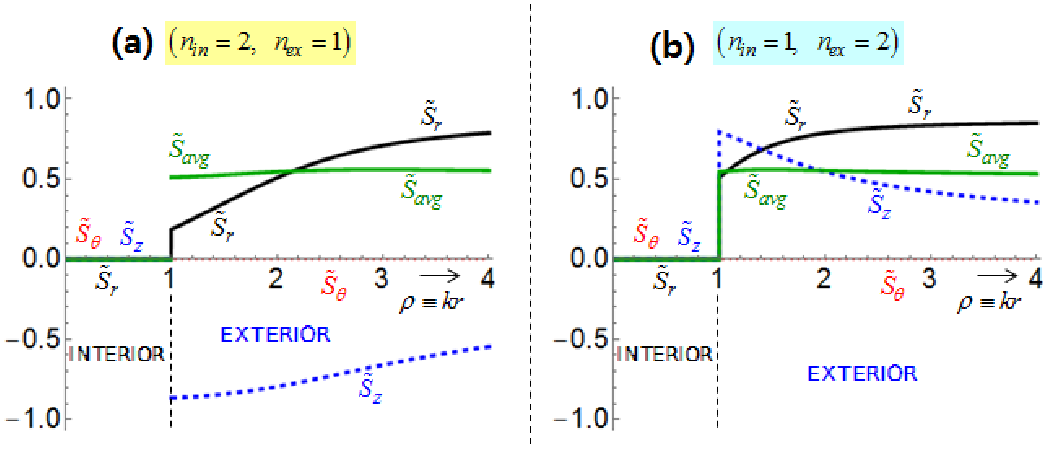

Figure S9.2: Spin components $\left(\tilde{S}_r, \tilde{S}_\theta, \tilde{S}_z \right)$ for the counter-rotational case plotted over $0 \le \rho \equiv kr \le 4$ for $m = 3$, $R_\rho \equiv kR = 1$, and $q = (1+i)/\sqrt{2}$.

Figure S9.2 presents such $\left(\tilde{S}_r, \tilde{S}_\theta, \tilde{S}_z \right)$ over $0 \le \rho \equiv kr \le 4$ with $R_\rho \equiv kR = 1$, $m = 3$, and $q = (1+i)/\sqrt{2}$. Notice hence that $|q| = 1$. Furthermore, we set $\exp(-2im\theta) = 1$ in Eq. (S1.11) for convenience so that $\sigma = \chi$. The refractive indices are $n^- = 2$ (say, glass) and $n^+ = 1$ (say, air) for panel (a), whereas they are reversed such that $n^- = 1$ (say, air) and $n^+ = 2$ (say, glass) for panel (b). We find through numerical computations that $|S_r|, |S_\theta|, |S_z| < w$. The average spin $S_{avg} \equiv \sqrt{\frac{1}{3}\left(S_r^2 + S_\theta^2 + S_z^2 \right)} < w$ is plotted in green curve. All the curves in Figure S9.2 display discontinuities in the radial profiles across the thin layer. However, S_r shows an approximately similar behavior on both panels.

On panel (a) of Figure S9.2, it happen on panel (a) that $S_z(\rho)=0$ in the interior from Eq. (S9.19) for the counter-rotational case, because $N=1$ in interior. In comparison, $S_z(\rho)<0$ in the exterior. On panel (b), $S_z(\rho)=0$ again in the interior, because $N=1$ still in interior. Only difference is that $N=2$ and $N=\frac{1}{2}$ in the exterior for panels (a) and (b), respectively. In evaluating S_z in Eq. (S9.19), $N^2-|q|^2=4-1=3>0$ for panel (a) and $N^2-|q|^2=\frac{1}{4}-1=-\frac{3}{4}<0$ for panel (b), respectively. As a result, $S_z<0$ on panel (a) and $S_z>0$ on panel (b), respectively.

Let us compare Eq. (S9.11) to Eq. (S9.19) for the three components of the co- and counter-rotational cases. For instance, S_r undergoes a little change in the multiplying factor from σ for the co-rotational case to χ for the counter-rotational case. Notice that χ depends strongly on the azimuthal angle as is the optical chiralty C presented in Eq. (S3.9). We can easily find that the usual sign changes are encountered for S_z.

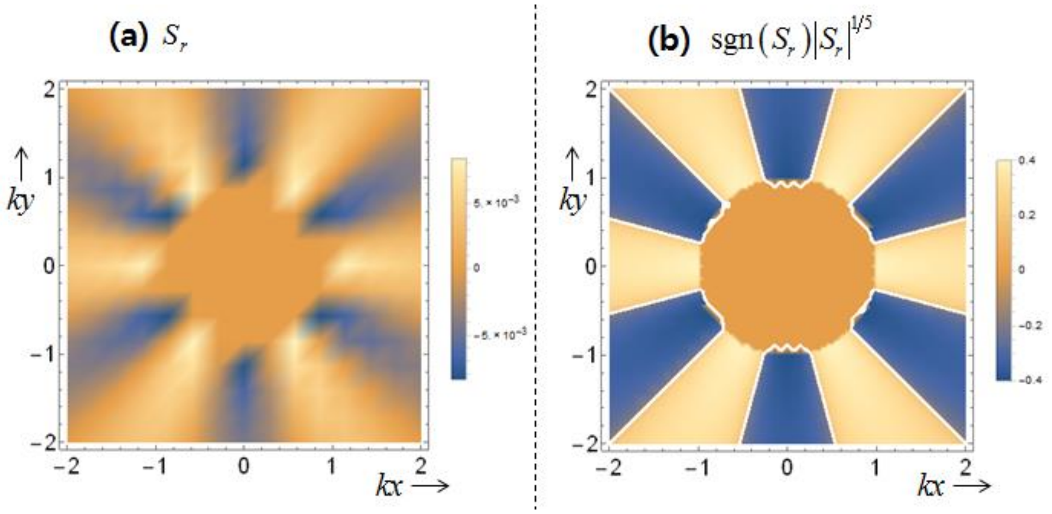

Figure S9.3: (a) A contour plot of the radial component of spin S_r. (b) A contour plot of its scaled value $\mathrm{sgn}(S_r)|S_r|^{1/5}$. Both are plotted on the (kx,ky)-plane for the counter-rotational case. The common data are $R_\rho \equiv kR=1$, $m=3$, and $q=i$.

Figure S9.3(a) exhibits a contour plot of the radial component of spin S_r, whereas panel (b) shows a contour plot of its scaled value $\mathrm{sgn}(S_r)|S_r|^{1/5}$. Both are plotted on the (kx,ky)-plane for the counter-rotational case. The common data are $R_\rho \equiv kR = 1$, $m=3$, and $q=i$. We find that S_r on panel (a) exhibits a blurred image so that its spatial distribution is hard to reveal itself.

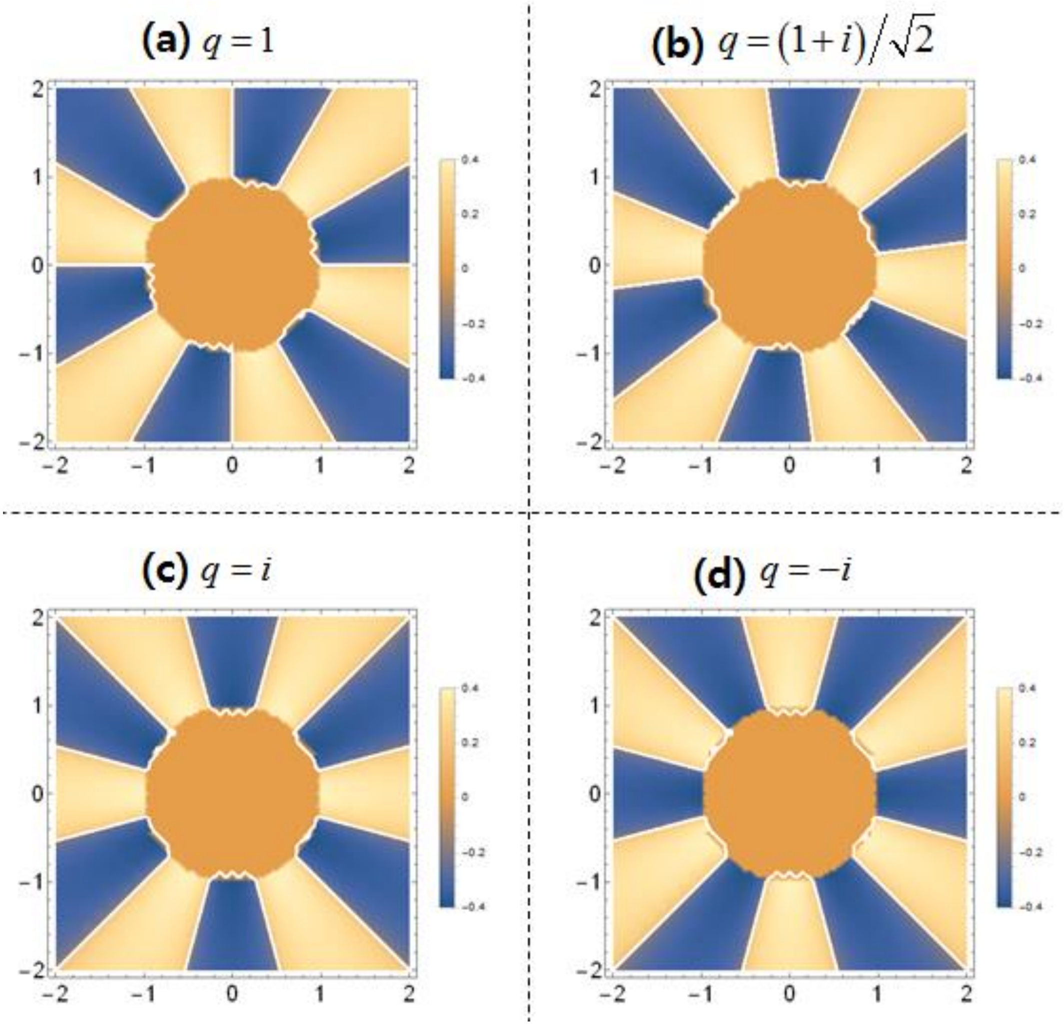

Figure S9.4: Contour plots of $\mathrm{sgn}(S_r)|S_r|^{1/5}$ on the (kx,ky)-plane for the counter-rotational case. The common data are $R_\rho \equiv kR = 1$ and $m=3$. Besides, $q=1,(1+i)/\sqrt{2},i,-i$ as written on each panel.

Figure S9.4 shows contour plots of the scaled value $\operatorname{sgn}(S_r)|S_r|^{1/5}$ on the (kx,ky)-plane for the counter-rotational case. The common data are $R_\rho \equiv kR = 1$ and $m=3$. The TE-TM coupling coefficient is varied such that $q = 1,(1+i)/\sqrt{2},i,-i$ as written on each panel. Hence, panel (c) is what we have already presented. Figure S9.4 is therefore shows the effect of the factor $\operatorname{Im}\left(qe^{-2im\theta}\right)$ on S_r.

It is $S_\theta = 0$ for the counter-rotational case, which is radically different from $S_\theta = \frac{1}{2}\sigma N(m/\bar{\rho})\left|F_m^\pm\right|^2$ in Eq. (S9.11) for the co-rotational case. Note from Eq. (S9.15) that the trivial identity $S_\theta = 0$ stems from the cancellation between the electric- and magnetic-field contributions during counter-rotations of the TE and TM waves. To take a closer look at this fact, consider the azimuthal part of Eq. (S9.12)

$$\begin{aligned}\vec{S}_{0,\theta} &\equiv \left(q^* f_z^* f_r e^{2im\theta} - f_r^* q f_z e^{-2im\theta}\right) + \left(h_z^* q h_r e^{-2im\theta} - q^* h_r^* h_z e^{2im\theta}\right) \\ &= \left(qe^{-2im\theta} f_r^* f_z\right)^* - \left(qe^{-2im\theta} f_r^* f_z\right) + \left(qe^{-2im\theta} h_z^* h_r\right) - \left(qe^{-2im\theta} h_z^* h_r\right)^*.\end{aligned} \tag{S9.20}$$

Taking its imaginary parts,

$$\begin{aligned}\operatorname{Im}\left(\vec{S}_{0,\theta}\right) &= \operatorname{Im}\left[\left(qe^{-2im\theta} f_r^* f_z\right)^*\right] - \operatorname{Im}\left(qe^{-2im\theta} f_r^* f_z\right) \\ &\quad + \operatorname{Im}\left(qe^{-2im\theta} h_z^* h_r\right) - \operatorname{Im}\left[\left(qe^{-2im\theta} h_z^* h_r\right)^*\right] \\ &= -2\operatorname{Im}\left(qe^{-2im\theta} f_r^* f_z\right) + 2\operatorname{Im}\left(qe^{-2im\theta} h_z^* h_r\right)\end{aligned}. \tag{S9.21}$$

Again from Eq. (S1.5), we obtain

$$\begin{aligned}\operatorname{Im}\left(\vec{S}_{0,\theta}\right) &= -2\operatorname{Im}\left(qe^{-2im\theta}\frac{-m}{\bar{\rho}} h_z^* f_z\right) + 2\operatorname{Im}\left(qe^{-2im\theta} h_z^* \frac{-m}{\bar{\rho}} f_z\right) \\ &= 2\frac{m}{\bar{\rho}}\left[\operatorname{Im}\left(qe^{-2im\theta} h_z^* f_z\right) - \operatorname{Im}\left(qe^{-2im\theta} h_z^* f_z\right)\right] = 0\end{aligned}. \tag{S9.22}$$

We find that we have never employed the particular solutions $F_m^{\pm}$ in Eq. (S1.6) and $G_m^{\pm}$ in Eq. (S1.7) in order to obtain the conclusion that $S_\theta = 0$ for the counter-rotational case. Therefore, $S_\theta = 0$ holds true for any concentric cylinders as long as the refractive index can be expressed by any piecewise-continuous functions.

S10. Spin Vector for Counter-Rotations with Differing Angular Speeds

In the preceding section, one kind of waves propagate according to $\exp\left[i\left(m\theta-\omega t\right)\right]$, whereas the other kind of waves propagate according to $\exp\left[i\left(-m\theta-\omega t\right)\right]$. Consequently, our particular pair of the TE and TM waves leads to $S_\theta = 0$, when they are counter-rotational. We then raise a question: "Would any orthogonal pair of two would lead to $S_\theta = 0$?".

In order to answer this question, let us consider the two waves in counter-rotations but with different angular speeds. To this goal, let us modify both Eqs. (S1.4) and (S1.5) as follows for the counter-rotational case.

$$\begin{cases} \dfrac{\vec{E}}{\sqrt{\mu}} = \dfrac{1}{\sqrt{1+|q|^2}}\left(f_r e^{im\theta}, f_\theta e^{im\theta}, q f_z e^{-il\theta}\right) \\ \dfrac{\vec{H}}{\sqrt{\varepsilon}} = \dfrac{1}{\sqrt{1+|q|^2}}\left(q h_r e^{-il\theta}, q h_\theta e^{-il\theta}, h_z e^{im\theta}\right) \end{cases}. \tag{S10.1}$$

$$\begin{cases} TE: \quad h_r = -\dfrac{l}{\bar{\rho}} f_z, \quad h_\theta = i\dfrac{df_z}{d\bar{\rho}}; \\ TM: \quad f_r = -\dfrac{m}{\bar{\rho}} h_z, \quad f_\theta = -i\dfrac{dh_z}{d\bar{\rho}} \end{cases}. \tag{S10.2}$$

Here, we assume that $l, m > 0$ being integers. Therefore, the TE waves follow $\exp\left[i\left(-l\theta-\omega t\right)\right]$, while the TM waves follow $\exp\left[i\left(m\theta-\omega t\right)\right]$. Namely,

$$\begin{cases} TE: \ \exp\left[i\left(-l\theta-\omega t\right)\right] \\ TM: \ \exp\left[i\left(m\theta-\omega t\right)\right] \end{cases}. \tag{S10.3}$$

In both equations, the items in red color indicate what have been changed in comparison to Eqs. (S1.4) and (S1.5). Consider

$$\begin{aligned}\vec{S}_0 &= \left(f_r^* e^{-im\theta}, f_\theta^* e^{-im\theta}, q^* f_z^* e^{il\theta} \right) \times \left(f_r e^{im\theta}, f_\theta e^{im\theta}, q f_z e^{-il\theta} \right) \\ &+ \left(q^* h_r^* e^{il\theta}, q^* h_\theta^* e^{il\theta}, h_z^* e^{-im\theta} \right) \times \left(q h_r e^{-im\theta}, q h_\theta e^{-il\theta}, h_z e^{im\theta} \right)\end{aligned}. \tag{S10.4}$$

Let us consider only the azimuthal component for simplicity.

$$\begin{aligned}\vec{S}_{0,\theta} &\equiv \left(q^* f_z^* f_r e^{i(m+l)\theta} - f_r^* q f_z e^{-i(m+l)\theta} \right) + \left(h_z^* q h_r e^{-i(m+l)\theta} - q^* h_r^* h_z e^{i(m+l)\theta} \right) \\ &= \left(q e^{-i(m+l)\theta} f_r^* f_z \right)^* - \left(q e^{-i(m+l)\theta} f_r^* f_z \right) + \left(q e^{-i(m+l)\theta} h_z^* h_r \right) - \left(q e^{-i(m+l)\theta} h_z^* h_r \right)^*\end{aligned}. \tag{S10.5}$$

Taking its imaginary parts,

$$\begin{aligned}\mathrm{Im}\left(\vec{S}_{0,\theta}\right) &= \mathrm{Im}\left[\left(q e^{-i(m+l)\theta} f_r^* f_z \right)^* \right] - \mathrm{Im}\left(q e^{-i(m+l)\theta} f_r^* f_z \right) \\ &\quad + \mathrm{Im}\left(q e^{-i(m+l)\theta} h_z^* h_r \right) - \mathrm{Im}\left[\left(q e^{-i(m+l)\theta} h_z^* h_r \right)^* \right] \\ &= -2\,\mathrm{Im}\left(q e^{-i(m+l)\theta} f_r^* f_z \right) + 2\,\mathrm{Im}\left(q e^{-i(m+l)\theta} h_z^* h_r \right)\end{aligned}. \tag{S10.6}$$

Again from Eq. (S10.2), we obtain

$$\begin{aligned}\mathrm{Im}\left(\vec{S}_{0,\theta}\right) &= -2\,\mathrm{Im}\left(q e^{-i(m+l)\theta} \frac{-m}{\bar{\rho}} h_z^* f_z \right) + 2\,\mathrm{Im}\left(e^{-i(m+l)\theta} h_z^* \frac{-l}{\bar{\rho}} f_z \right) \\ &= 2\frac{m-l}{\bar{\rho}} \mathrm{Im}\left(q e^{-i(m+l)\theta} h_z^* f_z \right)\end{aligned}. \tag{S10.7}$$

By $\vec{S} = \frac{1}{4}\left(1+|q|^2\right)^{-1} \mathrm{Im}\left(\vec{S}_0\right)$ in Eq. (S9.2), we go back to the spin vector to have

$$S_\theta = \frac{1}{4}\frac{1}{1+|q|^2}\operatorname{Im}\left(\vec{S}_{0,\theta}\right) = \frac{1}{4}\frac{1}{1+|q|^2} 2\frac{m-l}{\bar{\rho}}\operatorname{Im}\left(qe^{-i(m+l)\theta}h_z^* f_z\right)$$
$$= \frac{1}{2}\frac{1}{1+|q|^2}\frac{m-l}{\bar{\rho}} N\left|F_m^\pm\right|^2 \operatorname{Im}\left(qe^{-i(m+l)\theta}\right) \quad . \qquad \text{(S10.8)}$$

As a consequence, $S_\theta = 0$ hold true if and only if two waves propagate in counter-rotations with the same magnitude. This result $S_\theta = 0$ is therefore a very special. By the way, $S_\theta = 0$ corresponds to the in-plane spin being radially polarized.

In dimensional terms, we note that $S_\theta \propto \operatorname{Im}\left(qe^{-i(m+l)\theta}h_z^* f_z\right) \propto \operatorname{Im}\left(H_z^* E_z\right)$, which is nothing but the quantity proportional to the optical chirality in the axial direction. In this respect, we notice that $C \equiv -(2n)^{-1}\operatorname{Im}\left(\vec{E}^* \cdot \vec{H}\right) = (2n)^{-1}\operatorname{Im}\left(\vec{H}^* \cdot \vec{E}\right)$.
